# Conformal graphene coatings on ordinary fabrics for wearable electronic devices

**DOI:** 10.1038/s41467-026-73319-2

**Published:** 2026-05-18

**Authors:** Zibo Chen, Yunfa Si, Xiaobin Liao, Rui Fang, Zhen Li, Weimingyang Tan, Sichang Wang, Yongyi Ji, Wei Qian, Huaqiang Fu, Lun Li, Runquan Li, Mingtao Chen, Bo Liu, Zhugen Yang, Jiaxing Huang, Daping He

**Affiliations:** 1 Sanya Science and Education Innovation Park of Wuhan University of Technology, Sanya, China; 2 School of Materials Science and Engineering, Wuhan University of Technology, Wuhan, China; 3 State Key Laboratory of Advanced Technology for Materials Synthesis and Processing, Wuhan University of Technology, Wuhan, China; 4 Hubei Engineering Research Center of RF-Microwave Technology and Application, School of Physics and Mechanics, Wuhan University of Technology, Wuhan, China; 5 School of Engineering, Westlake University, Hangzhou, China; 6 Faculty of Engineering and Applied Sciences, Cranfield University, Cranfield, UK

**Keywords:** Electronic properties and devices, Electronic devices, Chemical engineering

## Abstract

Dip-coating ordinary fabrics with conductive macromolecules holds promise for mass-production of next-generation wearable electronics but faces an interaction dilemma in high-entangled fabrics: weak interactions for uniform penetration versus strong for stable coating. Herein, we present a temporal decoupling strategy, designing stage-specific interaction strengths to achieve uniform graphene oxide penetration and robust reduced graphene oxide adhesion. Using the triphilic surfactant Triton X-100 as a representative system, this strategy enables the fabrication of fabrics with conductivity (283.1 S m^−1^) and comprehensive wearability (hydrophilicity, air permeability, washability, bacteriostasis, and biocompatibility) over 200-meter roll. This combination of conductivity and production scale outperforms current competitors by over 100-fold, with over 10-time-lower cost (0.4 US$ m^−2^). This strategy is universally applicable to various ordinary fabrics and enables multifunctional applications, including electromagnetic interference shielding and Joule heating. Our work offers a scalable, universal and low-cost methodology for fabric-based wearable electronics with immediate potential for industrial adoption.

## Abstract (web summary)

Chen et al. report a strategy of separating dipping and coating processes for fabricating highly conductive and comprehensive wearable graphene electronic fabrics. This low-cost methodology achieves 200-meter roll mass production, offering a universal platform for next-generation wearable technologies.

## Introduction

Electronic fabrics (E-fabrics), which integrate functional materials into fabrics, hold great potential for creating advanced wearable devices that could revolutionize our interaction with technology^1^. With annual fabrics production exceeding 100 megatons, any attempt to render ordinary fabrics conductive, normally insulating polymer, to E-fabrics must align with scalable production^2^. Meanwhile, it must also fulfil wearability criteria, such as hydrophilicity, air-permeability, washability, bacteriostasis and biocompatibility^3–5^. In top-down fabric level manufacturing (usually presenting a highly entangled structure), dip coating stands out as a potential candidate^6,7^, while effective for small molecules, faces challenges with conductive macromolecules, such as graphene, carbon nanotubes, and conductive polymers^8^. It usually fails because they are hard to disperse and cannot penetrate deep into fabrics and form conformal structure^9^, seriously deteriorating the fabrics’ air-permeability. Even worse, the interactions between macromolecules and polymer are usually weak and cannot maintain performance in daily use and periodic laundry^10^.

To address these challenges, graphene oxide (GO), a precursor to graphene-based materials, has emerged as a promising candidate due to excellent aqueous processability^11,12^ and high surface area^13^. These features enable GO to penetrate fabric networks easily and facilitate conformal coating. However, limited interactions between the GO with polar^14–16^ nature and polymer with aliphatic^17–19^ properties, deteriorate conductivity and durability. Previous efforts^20–26^ enhance interactions self-assemble robust GO coatings and often result in non-conformal structures, such as sandwich structures^27–29^, which covers the gaps among fibers, disrupting fabrics’ air-permeability. These methods struggle to achieve GO penetration and robust coatings simultaneously in liquid phase. Essentially, this reveals an interaction dilemma: GO requires weak interactions to uniformly penetrate complex fabric networks but needs strong interactions to ensure the stability and durability of coatings. Furthermore, upon reduction to reduced graphene oxide (rGO), its conductivity is enhanced due to increased aromaticity^30,31^, but also necessitates strong interactions for maintaining robust coatings. Thus, a designed interaction strategy is required to resolve conflicting requirements orthogonally through temporal decoupling (TD) dip coating process.

**Fig. 1:**
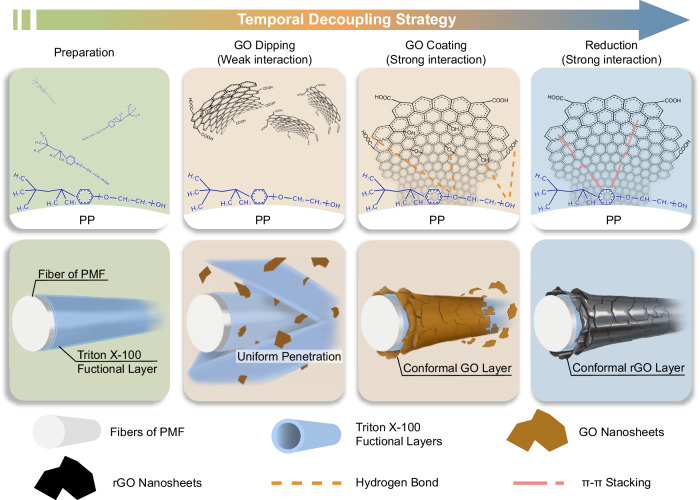
Schematic representation of TD strategy and rGO-PMF synthesis. It shows the interaction at interfaces and microscopic perspectives in preparation, GO dipping, GO coating and reduction.

Herein, we present an innovative TD strategy, which separates dipping and coating processes, designing interaction strengths at different stages. This approach employs Triton X-100 (Triton), a tailored triphilic surfactant, which facilitates GO penetration, enabling robust adhesion and enhanced fabric performance. Polypropylene melt-blown fabrics (PMF), widely used in daily applications, are selected as a challenging model for demonstration due to their chemical stability^32^ and structural complexity^33^. The process is presented schematically (Fig. 1). Before GO dip coating, Triton quickly wets the PMF and forms a functional layer by embedding polypropylene (PP) through its octyl groups under partial swelling. During the dipping process, Triton’s non-ionicity and polyethylene glycol segments allow uniform GO penetration with limited interactions, while strong interactions are formed via hydrogen bonding in the coating process, the drying procedure. Upon reduction to rGO, the hydrogen bonds weaken, while π-π stacking interactions between Triton’s phenyl groups and the rGO coating further stabilize the structure, enhancing conductivity and long-term durability of obtained E-fabrics. Furthermore, TD strategy must achieve an optimal balance between electronic performance and full wearability because the true advance for E-fabrics does not lie in maximizing raw conductivity. Therefore, it is designed to solve this ‘conductivity vs. wearability’ bottleneck, engineering a material that retains full functionality, rather than a material that compromises it for diminishing electronic returns.

We demonstrate this strategy by producing conformal rGO-coated PMF through multiple cycles of GO dip coating and reduction (rGO-PMF-n) in an assembly line. Depth profiling characterizations and molecular dynamics simulations confirm designed interactions that improve GO and rGO penetration and adhesion, ensuring conductivity and long-term stability. The strategy is applicable to multiple non-ionic triphilic surfactants and to various ordinary polymer/natural fabrics, including polyamide and cotton. Notably, rGO-PMF-16 first achieves 283.1 S m^−1^ conductivity over a 200-meter roll production with less than 2.4% relative deviation. It overperforms in conductivity and production scale among competitors by orders of magnitude, with significantly low production cost of 0.4 US$ m^−2^. rGO-PMF-16 shows a potential for universal platform enabling multiple wearable electronics, exceling in full-body applications such as electromagnetic shielding and Joule heating for demonstration, while meeting wearability criteria like hydrophilicity, air permeability, washability, bacteriostasis, and biocompatibility. This work uses an innovative TD strategy to address critical interaction dilemma in massive production, demonstrating immediate potential for industrial adoption of graphene-based E-fabrics and to advance their potential for next-generation wearable technologies.

## Results

### Microscopic characterization

**Fig. 2:**
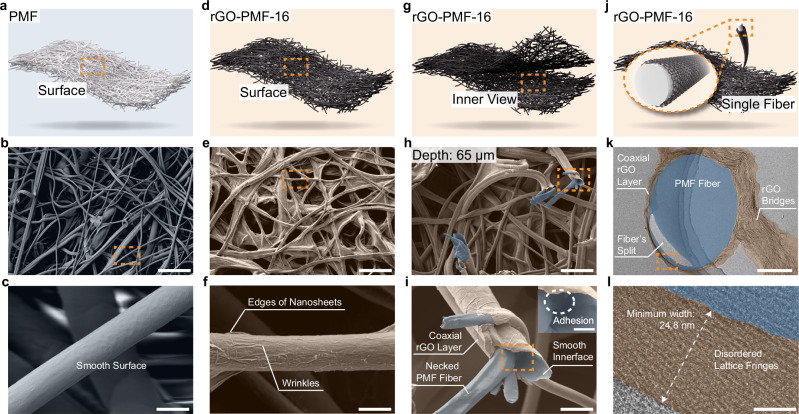
Schematic representation and microscopic characterization of PMF and rGO-PMF. **a** The schematic representation of PMF. **b** The SEM image of PMF. **c** The SEM image of marked part in **b**. **d** The schematic representation of rGO-PMF-16. **e** The SEM image of rGO-PMF-16’s surface. **f** The SEM image of marked part in **e** Fig. 2e. **g** The schematic representation of rGO-PMF-16’s inner view. **h** The SEM image of rGO-PMF-16 at the depth of 65 μm. **i** The SEM image of marked part in **h** Fig. 2h. **j** The schematic representation of rGO-PMF-16’s single fiber. **k** The HRTEM image of rGO-PMF-16’s single fiber section view. **l** The HRTEM image of marked part in **k**. Scale bars: 20 μm **b,** **e**, 2 μm (**c,** **f, i** and **its inset**), 10 μm **h**, 500 nm **k**, and 10 nm **l**.

Conformal rGO layer brings high conductivity and decent wearability for PMF. Microscopic characterization has been conducted to the outcomes where rGO is marked in orange, and PP is marked in blue. A schematic representation of PMF is exhibited (Fig. 2a). The scanning electron microscope (SEM) image shows the non-oriented fiber structure with a range of diameter from 1 to 5 μm (Fig. 2b). A closer inspection demonstrates the smooth surface of PMF fibers during melt-blown manufacturing (Fig. 2c). While after 16 cycles of dip coating treatment and reduction, fibers are coated by conformal rGO layer on the surface and rGO bridges connect fibers at the cross sites in rGO-PMF-16 (Fig. 2d, e). Besides, fibers bundle together, creating inter-bundle gaps that confer air-permeability. Bridges and bundling structures also create shortcuts of electron pathway across the fibers for enhancing conductivity. A series of SEM images show how rGO nanosheets shape rGO-PMFs by different cycles of dip coating treatment (Supplementary Fig. 1). Moreover, a surface of single fiber is zoomed in where wrinkles and edges of rGO nanosheets are observed (Fig. 2f), proving the conformal rGO layer. Wrinkles across fibers also emerge to support the bundling structure (Supplementary Fig. 2).

Excluding the surface, an inner view is indispensable for uniformity in depth (Fig. 2g). Cryosection technology exposes the structures at the depth of 65 μm (Fig. 2h). rGO-PMF-16 maintains morphology as the surface shown above. Notably, conformal rGO layer is confirmed at the fracture sites. A detailed image reveals ductile fracture (Fig. 2i). Necked PMF fibers pose at the axis of conformal rGO layer and do not break at the same site as rGO, which helps observing the inner face of fibers. The inner face of conformal rGO layer is smoother compared to the outside surface, which the iconic rGO wrinkles are covered by a residual layer. At a larger magnification, an adhesion part between necked PMF and the residual layer appears clearly. It seems that strong interactions exist at the interface between PP and conformal graphene-based layers.

To investigate the interaction mentioned, it is necessary to analyze single fiber interfaces in detail (Fig. 2j). Thus, the advanced cryo-ultramicrotomy technology is conducted along with high-resolution transmission electron microscopy (HRTEM) for observing the cross section of a single rGO-PMF-16 fiber (Fig. 2k). Conformal rGO layer forms entirely around the PMF fiber, which also sprouts rGO bridges for connecting other fibers nearby. The conformal rGO layer exhibits disorientation of layered stacking. A split is generated inside of the PMF fiber by cryo-ultramicrotomy, while the inner side of it attaches a PP layer with thickness of 50–80 nm. This phenomenon confirms that strong interactions exist because the fracture emerges first inside of PP rather than the interface of conformal rGO layer. These results benefit washability and durability. Furthermore, the magnification of marked part demonstrates the minimum thickness of conformal rGO layer with disordered lattice fringes is 24.8 nm (Fig. 2l). The lattice fringes indicate moderate reduction of rGO, which is expected to simultaneously realize conductivity and hydrophilicity. Finally, cryogenic focused ion beam SEM (Cryo-FIB-SEM) characterization on a large-diameter PP fiber confirms that the interfacial micromorphology remains consistent regardless of fiber diameter (Supplementary Fig. 3).

### Mechanism and its universality

**Fig. 3:**
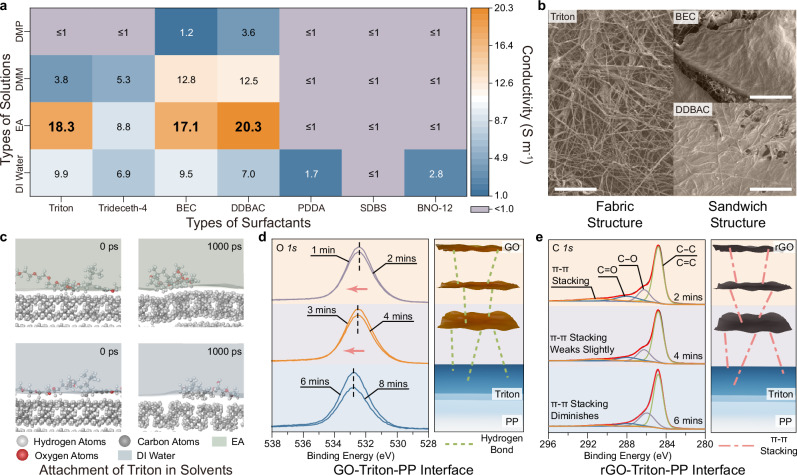
The mechanism of TD strategy. **a** The heat map of conductivity with single dip coating treatment in different surfactant-solution combinations. **b** The SEM image of rGO-PMF-1 assembled by Triton, BEC and DDBAC. **c** The schematic representation of Triton’s attachment in molecular dynamics simulation. **d** The XPS spectrum of O *1 s* GO-Triton-PP interface and its schematic representation. **e** The XPS spectrum of C *1 s* rGO-Triton-PP interface and its schematic representation. Scale bars: 100 μm **b**.

The mechanism and universality of the TD strategy require investigation. The optimization of the strategy is also worthy of discussion, although optimized manufacture processes are supported by Fourier transform infrared (FTIR) absorption spectroscopy and X-ray diffraction (XRD) patterns (Supplementary Fig. 4). Considering that the construction of functional Triton layer in preparation process is influenced by surface free energy and solubility parameters of solutions, deionized (DI) water, ethyl acetate (EA), dimethyl malonate (DMM) and dimethyl phthalate (DMP) are selected as solvents. Triton, trideceth-4, benzethonium chloride (BEC), benzyldimethyl-dodecylammonium chloride (DDBAC), poly dimethyl diallyl ammonium chloride (PDDA), sodium dodecyl benzene sulfonate (SDBS) and naphthol polyoxyethylene ether (BNO-12) are tested as surfactants. Surface free energy difference values (D-values) of PP and different solutions have been measured and calculated (Supplementary Fig. 5) with original data demonstrated (Supplementary Table 1). Their conductivity results of single dip coating treatment are measured into a heat map (Fig. 3a). By vertically comparing the data, it is observed that larger D-values result in more wetted surface in mixed solution^34^, which enhances efficiency of surface functionalization, thereby improving conductivity and making EA the preferred choice. Besides, notable reversible swelling caused by closer solubility parameters of EA and PP (Supplementary Fig. 6) embeds Triton’s octyl groups into PP and enhances Triton’s attachment, providing abundant hydrogen bond sites of GO nanosheets.

The penetration of GO requires optimal size and weak interactions. First, the size effect is crucial to penetrating the inner part of PMF. As sizes of the nanosheets increase, electron flow through coatings becomes more continuous, resulting in higher conductivity, which, however, is more difficult for GO penetration. The peak of conductivity in cycles dipping is detected, which appears at GO’s average size of 401.5 nm (Supplementary Fig. 7), with single layers (Supplementary Fig. 8) and GO size distribution analysis (Supplementary Fig. 9). Furthermore, the weak interaction during penetration depends on the ionicity of surfactants (Supplementary Table 2) as well, considering GO nanosheets express negative ionicity. Horizontally comparing the data in Fig. 3a, SDBS, as an anionic ionic surfactant, shows unsatisfactory conductivity data due to repulsion. In contrast, the highest conductivities are observed with the EA solution containing BEC, DDBAC, and Triton. However, the cationic surfactants, BEC and DDBAC, are eliminated because their interactions lead to formation of sandwich-like GO structures, which covers the gaps between fibers and disrupts air permeability. While Triton, a non-ionic surfactant, maintains a conformal fabric structure (Fig. 3b). Besides, the selection of Triton and its derivatives is discussed, and Triton X-100 is the optimal choice (Supplementary Table 3).

Robust conformal GO and rGO coatings demand strong interactions. According to the highest 3 conductivity data mentioned, those surfactants share a commonality which is triphilic property (aliphatic-polar-aromatic affinity) and the affinities of all the surfactants are listed (Supplementary Table 4). To identify the aliphatic affinity of Triton in interface, molecular dynamics simulations are conducted, indicating that Triton attaches from EA to the surface of PP and maintains attachment in DI water (Supplementary Video 1 and 2). Triton molecules maintain attachment during 1000 ps in DI water and EA (Fig. 3c and Supplementary Fig. 10), which show potentials for subsequent entanglement through swelling and provides polar and aromatic affinities to the interface. X-ray photoelectron spectroscopy (XPS) depth profiling further reveals the existence of strong interactions at the interface. For robust GO coatings, the results of O *1 s* in GO-Triton-PP interface in 8 mins of Ar gas cluster ion beam (GCIB) are presented and analyzed (Fig. 3d). The data can be divided into 3 parts, which are GO-GO interface in 1-2 mins, GO-Triton interface in 3-4 mins and Triton-PP dispersion after 6 mins. As the etching progresses, the intensity of O *1 s* decreases, while peaks stay still in each phase. Specifically, the stronger intensity of hydrogen bonds leads to the higher electron cloud density around oxygen atoms, and the lower binding energy of them. Notably, hydrogen bonding was observed at the GO-GO and GO-Triton interfaces, which proved to be shifted towards lower binding energies at these two interfaces with 532.4 eV/532.5 eV, respectively, as compared to the binding energy peak of 532.8 eV in the Triton-PP dispersion. Besides, the peak’s existence and weakening after 6 mins indicate Triton effectively enters PP by swelling because Triton is the only resource of oxygen. For durable rGO coatings, the results of C *1 s* in rGO-Triton-PP interface in 6 mins of Ar GCIB are provided (Fig. 3e), where rGO-rGO interface is at 2 mins, rGO-Triton interface is at 4 mins and Triton-PP dispersion after 6 mins. As the etching progresses, the classification of the 3 phases is supported by the initial decrease and subsequent increase in C-O peaks, along with the continuous decline of C = O peaks. The C = O peaks are attributed solely to rGO, while C-O peaks appear in both rGO and Triton. Additionally, the π-π satellite peaks weaken slightly at the rGO-Triton interface and disappear in the Triton-PP dispersion, indicating the presence of π-π stacking interactions, which are reduced in the rGO-Triton interface compared to the rGO-rGO interface. In brief, the hydrogen bond and π-π stacking separately induced from TD strategy in different processes contribute to robustness of E-fabrics.

**Fig. 4:**
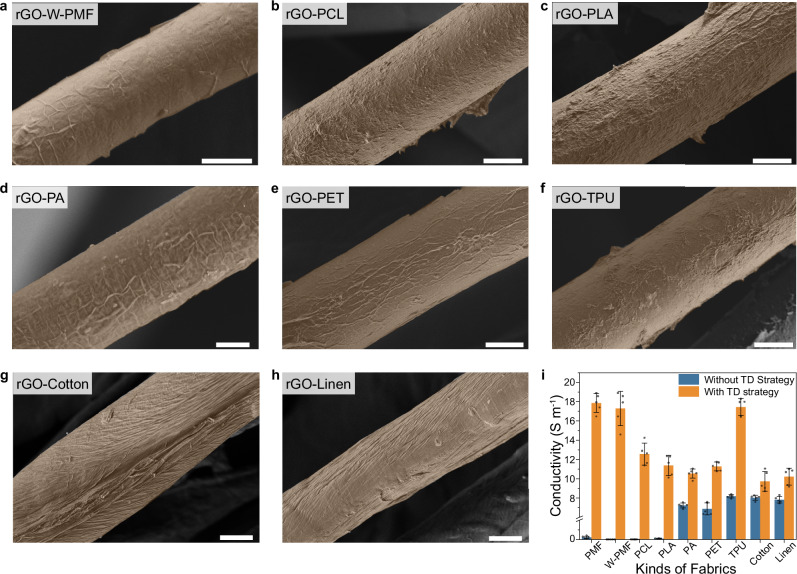
The universality of TD strategy for different ordinary fabrics in single dip coating treatment. **a-h** The SEM images of different rGO conformal coated ordinary fabrics. rGO coating fabrics are colored orange in this figure. **i** The conductivity of different rGO conformal coated ordinary fabrics with/without TD strategy. Data is presented as mean ± standard deviation. The sample size (*n* = 5) represents five independently prepared and tested fabric samples for each group. Scale bars: 1 μm **a,** **d,** 5 μm **b,** **c,** **e, f, g** and **h**.

Our TD strategy is not limited to any specific surfactant or certain fabrics. Since Triton and its derivatives may cause environmental concerns, we ensure the world-wide compliance of rGO-PMF-16 (Supplementary Fig. 11) and successfully replicate the synthesis of conformal rGO coatings by cardanol polyoxyethylene ether with a superior environmental profile (Supplementary Fig. 12). Furthermore, the universality of TD strategy requires study for graphene on various ordinary polymer/natural fabrics. We execute single dip coating treatment on 8 kinds of fabrics and assemble various E-fabrics named rGO-X (Fig. 4). And those polymer fabrics are not surface pre-functionalized, proven by XPS (Supplementary Fig. 13). We tested Wasted PMF (W-PMF), along with polycaprolactone (PCL) and polylactic acid (PLA), as non-polar polymer fabrics, shows similar significant enhancements as PMF with TD strategy. While polyamide (PA), polyethylene glycol terephthalate (PET) and thermoplastic polyurethane (TPU) as polar polymer fabrics, improve their conductivity compared to the outcomes without TD strategy. Natural fabrics show similarity as polar polymer fabrics as well. Clearly, iconic surface morphology of graphene appears on rGO-fabrics in conformal coating structure compared to their raw surface (Supplementary Fig. 14). And the variation of conductivity among multiple fabrics microstructure of the polymer fabric scaffold (Supplementary Fig. 15 and Supplementary Table 5). Overall, it is quite promising for large-scale producing E-fabrics through TD strategy.

### Massive production and applications

**Fig. 5:**
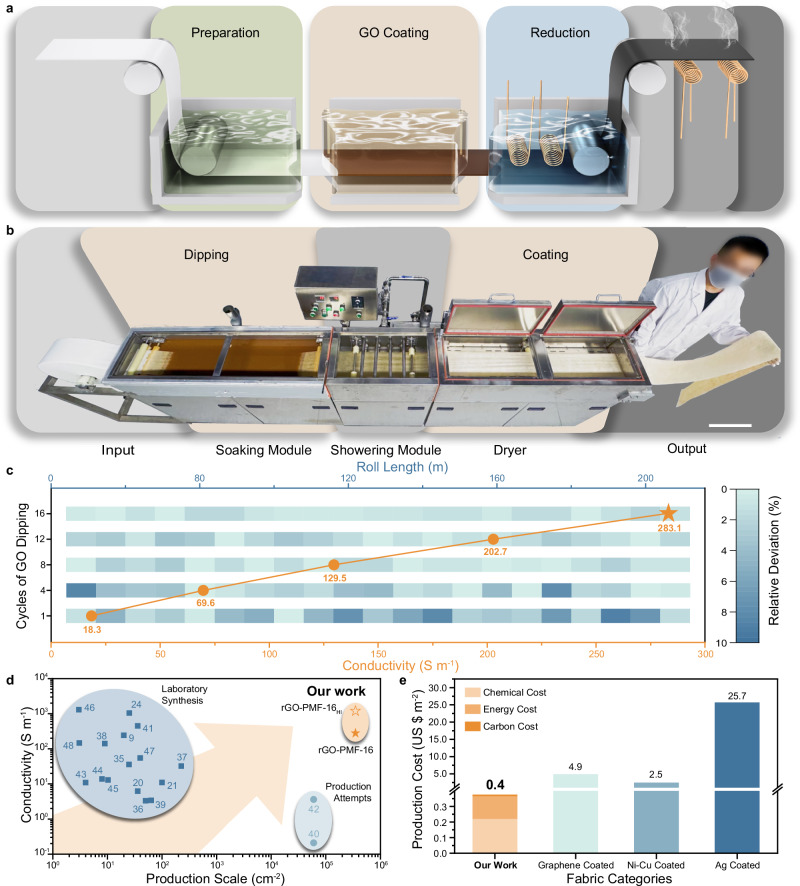
Massive production of rGO-PMF-16. **a** The schematic representation of the pilot assembly line. **b** The optical photo of massive production in GO dipping and coating processes. **c** The conductivity of 200 m roll for multiple rGO-PMF and their relative deviation. **d** The comparison of graphene-based fabrics in conductivity and production scale. **e** The production cost comparison of rGO-PMF-16 and other commercial conductive fabrics. Scale bars: 20 cm **b**.

We integrate the preparation, GO coating, and reduction processes into an orderly liquid-phase processing assembly line for massive production (Fig. 5a). Manufacturing parameters have been optimized (Supplementary Fig. 16-19) in a pilot assembly line (Fig. 5b) for high conductivity and decent wearability. Strikingly, we produce 200 m long roll of rGO-PMF with different cycles of GO dipping and achieve the highest conductivity of 283.1 S m^‒1^ under 16 cycles of dipping with a relative deviation of less than 2.4% (Fig. 5c). Obviously, conductivity increases nearly linearly with the increase of dipping cycles, while the relative deviation gradually decreases, which also indicates an increasingly stable tendency of conductivity fluctuation in production. And this stable tendency appears in different environmental conditions as well (Supplementary Fig. 20). To elucidate the underlying conductive mechanism, we further characterize the single-fiber conductivity and z-directional transport, confirming the establishment of a robust 3D conductive network enabled by continuous coaxial pathways (Supplementary Fig. 21 and 22). To benchmark this performance and test the conductivity-first paradigm, we also fabricated an aggressively reduced sample (rGO-PMF-16_HI_) using hydroiodic acid, achieving a conductivity of 1214.8 S m^‒1^. While this represents a 4.3-fold increase in conductivity, it provides diminishing returns. More critically, this aggressive reduction compromises essential wearability: the fabric becomes hydrophobic and loses its bacteriostatic properties (discussed later in Supplementary Fig. 29). This quantitatively demonstrates that our rGO-PMF-16 represents an optimal balance for wearable applications, solving a key bottleneck in the field (Supplementary Fig. 23 and Supplementary Table 6). Due to its ultra-high conductivity and extensive production capacity, rGO-PMF-16 clearly outperforms competitors^9,20,21,24,35–48^ (Fig. 5d) over 100 times. Additionally, our production cost of only 0.4 US$ m^‒2^ is more than 10 times lower commercial products (Fig. 5e) with detailed information listed (Supplementary Table 7 and 8), highlighting its strong potential for immediate industrial adoption.

**Fig. 6:**
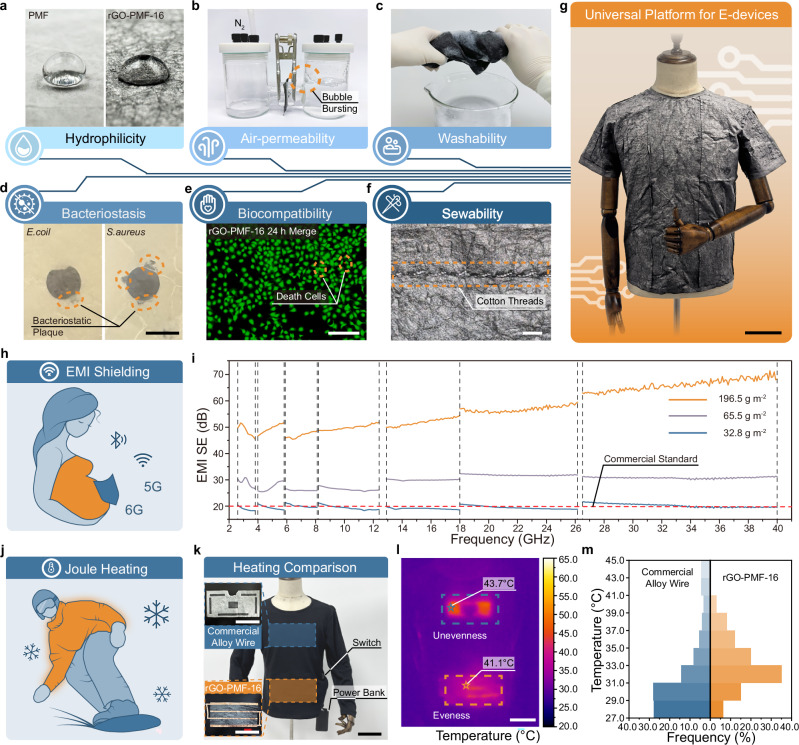
Multiple wearability and potential as universal platform for E-devices. **a** The optical image of PMF’s and rGO-PMF-16’s hydrophilicity. **b** The optical image of rGO-PMF-16’s air-permeability. **c** The optical image of washing rGO-PMF-16. **d** The optical image of *Escherichia coli* and *Staphylococcus aureus* cultivated 8 h with rGO-PMF-16. **e** The fluorescence microscopy merged image of human fibroblasts cultivated 24 h with rGO-PMF-16. **f** The optical image of a suture linked two pieces of rGO-PMF-16. **g** The optical image of a T-shirt made by rGO-PMF-16. **h** The schematic representation of electromagnetic shielding in daily life. **i** The EMI SE properties of rGO-PMF-16. **j** The schematic representation of Joule heating in daily life. **k** The optical image of alloy wire and rGO-PMF-16 on commercial heating clothing **l**. The infrared image of alloy wire and rGO-PMF-16 on commercial heating clothing **m**. The temperature distribution of marked parts in Fig. 6l. Scale bars: 1 cm **d**, 50 μm **e**, 5 mm **f**, 10 cm **g,** **k** and **l**.

For wearable electronic devices (E-devices), wearability is another crucial property that needs to be considered. rGO-PMF-16 shows hydrophilicity by moderate reduction while PMF is hydrophobic (Fig. 6a). It partially removes oxygen-containing functional groups and obtains contact angles below 90˚ (Supplementary Fig. 24). Especially, Triton treated PMF expresses excellent hydrophilicity where water drops absorb within 366 ms (Supplementary Fig. 25). Air-permeability is observed as well, by bubble bursting on the right side of H-type electrolytic cell concretely under external N_2_ flow (Fig. 6b). It benefits from the conformal coating structure where rGO-PMF-16 achieves 103.7 mm s^‒1^ (Supplementary Fig. 26). Simulation of daily washing is executed with washing powder (Fig. 6c). Excellent washability is confirmed within 2.3% of weight loss and 4% of conductivity deterioration (Supplementary Fig. 27a) with excellent long-term durability (Supplementary Fig. 27b). Crucially, we confirm negligible environmental risk from nanoparticle release through rigorous multimodal analysis, including dynamic light scattering and theoretical exposure modeling (Supplementary Fig. 28 and Supplementary Table 9).

Furthermore, wearable E-devices based on E-fabrics require bacteriostasis and biocompatibility as well. Bacteriostasis is examined with *Escherichia coli* (*E. coli*) and *Staphylococcus aureus* (*S. aureus*) (Fig. 6d). While *E. coli* partially obscures the rGO-PMF-16 with concurrent bacterial plaques, *S. aureus* growth is significantly inhibited. Oscillatory liquid culture, simulating dynamic wearing conditions, yields consistent results, showing higher efficacy against *S. aureus* (Supplementary Fig. 29). These differences are attributed to distinct cell wall structures and their varying susceptibility to the nanoknife and glutathione oxidation effects^49^. Biocompatibility is assessed using human fibroblasts (HFB) cultivated for 24 h (Fig. 6e). Fluorescence microscopy reveals excellent biocompatibility, with only negligible dead cells (red spots) observed in the field of view. High cell survival rates over 24, 48, and 72 h further confirm this cytocompatibility (Supplementary Figs. 30 and 31). To comprehensively validate safety, we further demonstrated that rGO-PMFs exhibit negligible cytotoxicity to immune cells (macrophages), strongly supporting their biocompatibility for long-term skin-contact applications (Supplementary Fig. 32-34).

Convenient processibility is crucial for E-devices on the backend. The sewability is proved and the marked part shows how cotton threads sew two pieces of rGO-PMF-16 fabrics with a sewing machine (Fig. 6f). Then we make a T-shirt for body size with rolls of rGO-PMF-16 fabrics to show its scalable processibility (Fig. 6g). Obviously, the obtained multiple properties make it promising as a universal platform for assembling different E-devices. Therefore, we showcase wearable electromagnetic interference (EMI) shielding for different modern communication bands (Fig. 6h). EMI shielding effectiveness (EMI SE) is detected for 1 (32.8 g m^‒2^), 2 (65.5 g m^‒2^) and 6 layers (196.5 g m^‒2^) of rGO-PMF-16, from 2.6 to 40.0 GHz comprising Bluetooth, Wi-Fi, 5G and the upcoming 6G frequency bands (Fig. 6i). The performance of EMI SE reaches above 50.0 dB in X band and obtains the highest EMI SE of 71.0 dB near 40.0 GHz, with the weight of 196.5 g m^‒2^ which is as lightweight as cotton T-shirts for summertime. A single layer of 32.8 g m^‒2^ still meets the commercial standards. Excellent EMI SE performance is because conformal rGO layer reflects the microwaves between fibers and generates induced current to dissipate energy. However, the further increasing conductivity yields diminishing returns ( < 5 dB gain) while severely compromising other wearable properties (Supplementary Fig. 35).

We also showcase the Joule heating to prove rGO-PMF-16 as universal platform (Fig. 6j). rGO-PMF-16 in same size replaces a commercial alloy wire heating module in commercial heating clothing with the original circuit powered by a portable power bank (Fig. 6k). After switching on, the temperature goes higher in several minutes and shows special patterns in the infrared image (Fig. 6l). It is easy to tell that the evenness of each varies a lot. rGO-PMF-16 heats a larger area with better evenness, while its hottest point is close to that in the commercial alloy wire heating module under the same voltage. Detailed analysis is performed and tells the differences of heating outcomes (Fig. 6m). The commercial alloy wire demonstrates a wider distribution in the low/high temperature ranges, while rGO-PMF-16 shows a more centralized temperature profile. This indicates the superiority of rGO-PMF-16 because conformal rGO coated fibers in disordered structure provide moderate and even resistance for Joule heating. Besides, this structure assembles a huge heat conduction network for temperature equalization. With all performance mentioned above, we believe rGO-PMF-16 will be the next universal platform for multiple advanced wearable E-devices.

## Discussion

The TD strategy introduced in this work provides a transformative solution to the persistent challenge of achieving conformal and durable coatings on highly entangled fabric structures. By strategically engineering different interaction strengths at distinct stages of the dip-coating process, this method not only significantly enhances conductivity but also, critically, preserves essential wearability properties. This work re-frames the field’s goal from maximizing raw conductivity to achieving an optimal balance of electronic and wearable functions. We quantitatively demonstrate that our 283.1 S m^‒1^ fabric achieves this balance, whereas an aggressively reduced 1214.8 S m^‒1^ version suffers from diminishing electronic returns ( < 5 dB EMI gain) and compromised wearability (e.g., loss of hydrophilicity and bacteriostasis). This combination of a breakthrough strategy, proven industrial-scale production (200-meter roll with < 2.3% deviation), and ultra-low cost (0.4 US$ m^‒2^) underscores its universal applicability and strong potential for immediate industrial adoption.

Looking forward, further optimization of interfacial interaction dynamics will expand the applicability of this technique to a wider range of fabrics and conductive materials, such as carbon nanotubes and conductive polymers. The scalability of this process for mass production, along with its environmental sustainability, will be crucial for the commercialization of graphene-based electronic textiles. Ultimately, this strategy lays a solid foundation for the next generation of flexible, high-performance smart textiles, opening diverse applications in healthcare, energy, and beyond.

## Methods

### Reagents

All the reagents were purchased from Shanghai Aladdin Biochemical Technology Co., Ltd. and used as received without further purification. Polymer/natural fabrics were purchased from Shandong Changbode Holdings Group Nonwoven Polyolefin Research Institute, China. Graphene Oxide (GO) filter cake was purchased from Wuhan Hanene Technology Co., Ltd, China.

### Synthesis of conformal graphene coating fabrics

GO aqueous dispersion: GO filter cake is diluted with DI water to a concentration of 1 wt% in a vertical agitator and stirred at a speed of 800 rpm overnight. Subsequently, the dispersion enters the stirring defoamer for further dispersion, with 2 cycles of 2000 rpm for 28 min and 2200 rpm for 2 min. The obtained dispersion is transferred to high pressure homogenizer with 0, 400, 800, 1200, and 1600 bar to acquire different diameters of GO nanosheets. Followed by this, the dispersion is diluted into 0.5 wt% with DI water and treated in the stirring defoamer for another 2 cycles with the same parameters. After these processes, GO aqueous dispersion is stored at 4 °C for further usage.

Fabric functionalization: Polymer/natural fabrics are purchased in rolls and put into the pilot assembly line. Medical alcohol is added to the soaking module and the ultrasonic cleaning function is activated. Fabrics are transported at a certain speed and dried at 75 °C through a drying module to ensure that they undergo 30 min of processing in each module. After these processes, fabrics are wrapped into rolls for functionalization. A combination of solvents and surfactants is configured with a concentration of 5 wt‰ for Triton and poured into the soaking module at room temperature. Rolling speed is turned down to ensure that fabrics undergo a series of time for partial swelling. Soaked fabrics are transported at the same speed and dried at 45 °C. After these processes, fabrics are wrapped into rolls for further usage. Other functionalized PMF with other surfactants are synthesized in the same method.

GO coating: GO aqueous dispersion with different parameters is poured into soaking module and stirred at 200 ml min^‒1^ with a pump. Functionalized polymer/natural fabrics are put into the pilot assembly line at a series of speed followed by drying at 55 °C in dry module and then wrapped into rolls, marked as GO-PMF-n (n stands for cycles of GO dip-coating). Rolls of GO-PMF-n are put back to the pilot assembly line with the same parameters for more cycles of dip-coating.

Reduction of GO coatings: We prepare a 0.1 mol L^‒1^ thiourea dioxide aqueous solution and pour the solution into the soaking module. The solution was adjusted to pH 9.5-10.0, stirred at a certain speed of 200 ml min^‒1^ and heated to 85 °C. The coated fabrics are put into the pilot assembly line and set at different speeds for different reduction time. After that, fabrics are set to the washing module for DI water showering and then dried in the dry module at 55 °C. Finally, rGO coated fabrics are wrapped into rolls for further usage.

### Material and structural characterization

Cryosection: Cryosection is conducted to expose the inner view of rGO-PMF-16 by a cryostat (Thermo Fisher HM525 NX). We use FSC 22 (Leica FSC 22 frozen section media) as embedding agent to conduct cryosection. rGO-PMF-16 is shaped into a piece of 2*2 cm. The freeze temperature is set at -35 °C. We embed rGO-PMF-16 with FSC 22 and freeze it on the sample stage. When the freezing is completed, we keep the temperature to slice and record the progress of cryosection process. The first appearance of rGO-PMF-16 in the slice is regarded as the starting point. We set the cutting parameter to 1 μm per slice and repeat 65 times. The remaining samples on the sample stage are thawed, cleaned, and transferred to the SEM for observation.

SEM: SEM images are captured using a dual beam electron microscope (Zeiss Crossbeam 350). Fabric samples are applied onto adhesive carbon tapes attached to aluminum sample stages. GO aqueous dispersion samples are diluted with ethanol into the weight concentration of 2.5 ppm. The diluted solution is then dropped twice onto the tilted gold-coated silicon wafer after plasma treatment, 5 μl each time, which can effectively avoid the coffee ring effect in the process. All samples are coated without any conductive materials via ion sputtering for the real morphology. The SEM images are captured with a working distance of 1.5-5 mm and an accelerating voltage of 0.8-2 kV with an SE2 detector and aperture of 7-30 μm.

Cryo-ultramicrotome: Cryo-ultramicrotome is conducted to detect the single fiber’s section morphology of rGO-PMF-16 by cryo-ultramicrotome (Leica EM UC7 ultramicrotome with EM FC7 Cryochamber). We use EMbed 812 (Electron Microscopy Sciences EMbed 812 embedding kit) to embed rGO-PMF-16. The obtained resin block is trimmed at room temperature to match ultramicrotome. Then we cool it down to -90 °C in ultramicrotome and maintain it for a period. Under the operation of an electrostatic gun, we use a diamond knife to cut resin blocks into thin slices below 100 nm. Then the obtained slices are transferred onto a copper mesh for HRTEM observation.

HRTEM: Samples for HRTEM are prepared transferring a drop of frozen sucrose solution onto 230-mesh microgrid copper meshes. HRTEM images are collected using a JEOL-F200 equipped with a Cs probe aberration corrector, XMAX 100 EDX detector (Oxford Instruments), high-angle annular dark-field and segmented detectors. The system was operated at 300 kV.

XPS depth profiling: XPS depth profiling is conducted to investigate the interactions in GO-Triton-PP interface and rGO-Triton-PP interface. Since the limitation of X-ray beam size, we use an alternative method to analysis. PMF is washed by the same methods reported as experimental section in supplementary information. Washed PMF is put in hot press machine with a 50 μm thick mold at 115 °C for reshaping into PP membrane. The obtained PP membrane is treated by similar methods reported as experimental section in supplementary information, to get PP membrane with 1 dip coating treatment of GO and rGO. GO coated PP membrane and rGO coated PP membrane are examined with XPS instrument (PHI VersaProbe 4). Samples are etched with Ar GCIB for 30 s to eliminate surface adsorbed impurities and XPS analysis is performed every minute of etching, where Ar GCIB recipe is chosen at 10 KV, 30 nA. The X-ray resource is Al mono working at 25.0 W with the beam diameter of 100.0 μm. The neutralizer of the experiment is set at 0.3 V, 5.0 μA with analysis mode is preferred as fixed analyzer transmission mode. The C *1 s* spectrum of carbon and the O *1 s* spectrum of oxygen use the same set of parameters where pass energy is 112 eV and energy step is 0.1 eV. Charge (binding energy scale) calibration is performed using C *1 s* at 284.8 eV.

FTIR: FTIR spectra are recorded on a Nicolet 60-SXB IR instrument in the attenuated total reflection. XRD: XRD patterns are acquired on a Rigaku Smartlab using Cu Kα (λ = 1.5406 Å) radiation and a scanning speed of 3˚ min^‒1^. AFM: The morphology of GO nanosheets is collected by atomic force microscope (Asylum Research Cypher ES).

Surface free energy: Surface free energy is detected for different surfactant solvent solutions. For a solution, surface free energy equals surface tension, thus Wilhelmy plate method is utilized with a rough platinum plate as the probe, in KRÜSS Scientific Tensíío at room temperature automatically.

Soxhlet extraction: A Soxhlet extraction method is employed for sample pre-treatment. rGO-PMF-16 samples (*n* = 3) are accurately weighed at 3.00 g and placed into the thimble of a Soxhlet extractor. Ethyl acetate is used as the extraction solvent, and the samples are refluxed at 90 °C for 24 h to ensure complete dissolution of Triton from PMF matrix. After extraction, the collected ethyl acetate extract is concentrated to near dryness under reduced pressure using a rotary evaporator at 45 °C. To ensure complete removal of the extraction solvent, the residue is co-evaporated with the HPLC mobile phase (9:1 methanol/DI water, ~50 mL). This co-evaporation process (re-dissolving the residue in ~50 mL mobile phase followed by rotary evaporation to near dryness) is repeated for a total of 5 cycles. Following the final evaporation, the residue is quantitatively washed and transferred to a 25 mL volumetric flask using the mobile phase and diluted to the mark. Prior to HPLC analysis, an aliquot of the solution was filtered through a 0.22 μm polytetrafluoroethylene (PTFE) syringe filter.

HPLC: The quantitative analysis of Triton is performed on a Dalian Elite Analytical Instruments Co., Ltd. HPLC system equipped with a UV-visible detector. Chromatographic separation is achieved on an Elite C18 column (column no. E3420776) packed with Supersil ODS2 (4.6×250 mm, 5 μm). The mobile phase consists of methanol and DI water at a volume ratio of 9:1 (v/v). An isocratic elution mode is used at a constant flow rate of 1.2 mL min^‒1^ by a P3100 high-pressure constant-flow pump. The column oven temperature is maintained at 30 °C, and the injection volume is 10 μL. The detection wavelength for Triton is set at 230 nm.

### Molecular dynamics simulation

MD simulations: Classical MD simulations are performed using the FORCITE package in Materials Studio software 2020. All the simulations (bonded and non-bonded) parameters are obtained from the force-field of condensed-phase optimized molecular potentials for atomistic simulation studies (COMPASS Ⅲ). The electrostatic and long-range van der Waals interactions are described using the Ewald summation method. The systems are equilibrated within the NVT ensemble with a time step and a total time of 1 fs and 2 ns, respectively. Nose-Hoover thermostats are carried out to control the temperature and pressure for dynamical simulations. The temperature is set to 300 K for all processes.

### Evaluation of wearability

Hydrophilicity: Contact angles are measured by static contact angle technology, capturing images of the droplet by KRÜSS Scientific DSA30B. The drop is DI water and images are captured after the droplet is stable on the sample surface. Contact angles are fitted automatically with an appropriate algorithm.

Air-permeability: Samples are shaped into round specimens with a diameter of 13 cm and set on AirPerm air permeability tester (SDL Atlas M021A) for air-permeability data under the air flow of 90 L min^‒1^.

Washability: Samples are shaped into round specimens with diameter of 5 cm, washed in a 1 L beaker with 200 mL water with 0.5 wt% of commercial washing powder and stirred at the speed of 300 rpm. Samples are washed for 20 mins, then rinsed for 20 mins at same speed with new tape water and rinsed for 10 mins again to simulate daily washing. Washed sample is hung to dry at 55 °C, after which the residual conductivity and mass retention are measured. To evaluate the potential release of nanomaterials, the washing supernatant is collected for Dynamic Light Scattering (DLS) analysis (Zetasizer Lab, Malvern Panalytical). The parameters are set as follows: rGO (Refractive Index = 2.420, Absorption = 1.000); Dispersant (Water: Temperature = 25.0 °C, Viscosity = 0.8872 mPa s, Refractive Index = 1.330). Additionally, the washing effluent is filtered through a 0.22 μm membrane, and the filtration residue is characterized by SEM.

Bacteriostasis: *Escherichia coli* (*E. coli*) and *Staphylococcus aureus* (*S. aureus*) are used as experimental bacteria. For in vitro antibacterial assays, standard bacterial strains including *E. coli* (BNCC133264) and *S. aureus* (BNCC186335) are utilized and obtained from BeNa Culture Collection (Beijing, China). After the bacteria are cultured to the logarithmic growth stage, 50 μl of bacteria solution are evenly coated on the solid culture dish. The samples (discs with a diameter of 1.0 cm) are attached to a petri dish coated with bacterial solution and cultured inversely at 37 °C for 24 h. The concentration of the bacterial solution is adjusted to 5×10^5^ cfu mL^‒1^, and 10 mL bacterial solution is taken into the culture bottle, and samples (5 cm long and 2 cm wide) are added to the culture bottle. After 24 h of shock culture, the absorbance of the bacterial solution is measured with a microplate reader.

Biocompatibility: Human fibroblasts (HFB) and macrophages are selected for cytotoxicity tests. HFB (HSF, STM-CL-5176, Male, Adult) are obtained from Stemrecell (Shanghai) Biotechnology Co., Ltd. Mouse macrophages (RAW 264.7, CL-0190, Male, Adult) are obtained from Wuhan Pricella Biotechnology Co., Ltd. Cells are cultured in 96-well plates for a series of time, the original medium is removed, and the experimental group continued to culture with material extract instead of complete medium for a series of time. After the culture, cck-8 kit is used to detect cell viability, and the absorbance of cells at 450 nm is detected with enzyme marker. The cells are stained with AM/PI fluorescence.

### Electronic properties and device applications

Electrical conductivity: Conductivities were measured using a four-probe resistivity meter (RTS-9). To assess the uniformity of the continuously produced 200 m rGO-PMF roll, sheet resistance was monitored every 20 m along the longitudinal direction at three transverse positions (center and 5 cm from each edge). The bulk electrical conductivity (σ) was calculated according to the equation,

1 $$\sigma=\frac{1}{{R}_{{sq}}\cdot t}$$

where *R*_*sq*_ is the sheet resistance and *t* is the fabric thickness.

Furthermore, to verify the 3D conductive network, both the z-directional conductivity and the single-fiber conductivity were characterized using a two-/four probe method via Linear Sweep Voltammetry (LSV) on an electrochemical workstation (CHI660E, CH Instruments).

EMI shielding: The EMI SE is measured by the PNA Vector Network Analyzer (N5244B) in the band frequency range of 2.6-40.0 GHz using the waveguide method. All samples are cut into different rectangular shapes for different clamping requirements and sandwiched tightly between the waveguide sample holders. The incident power of electromagnetic waves is 0 dBm, corresponding to 1 mW. Tighten samples before measurement to avoid any leakage at the waveguide edge.

Joule Heating: Obtained rGO-PMF-16 is assembled with copper tapes for the heating module. Then the original circuit is applied to the copper tape to ensure that the heating module is powered for Joule heating by a commercial power bank. Last, infrared camera (Testo 869) is used for capturing infrared images with infrared emissivity being set at ε = 0.8 for cotton.

### Statistics and Reproducibility

No statistical method was used to predetermine sample size. No data were excluded from the analyses. The experiments were not randomized. The investigators were not blinded to allocation during experiments and outcome assessment. All morphological characterizations (including SEM and TEM) and in vitro biological assays (antibacterial tests and live/dead cell staining) were repeated independently at least three times with similar results. Sample sizes were listed in corresponding figure legends and were chosen based on standard practices in the field to ensure adequate statistical power and reproducibility. Representative images from these independent replicates were shown in the respective figures. Where applicable, quantitative data were presented as mean ± standard deviation (SD).

### Reporting summary

Further information on research design is available in the Nature Portfolio Reporting Summary linked to this article.

## Supplementary information

Supplementary Information

Description of Additional Supplementary Files

Supplementary Video 1

Supplementary Video 2

Reporting Summary

Transparent Peer Review file

## Source data

Source Data

## Data Availability

All other data supporting the findings of this study are available in the main text and the Supplementary Information. The data generated in this study are provided in the Source Data file. Source data are provided with this paper.
