# Supplementary material for: Conformal graphene coatings on ordinary fabrics for wearable electronic devices
Source: Nat Commun. 2026 May 18;17:6565. doi: 10.1038/s41467-026-73319-2 (PMC13381529; doi:10.1038/s41467-026-73319-2)
Supplement: Supplementary file 1 — Supplementary Information [file 41467_2026_73319_MOESM1_ESM.pdf]

Conformal Graphene Coatings on Ordinary Fabrics for Wearable Electronic Devices

Index for Supplementary Information

1. **Microscopic characterization of rGO-PMF** 2
2. **Mechanism of temporal decoupling strategy and its universality** 5
3. **Massive production and applications** 26
4. **Supplementary References** 57

1. Microscopic characterization of rGO-PMF

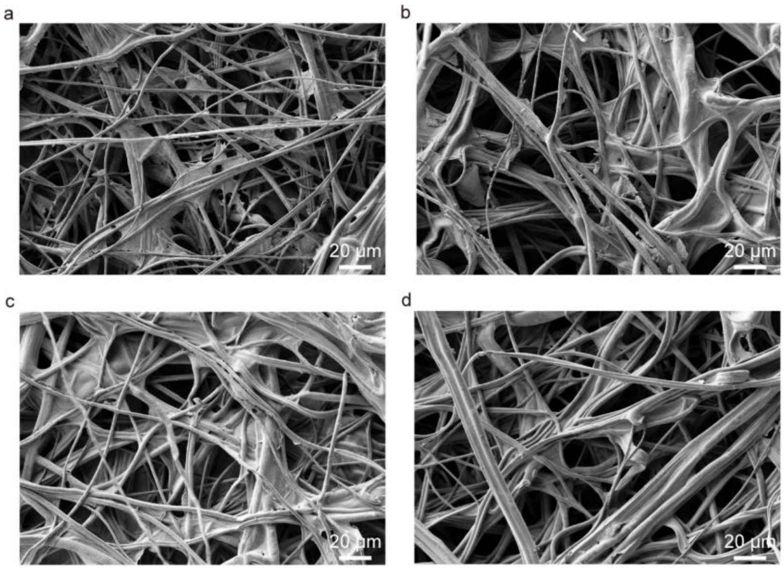

Supplementary Fig. 1 | Scanning electron microscope (SEM) images of rGO-PMF-n. (n=1, 4, 8, 12) a, The SEM image of rGO-PMF-1. **b,** The SEM image of rGO-PMF-4. **c,** The SEM image of rGO-PMF-8. **d,** The SEM image of rGO-PMF-12.

SEM images reveal dynamic microstructural evolution during cycles of dipping (Supplementary Fig. 1). In rGO-PMF-1, rGO nanosheets attach to individual fibers and form initial bridges; however, many isolated, protruding rGO nanosheets are observed, which partially occlude inter-fiber pores without establishing effective conduction pathways. After 4 cycles, the bridging network becomes more extensive, yet persistent isolated rGO structures further lead to filling the interstitial space, resulting in a reduction of pore openness. By rGO-PMF-8, the rGO bridges become continuous and coherent, eliminating most non-contributing nanosheets and beginning to draw adjacent fibers closer. At 12 cycles, this process culminates in the bundling of fibers, which reconfigures the pore architecture by creating larger inter-bundle voids. Further coating to 16 cycles (Fig. 2e) leads to the establishment of rGO bridges between these bundles, forming a hierarchical conductive network while largely preserving the regained structural openness.

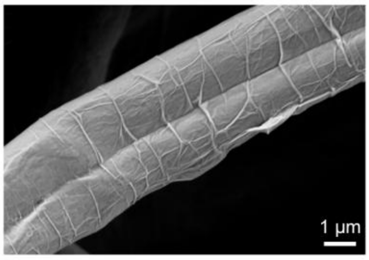

Supplementary Fig. 2 | The SEM image of bundling structure.

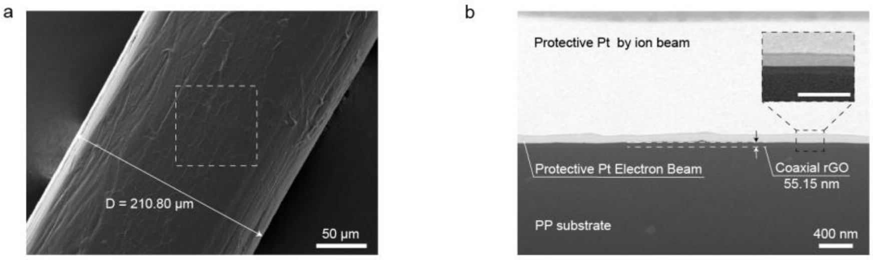

Supplementary Fig. 3 | Characterization of large diameter single fiber's surface, cross-section. **a,** The SEM image of the surface of the rGO coated large diameter fiber. **b,** The SEM image of the cross-section of the rGO coated large diameter fiber.

As shown in Supplementary Fig. 3, we have performed SEM to investigate the conformal coaxial rGO coating of a single PP fiber with a larger diameter (210.80 μm) by the same coating method. Clearly, distinct wrinkles of rGO appear on the surface, showing that the coaxial rGO coating is successfully assembled. Moreover, the cross-section SEM image captured by Cryogenic Focused Ion Beam (Cryo-FIB-SEM) exhibits a highly conformal coaxial rGO coating with a thickness of 55.15 nm. Considering the micro-sized fibers (1–5 μm) typically found in the PMF, the successful construction of a highly conformal coating on this 210.80 μm fiber unambiguously confirms the size universality of our coaxial coating strategy.

2. Mechanism of temporal decoupling strategy and its universality

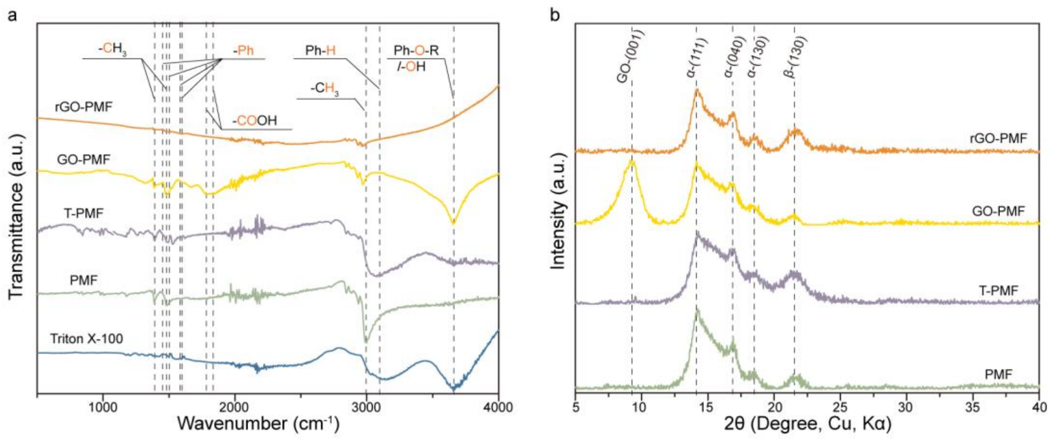

Supplementary Fig. 4 | The Fourier transform infrared spectroscopy (FTIR) and X-ray diffraction patten (XRD) of Triton and multiple PMFs. a, The FTIR of Triton and multiple PMFs. **b,** The XRD of Triton and multiple PMFs.

FTIR reveals the changes of functional groups in Triton and multiple PMFs, which confirms the synthesis mentioned above (Supplementary Fig. 4a). Triton treated PMF is named as T-PMF and GO coated PMF is called GO-PMF. The phenyl group characteristic peak from 1450 cm^{-1} to 1600 cm^{-1} of Triton is absent. It is caused by the symmetrical position of charges since octyl groups have similar electronegativity to polyoxyethylene ether chains, which also confirms the non-ionicity of Triton. The smooth peak appearing on the redshift side at 3000.00 cm^{-1} indicates the coexistence of alkane hydrogen and aromatic hydrogen, while the peak at 3654.44 cm^{-1} indicates the presence of hydroxyl and ether bonds. Compared with Triton, PMF exhibits two methyl characteristic peaks at 1388.98 cm^{-1} and 1483.96 cm^{-1}, while sharp characteristic peaks belonging to alkane hydrogen appear at 3000.00 cm^{-1}. The characteristic peak feature of T-PMF is the combination of PMF and Triton. Three features confirm the successful synthesis of T-PMF, which include the appearance of multiple peaks from 800 cm^{-1} to 1600 cm^{-1}, the blue shift of the co-peak of alkane/aromatic hydrogen composition, and

the smooth peak of hydroxyl and ether bonds at 3646.25 cm^{-1}. The reappearance of the hydroxyl peak at 3654.93 cm^{-1} in GO-PMF confirms the appearance of a coaxial GO layer, while the peaks of carbonyl groups in carboxylic acid groups at 1787.21 cm^{-1} and 1833.50 cm^{-1} also confirm this. The characteristic peaks corresponding to methyl groups reappeared at 1388.98 cm^{-1} and 1483.96 cm^{-1} due to few incomplete coated sites. However, the hydroxyl characteristic peak that forms hydrogen bonds undergoes a blue shift, forming a co peak with alkane hydrogen at 2975.62 cm^{-1}. Finally, we investigated the relatively flat FTIR of rGO-PMF. Compared with GO-PMF, some extremely weakened peaks have attracted attention, namely the carbonyl peaks at 1787.21 cm^{-1} and 1833.50 cm^{-1}, as well as the hydroxyl and ether bond peaks at 3646.25 cm^{-1}. This is a hallmark of the moderate reduction of coaxial GO layer by removing oxygen-containing functional groups through thiourea dioxide. Besides, GO undergoes certain rearrangement in the aqueous phase during reduction, resulting in the loss of methyl peaks at 1388.98 cm^{-1} and 1483.96 cm^{-1} and the hydrogen peak of alkanes becomes weaker at 2975.62 cm^{-1}. This phenomenon is beneficial for coaxial coating.

XRD shows the consistency of phase analysis results and designed synthesis (Supplementary Fig. 4b). Three diffraction peaks of the α crystal of PP can be observed in multiple PMFs, located at $2\theta=14.25°$ (111), $2\theta=16.91°$ (040), and $2\theta=18.68°$ (130), respectively. At $2\theta=21.51°$, another shared diffraction peak of PMFs indicates the presence of β crystal (130) in PP, while the other two characteristic peaks belonging to β crystal are covered by the characteristic peaks of α crystals in PP. Comparing the XRD patterns of PMF and T-PMF, their similarity can be observed. This similarity proves that using EA as a swelling agent, the swelling phenomenon generated when EA enters the PP is reversible, and it will not affect the crystallization of PP itself when EA leaves. Moreover, GO-PMF has a sharp characteristic peak at $2\theta=9.31°$, which is the main feature and strong evidence belonging to GO. However, the position of this characteristic peak is offset towards small angle direction relative to the reported

positions of GO characteristic peaks. This is because the selected GO nanosheets are smaller than the commonly used GO nanosheets, resulting in the offset. After moderate reduction of GO-PMF, the characteristic peak belonging to rGO around $2\theta=10°$ is missing. The reason for this phenomenon is the thin layers of rGO and low degree of chemical reduction of rGO, resulting in low crystallinity, which prevents the characteristic diffraction peaks of rGO from being displayed in XRD pattern.

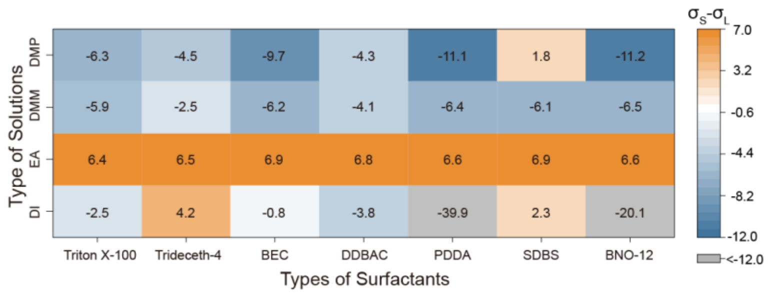

Supplementary Fig. 5 | The Surface free energy D-values of PP and surfactants' solvent solution.

Surfactants	Solutions			
	DI water	Ethyl Acetate (EA)	Dimethyl Malonate (DMM)	Dimethyl Phthalate (DMP)
Pure	71.3	23.2	36.3	41.2
Triton	32.6	23.7	36.0	36.4
Trideceth-4	25.9	23.6	32.6	34.6
Benzethonium Chloride (BC)	30.9	23.2*	36.3*	39.8*
Benzyldimethyldodecylammonium Chloride (DDBAC)	33.9	23.3*	34.2*	34.4*
Poly Dimethyl Diallyl Ammonium Chloride (PDDA)	70.0	23.5*	36.5*	41.2*
Sodium Dodecyl Benzene Sulfonate (SDBS)	27.8	23.2*	36.2*	28.3*
Naphthol polyoxyethylene ether (BNO-12)	50.2	23.5*	36.6	41.3*

Supplementary Table 1 | Surface free energy of different surfactant solutions (mN/m).

The surface free energy of PP is 30.1 mN/m.

* Data are detected from saturated solutions, due to the insufficient solubility of some surfactants in solvents.

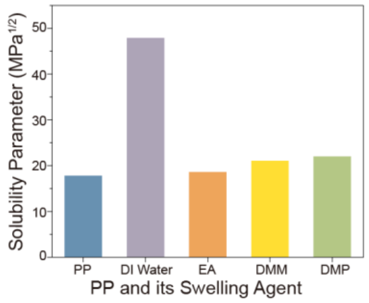

Supplementary Fig. 6 | The solubility parameter of PP and multiple swelling agents.

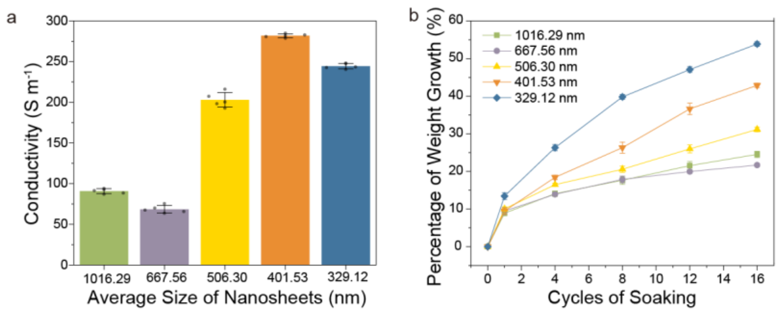

Supplementary Fig. 7 | The conductivity of multiple rGO-PMF-16s in different average sizes of GO nanosheets and their GO weight growth. a, The conductivity of rGO-PMF-16s in different average sizes of GO nanosheets (n=5). **b,** The GO weight growth of rGO-PMF-16s under different average sizes of GO nanosheets in dipping process (n=3). All data in this figure is presented as mean ± SD.

We systematically investigate the conductivity over 1 to 16 dipping cycles to comprehensively capture the evolving architecture of the conductive network, rather than relying on a single data point. Samples synthesized by larger nanosheets should, in principle, exhibit higher conductivity. However, our experimental results (Supplementary Fig. 7a) reveal a non-monotonic trend in conductivity, indicating that macroscopic electrical performance is governed not only by the intrinsic properties of individual nanosheets but also by the architecture of the conductive network within the fabrics. The observed behavior can be interpreted as a trade-off between a penetration-limited regime (large GO) and a junction resistance-limited regime (small GO):

Large GO nanosheets (1016.29 nm and 667.56 nm): These sheets show limited penetration into PMFs, as evidenced by their low GO weight growth during dipping (Supplementary Fig. 7b). They form sandwich structure rather than a continuous 3D coaxial network, resulting in low conductivity despite high intrinsic in-plane conductivity.

Intermediate GO nanosheets (506.30 nm, 401.53 nm): This range represents an optimal balance. The nanosheets penetrate effectively (shown by a sharp increase in

GO weight growth) and coaxially coat the fibers, establishing a robust, long-range conductive network. The peak conductivity at 401.53 nm reflects this optimal regime.

Small GO nanosheets (329.12 nm): While achieving the highest loading and penetration, the conductive network formed by these small sheets contains an excessively high density of inter-nanosheet junctions. This introduces significant electron junction resistance[1,2], which dominates and leads to a decrease in overall conductivity.

In summary, the conductivity of rGO-PMFs is also dominantly controlled by the architecture of the conductive network. The non-monotonic trend results from a transition from a penetration-limited regime (large GO) to a junction resistance-limited regime (small GO), with an optimal intermediate size.

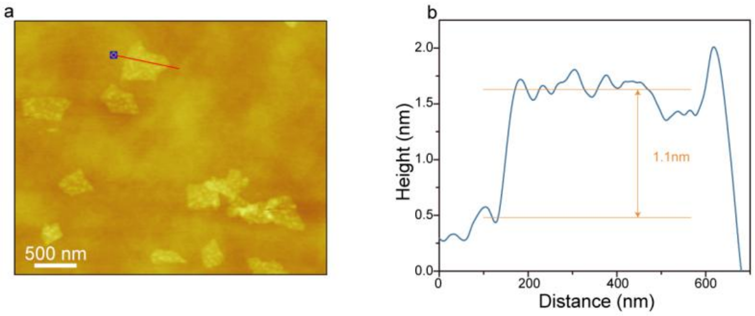

Supplementary Fig. 8 | The atomic force microscopy (AFM) image and its analysis of GO nanosheets at the average size of 401.53nm. a, The AFM image of GO nanosheets at the average size of 401.53 nm. **b,** The height analysis of the scribed nanosheet in supplementary Fig. 7a.

The obtained GO nanosheets have the same thickness due to the same contrast (Supplementary Fig. 8a). And a detailed analysis of the scribed part shows that the GO nanosheet is single layer (Supplementary Fig. 8b).

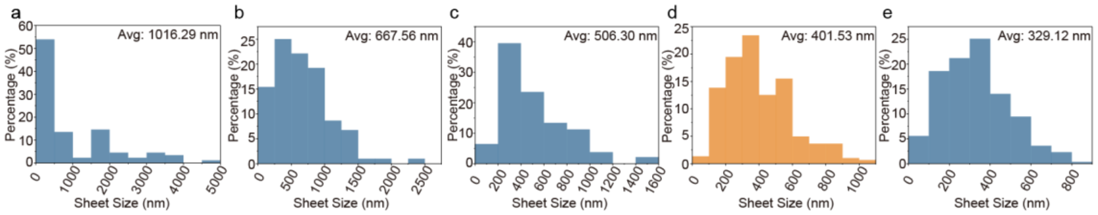

Supplementary Fig. 9 | The size distribution histograms of the GO nanosheets. a, The size distribution histogram of the GO nanosheets at the average size of 1016.29 nm. **b,** The size distribution histogram of the GO nanosheets at the average size of 667.56 nm. **c,** The size distribution histogram of the GO nanosheets at the average size of 506.30 nm. **d,** The size distribution histogram of the GO nanosheets at the average size of 401.53 nm. **e,** The size distribution histogram of the GO nanosheets at the average size of 329.12 nm.

The obtained GO nanosheets show concentrations around their average sizes.

**Surfactants**	**Ionicity**
Triton	Non-Ionic
Trideceth-4	Non-Ionic
BC	Positive
DDBAC	Positive
PDDA	Positive
SDBS	Negative
BNO-12	Non-Ionic

Supplementary Table 2 | Surfactant properties.

Triton Type	Chemical Formula	HLB Value
Triton X-15[3]	*tert-Octyl*-C_6H_4-$(OCH_2CH_2)_{1.5}OH$	4.9
Triton X-45[4]	*tert-Octyl* -C_6H_4-$(OCH_2CH_2)_{4.5}OH$	9.8
**Triton X-100**[5]	*tert-Octyl* -C_6H_4-$(OCH_2CH_2)_{9.5}OH$	**13.4**
Triton X-165[6]	*tert-Octyl* -C_6H_4-$(OCH_2CH_2)_{16}OH$	15.5
Triton X-305[7]	*tert-Octyl* -C_6H_4-$(OCH_2CH_2)_{30}OH$	17.3

Supplementary Table 3 | Different HLB values of Triton series. HLB stands for Hydrophile-Lipophile Balance.

The selection of Triton is based on the Hydrophile-Lipophile Balance (HLB) value, which is a critical parameter for surfactant efficacy in hydrophilic modification. As discussed in prior work[8], an HLB value that is too low results in insufficient hydrophilicity, while a value that is too high compromises the surfactant's affinity for the polypropylene (PP) matrix. The optimal HLB range has been identified as 11.9 to 14.8. Accordingly, we select Triton X-100, which has an HLB value of 13.5, falling squarely within this optimal range, as compared to other Triton variants (Supplementary Table 3).

**Surfactants**	**Aliphatic Properties**	**Polar Properties**	**Aromatic Properties**
Triton	√	√	√
Trideceth-4	√	×	√
BC	√	√	√
DDBAC	√	√	√
PDDA	√	√	√
SDBS	√	×	√
BNO-12	×	√	√

Supplementary Table 4 | Surfactant properties.

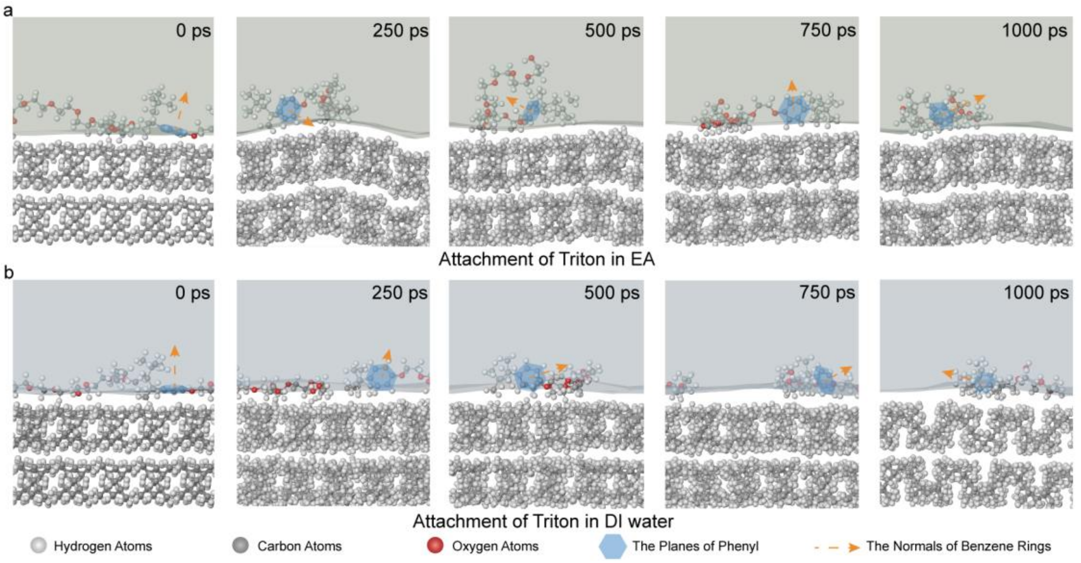

Supplementary Fig. 10 | Schematic molecular dynamics simulation of Triton's attachment in solvents. a, Schematic molecular dynamics simulation of Triton's attachment posture change in EA. **b,** Schematic molecular dynamics simulation of Triton's attachment posture change in DI water.

The molecular dynamics simulation of attachment behavior of Triton in EA and DI water are demonstrated graphically (Supplementary Fig. 10a and 10b). Triton was set on the PP surface at 0ps in EA. At 250 ps, the Triton molecule remains on the surface of PP, but its polyoxyethylene ether chain spontaneously self-forms a ring secondary structure, which is caused by the hydrogen bonding between ether bonds and the hydroxyl groups (Supplementary Fig. 10a). It leads to the disappearance of the σ-π conjugation of phenyl group and PP, and further results in the orthogonalization of the plane of the phenyl group and the plane of the PP surface. Later, at 500 ps, the self-forming secondary structure is disrupted by strong σ-σ conjugation and hydrogen bonding between Triton and EA, but Triton molecules still absorb on the surface of PP. At 750 ps, the self-forming ring reappeared, while the σ-σ conjugation between the octyl group and polyoxyethylene ether chains appeared at 1000 ps, resulting in a larger self-forming ring. It is worth noting that the phenyl group within the Triton molecule maintains a large dihedral angle with the PP surface throughout 1000 ps. The posture change of Triton in DI water within 1000 ps is very similar to that in EA (Supplementary

Fig. 10b). Triton stably attaches to the surface of PP, and its phenyl group plane always maintains a large dihedral angle with the PP surface. The self-forming secondary structure of polyoxyethylene ether chains is also exhibited in the aqueous phase, but the current solvent is DI water, resulting in the absence of a macrocyclic structure composed of octyl group and polyoxyethylene ether chains. The special posture of the large dihedral angle between the plane where the phenyl group is located and the surface of PP, especially when the phenyl group stands upright on the surface of PP, makes it easier for the phenyl group structure of Triton to expose the positively charged carbon atom layer without being masked by the conjugated π electron cloud on the upper and lower sides. Furthermore, it is easy to form side to side π-π stacking (T-type π-π stacking) with rGO. T-type π-π stacking can also enhance durability of coaxial coating, functioning with offset face-to-face stacking (F-type π-π stacking)[9–11].

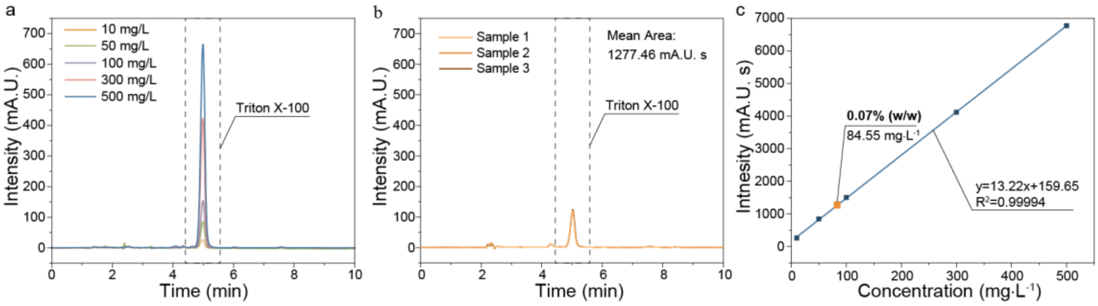

Supplementary Fig. 11 | Triton Concentration of rGO-PMF-16 detected by HPLC. a, The HPLC peaks of Triton at different concentrations. **b,** The HPLC results of Triton in rGO-PMF-16. **c,** The Triton concentration of samples on the linear fit standard curve.

We have conducted a comprehensive review of laws and regulations in most major industrial countries. Notably, the European Union (EU) stands out as the only region with specific restrictions on Triton X-100 (Triton) and its derivatives. Importantly, a key exemption under this regulation specifies that authorization is not required if the substance concentration in the final products remains below 0.1% (w/w)[12,13].

To definitively verify the compliance of rGO-PMFs-16, quantitative analysis is conducted by high-performance liquid chromatography (HPLC). A standard calibration curve with excellent linearity (R^2 = 0.99994) is established through series of Triton solutions Supplementary Fig. 10a and c). And Triton is extracted from fabric samples (n=3) via Soxhlet extraction (Supplementary Fig. 10b). The results are highly consistent, yielding a calculated concentration of 0.07% (w/w) of Triton in rGO-PMFs-16. This value is conclusively below the 0.1% regulatory threshold, confirming that the production and sale of rGO-PMFs-16 are fully compliant with current EU REACH regulations.

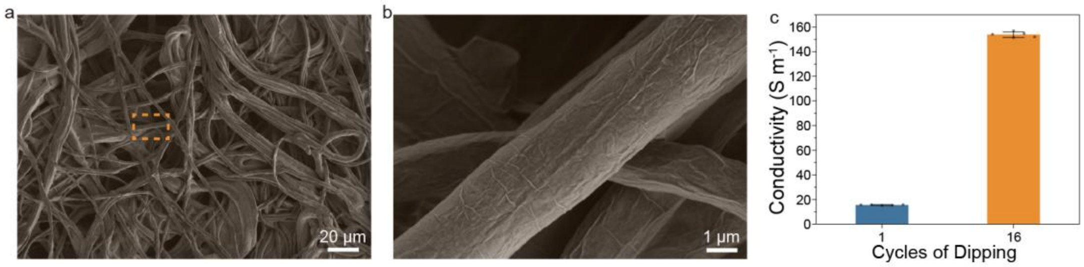

Supplementary Fig. 12 | The synthesis results of rGO-cPMFs. a, The SEM image of rGO-cPMF-16's surface. **b,** The SEM image of marked part in figure R2a. **c,** The conductivity of rGO-cPMF-1 and rGO-cPMF-16 (n=5). All data in this figure is presented as mean ± SD.

We value underlying concern regarding the ecological footprint of manufacturing. The significance of TD strategy lies not in the specific surfactant used, but in the universal TD strategy. To proactively demonstrate this versatility and address environmental considerations, a synthesis is successfully replicated using cardanol polyoxyethylene ether (CPE-9), a surfactant with a superior environmental profile[14–16]. The resulting fabrics (rGO-cPMF-x) exhibit a conformal rGO coaxial coating (Supplementary Fig. 12a and b) and achieve high conductivity (up to 153.87 S m^{-1}, Supplementary Fig. 12c). These results unequivocally prove that TD Strategy is universally adaptable and provides a direct pathway for developing high-performance conductive fabrics with a significantly reduced ecological impact.

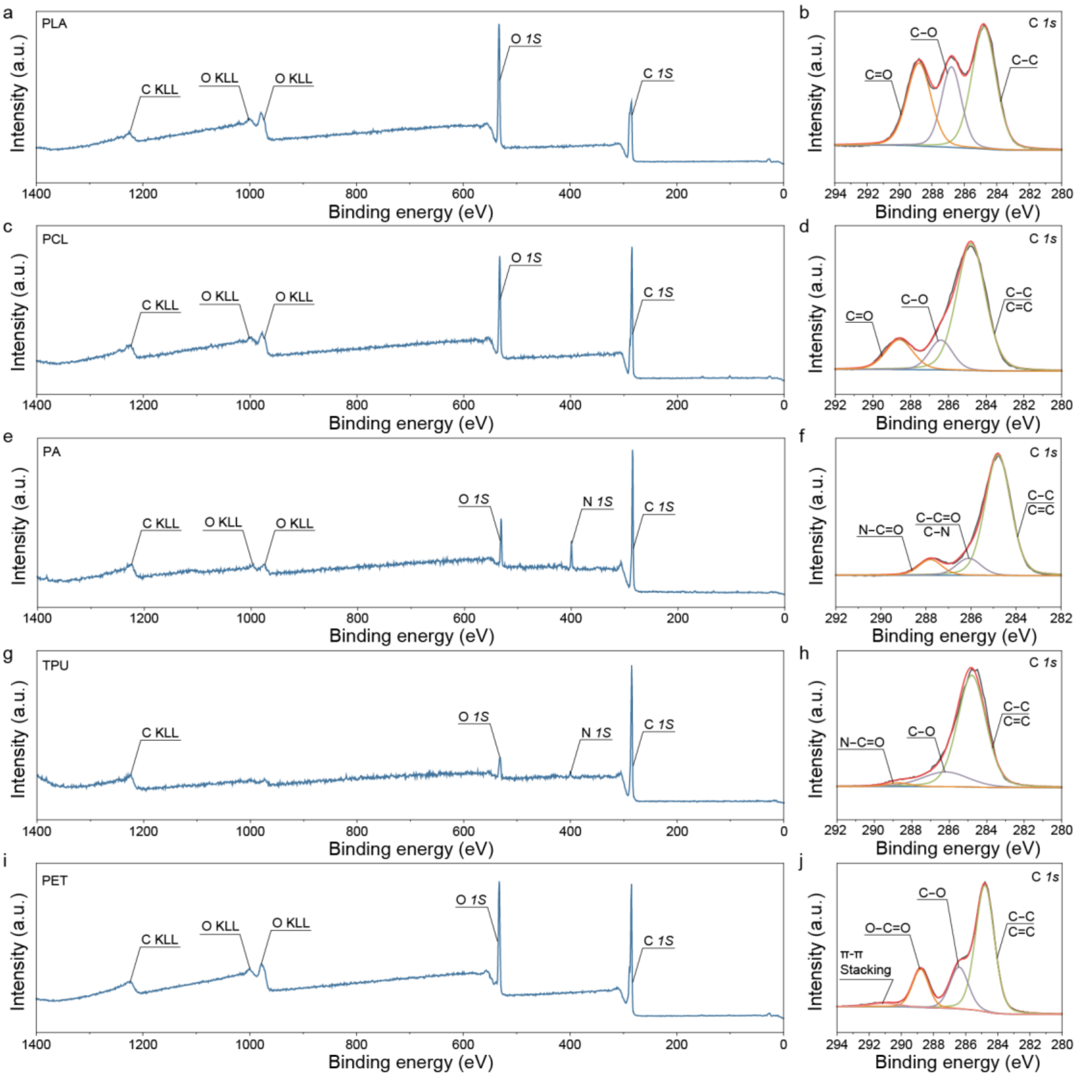

Supplementary Fig. 13 | XPS spectra of the untreated commercial fabrics. a-b, Full spectrum and high-resolution C 1s spectrum of PLA**. c-d,** Full spectrum and high-resolution C 1s spectrum of PCL**. e-f,** Full spectrum and high-resolution C 1s spectrum of PA**. g-h,** Full spectrum and high-resolution C 1s spectrum of TPU**. i-j,** Full spectrum and high-resolution C 1s spectrum of PET.

We have conducted systematic XPS characterization on the as-received commercial polymer/natural fabrics. The results reveal no detectable evidence of pre-existing surface treatments across multiple common fabric types (Supplementary Fig. 13).

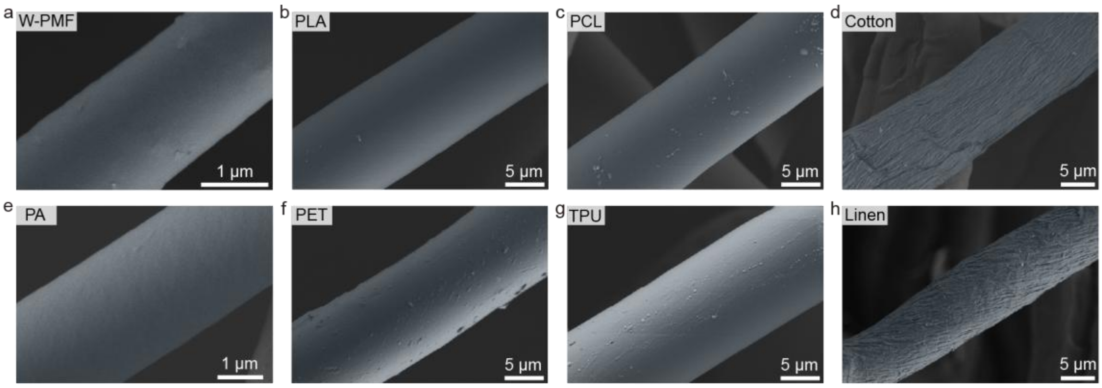

Supplementary Fig. 14 | The SEM images of W-PMF, PLA, PCL, PA, PET, TPU, PET, Cotton and Linen.

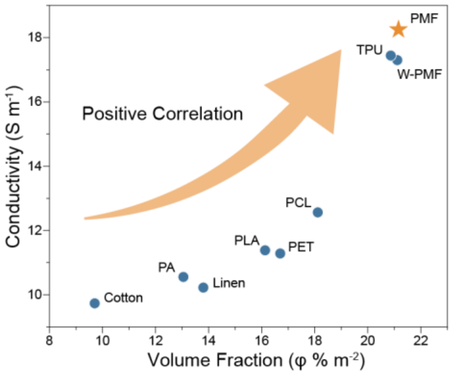

Supplementary Fig. 15 | The conductivity and volume fraction (φ) of each commercial polymer/natural fabrics.

Polymer/ Natural Fabrics	Conductivity (S m^{-1})	Intrinsic Density (g cm^{-3})	Weight (g m^{-2})	Thickness (μm)	Volume Fraction (φ % m^{-2})
PMF	18.26	0.91	25.03	130.01	21.16
W-PMF	17.30	0.91	24.99	130.06	21.11
PCL	12.56	1.15	25.00	120.00	18.12
PLA	11.38	1.24	30.00	149.97	16.13
PA	10.55	1.15	30.03	200.07	13.05
PET	11.28	1.40	35.07	150.00	16.70
TPU	17.44	1.20	50.09	199.98	20.87
Cotton	9.73	1.54	50.10	335.04	9.71
Linen	10.22	1.55	59.92	280.09	13.80

Supplementary Table 5 | Conductivity, intrinsic density, weight, thickness and volume occupation of each commercial polymer/natural fabrics. The fiber network volume fraction (φ) is calculated as: φ = (Weight / Intrinsic Density) / Thickness. This represents the volume fraction of solid polymer/natural fibers within the total volume of the fabric, providing a quantitative measure of the network's denseness and available scaffold for rGO coating.

While rGO is indeed the conductive component, our results demonstrate that the microstructure of the fabric scaffold is a critical determinant for the efficiency of electron transport. We quantify this microstructural influence by introducing the fiber network volume fraction (φ), which represents the volumetric density of the fiber network (Supplementary Table 5). Our analysis reveals a strong positive correlation between φ and conductivity (Supplementary Fig. 15). A higher φ signifies a denser, more interconnected scaffold, which facilitates the formation of a continuous, long-range coaxial rGO conductive pathway. Consequently, while the intrinsic properties of rGO set the fundamental conductivity ceiling, the solid fraction of the underlying polymer/natural fabric is also a key modulating factor for the realized conductivity in the rGO-PMFs. And the construction of an efficient conductive rGO network is fundamentally governed by the solid fraction of the underlying polymer/natural fabric scaffold.

3. Massive production and applications

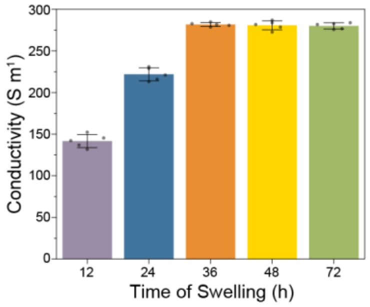

Supplementary Fig. 16 | The conductivity of rGO-PMF-16 under different swelling time (n=5). All data in this figure is presented as mean ± SD.

The influence of swelling time is investigated. As shown in Supplementary Fig. 16, the conductivity of rGO-PMF-16 reaches a plateau after 36 h, indicating that a swelling time of 36 h is sufficient for the process. Therefore, 36 h is selected as the optimal swelling time for all subsequent experiments.

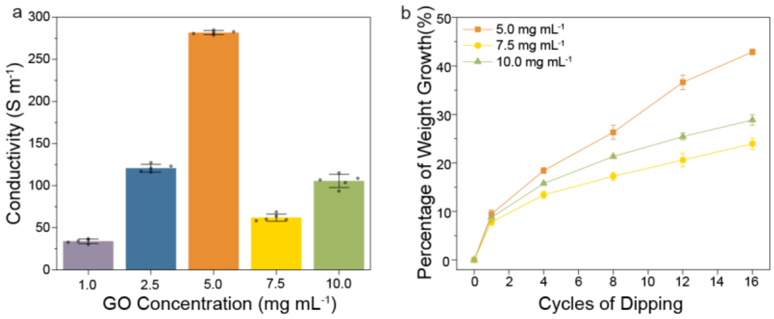

Supplementary Fig. 17 | The conductivity of rGO-PMF-16 under different GO concentration and the weight growth of rGO-PMF-16s under 5.0, 7.5 and 10 mg mL^{-1}. a, The conductivity of rGO-PMF-16 under different GO concentrations (n=5)**. b,** The GO weight growth of rGO-PMF-16 under 5.0, 7.5 and 10 mg mL^{-1} in dipping process (n=3)**.** All data in this figure is presented as mean ± SD.

As shown in Supplementary Fig. 17a, the conductivity of rGO-PMF-16 depends strongly on GO concentration, reaching a maximum at 5.0 mg mL^{-1}. This optimum corresponds to an efficient coaxial coating structure, where GO nanosheets uniformly penetrate the fabrics, forming a continuous 3D conductive pathway. At higher concentrations (7.5 and 10 mg mL^{-1}), however, excessive GO limits deep penetration, resulting in lower weight growth (Supplementary Fig. 17b) and inefficient conductive networks. Consequently, both higher concentrations yield inferior conductivity compared to the optimal 5.0 mg mL^{-1} condition with its fully interconnected conformal 3D network.

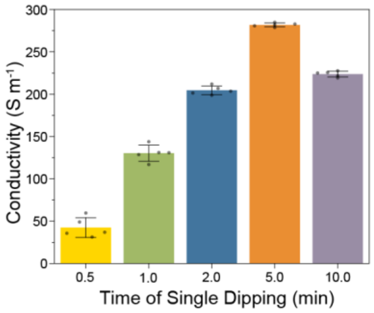

Supplementary Fig. 18 | The conductivity of rGO-PMF-16 under different single dipping time (n=5). All data in this figure is presented as mean ± SD.

The effect of a single dipping cycle is investigated. As presented in Supplementary Fig. 18, rGO-PMF-16 prepared with a 5.0 min dipping time exhibits the highest conductivity. A shorter time may lead to insufficient adsorption, while a longer time might cause nanosheet re-stacking. Therefore, a 5.0 min single dipping time is identified as optimal.

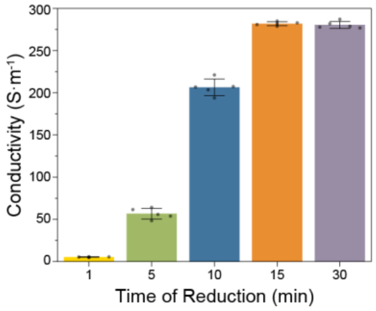

Supplementary Fig. 19 | The conductivity of rGO-PMF-16 under different reduction time(n=5). All data in this figure is presented as mean ± SD.

The effect of chemical reduction time is investigated. As shown in Supplementary Fig. 19, the conductivity of rGO-PMF-16 plateaus after 15 min of reduction, indicating that 15 min is sufficient for effective reduction. Consequently, a reduction time of 15 min is adopted for all samples.

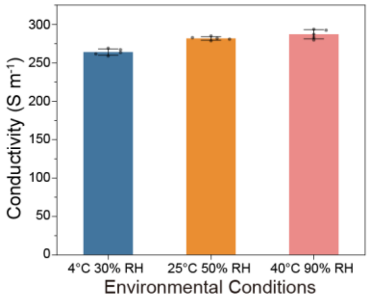

Supplementary Fig. 20 | The Conductivity of rGO-PMF-16 in different conditions (n=5). All data in this figure is presented as mean ± SD.

We have conducted additional experiments to evaluate the synthesis under different humidity and temperature conditions, simulating real-world scenarios: Winter-like condition: 4 °C and 30% RH and Summer-like condition: 40 °C and 90% RH.

The results are summarized in Supplementary Fig. 20. We observe that the conductivity under low-temperature conditions (4 °C, 264.03 S m^{-1}) is lower than that at room temperature (25 °C, 281.78 S m^{-1}), primarily due to slowed swelling and surface functionalization. In contrast, the conductivity under high-temperature conditions (40 °C, 287.13 S m^{-1}) is only 1.90% higher than that at room temperature, indicating that the swelling process is sufficient to achieve optimal functionalization.

Notably, relative humidity showed negligible influence on conductivity, as the process is based on a wet-chemical route involving sequential soaking in EA and DI-water based solutions. Although higher temperatures slightly enhance swelling kinetics, they also increase energy consumption and safety risks due to the use of EA. Therefore, we recommend room temperature (25 °C, 50% RH) as the optimal condition for balancing performance, cost, and safety.

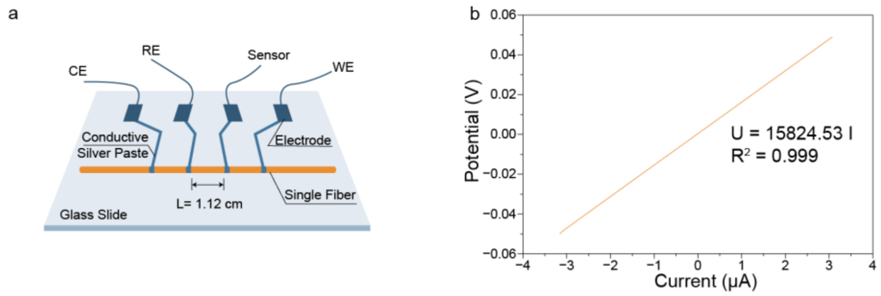

Supplementary Fig. 21 | Characterization of single fiber conductivity. c, Schematic diagram of the revised four probe conductivity measurement setup (CE represents Counter Electrode; RE represents Reference Electrode; Sensor represents Sensing Electrode, and WE represents Working Electrode). **d,** U-I curves from the LSV results.

To measure the conductivity of the single fiber, linear sweep voltammetry (LSV) was conducted using a customized four-probe setup to effectively eliminate contact resistance (Supplementary Fig. 21a). As shown in the highly linear U-I curve (Supplementary Fig. 21b), the measured resistance (R) of the large-diameter fiber (D = 210.80 μm, Supplementary Fig. S3) is 15,824.53 Ω between RE and Sensor. The apparent conductivity (σ_{app}) of large-diameter fiber is 20.28 S m^{-1}, calculated by the following formula:

$$\sigma_{app} = \frac{L}{R \cdot A_{total}} \tag{2}$$

Where:

σ_{app} is the conductivity in the z-direction;

L is the length between RE and Sensor;

R is the resistance of the large-diameter fiber between RE and Sensor;

A_{total} is the the total cross-sectional area.

Since the massive PP core is electrically insulating, we extracted the true intrinsic conductivity (σ_{int}) of the 55.15 nm rGO shell (Supplementary Fig. S3) utilizing its effective cross-sectional area (A_{rGO}) by the following formula:

$$\sigma_{int} = \frac{L}{R \cdot A_{rGO}} \tag{3}$$

Where:

σ_{int} is the intrinsic conductivity of the conformal rGO shell;

A_{rGO} is the effective cross-sectional area of the rGO shell.

This yields an intrinsic conductivity of 19,378.55 S m^{-1}, resulting from our moderate chemical reduction. In PMF, the PP fibers possess much smaller diameters (D) ranging from 1 to 5 μm. By applying this intrinsic conductivity and the most conservative rGO thickness (t = 25 nm, Figure 2l) to these microfibers, their theoretical apparent conductivity (σ_{micro}) is calculated by the following formula:

$$\sigma_{micro} = \sigma_{int} \frac{4t}{D} \quad (4)$$

Where:

σ_{int} is the theoretical apparent conductivity of the microfiber in rGO-PMF-16;

t is the most conservative rGO coating thickness on the microfiber (25 nm);

D is the diameter of the microfiber in PMF (1–5 μm).

Through this scaling effect, the theoretical apparent conductivity of the fibers in rGO-PMF-16 achieves a lower bound of 387.57 (5 μm) to 1,937.86 S m^{-1} (1 μm). In traditional porous materials, macroscopic conductivity typically drops by 1 to 2 orders of magnitude due to massive contact resistance at fiber intersections. Remarkably, the macroscopic in-plane conductivity of rGO-PMF-16 reaches 283.06 S m^{-1}, demonstrating an ultra-high retention rate. This strongly supports that our observed rGO bridges and fiber bundles (Supplementary Fig. 1) act as effective welds, minimizing point-contact resistance and translating superior single-fiber performance into a highly efficient 3D conductive network.

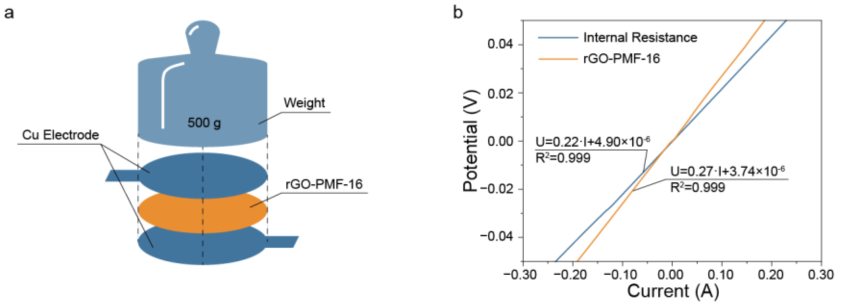

Supplementary Fig. 22 | Characterization of rGO-PMF-16's z-direction conductivity. a, Schematic diagram of the z-direction conductivity measurement setup. The sample is clamped between two Cu electrodes under a 500 g weight (~15.6 kPa). **b,** U-I curves from the LSV results.

The conductivity derived from standard 4-probe measurements primarily characterizes the efficient in-plane transport along the continuous fiber network. To explicitly quantify the vertical transport and validate the existence of the 3D conductive pathway, we perform z-direction conductivity measurements using a customized setup in LSV mode of an electrochemical workstation (Supplementary Fig. 22a). This specific pressure is deliberately applied to ensure reliable ohmic contact while strictly preserving the fabric's intrinsic highly porous architecture. Crucially, this value falls well within the real operational pressure of wearable electronics on human skin, distinct from the destructive high-pressure testing (typically >1 MPa) conventionally used for rigid industrial materials like carbon paper. The internal resistance is baseline-corrected (Supplementary Fig. 22b). The net z-direction resistance (R_Z) of the rGO-PMF-16 was 0.05 Ω. Based on the actual thickness and the contact area of the sample the z-direction conductivity (σ_Z) is 8.28 S m^{-1} which is calculated by the following formula:

$$\sigma_Z = \frac{h}{R_Z \cdot A} \tag{5}$$

σ_Z is the conductivity in the z-direction;

h is the actual thickness of the rGO-PMF-16 under the applied load;

R_Z is the net z-direction resistance of the rGO-PMF-16 (0.05 Ω);

A is the contact area of the Cu electrodes (3.14 cm^2).

This result reveals a significant but expected anisotropy compared to the in-plane conductivity (283.06 S m^{-1}). This behavior, caused by non-woven architecture (characterized by randomly oriented fibers stacked in the planar direction rather than an ordered interlaced structure), is consistent with the intrinsic physics of fibrous assemblies. Similar to carbon paper and carbon cloth[17,18], electron transport is naturally more efficient along the continuous fiber axis (in-plane) than hopping across fiber-to-fiber junctions in the thickness direction (z-direction). Crucially, the existence of a measurable z-direction conductivity strongly supports our claim of rGO bridges and fiber bundle structures. In the absence of a conformal coating penetrating the entire fabric depth, the z-direction would show ultra-low conductivity or even remain electrically insulated. The ability to conduct current through the thickness validates the formation of a fully interconnected 3D conductive network, despite the anisotropic efficiency.

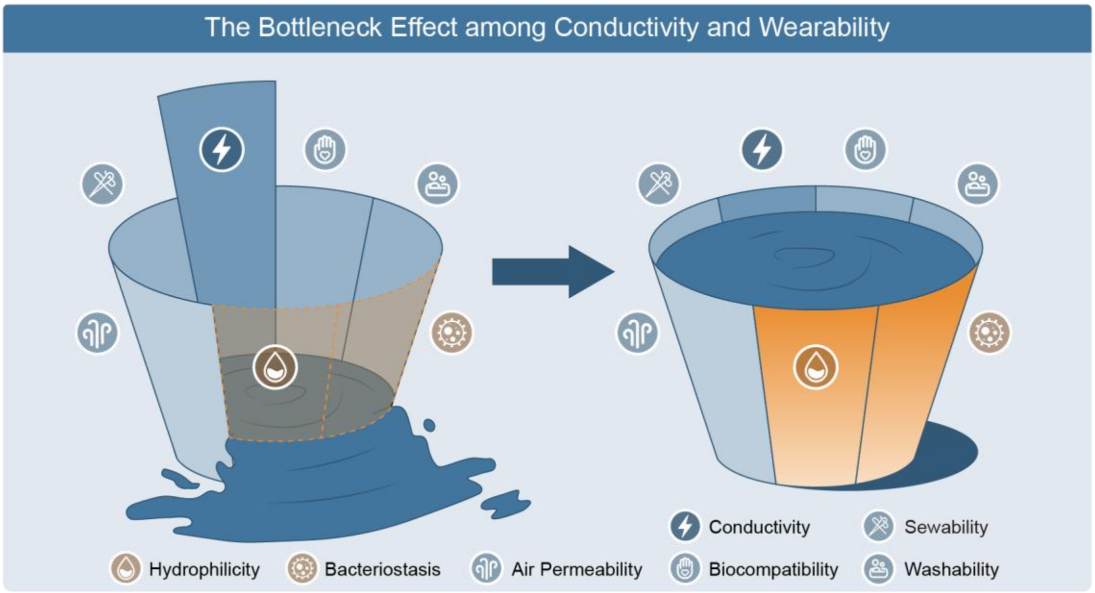

Supplementary Fig. 23 | The bottleneck effect of conductivity and multiple wearability performance.

Reference	Conductivity (S m^{-1})	Sheet Resistance (Ω sq^{-1})	Fabrics/ Fibers	Wearability*
**Our work**	**283.06**	**27.19**	**PP**	√
**Our work****	**1214.84**	**6.58**	**PP**	×
19	11	-	PP	×
20	35.6	-	PP	×
21	6.24	-	PP	×
22	-	600	Nylon	×
23	10	-	Glass	×
24	3.3	-	Polyamide	×
25	33.6	-	PEDOT:PSS	×
26	57.9	-	Silk	×
27	3.4	-	Cotton	×

Reference	Conductivity ($S\ m^{-1}$)	Sheet Resistance ($\Omega\ sq^{-1}$)	Fabrics/ Fibers	Wearability*
[28]	3.06×10^{-2}	-	Silk	×
[29]	-	3.69×10^{4}	Cotton	×
[30]	450	-	Nylon	×
[31]	1040	-	Nylon	×
[32]	-	2.14×10^{3}	Cotton	×
[33]	0.09	4.5×10^{4}	Wool	×
[34]	10.9	910	Cotton	×
[35]	-	560	Cotton	×
[36]	241.70	11.92	Poly-Cotton	×
[37]	12.9	110	Cotton	×
[38]	0.53	1.1×10^{4}	PES	×
[39]	-	840	Cotton	×
[40]	0.01	-	Acrylic	×
[41]	1300	-	Kevlar	×
[42]	55	113.64	Cotton	×
[43]	146.8	17.03	Aramid	×

Supplementary Table 6 | Conductivity and wearability comparison. *Wearability is concluded based on the reported properties in the respective publications. The absence of reported data for any of the 5 key properties (hydrophilicity, air-permeability, washability, bacteriostasis, and biocompatibility) is considered as a lack of demonstrated wearability. **The data is measured by rGO-PMFs-16_{HI}.

Notably, while conductivity is a critical parameter for the practical applications of wearable electronic fabrics, it is by no means the sole determinant of performance. These materials must also meet essential wearability requirements, including

hydrophilicity, air permeability, washability, bacteriostasis, and biocompatibility[44–46]. In practice, achieving ultra-high conductivity (typically >1000 S m^{-1}) often necessitates aggressive chemical or thermal reduction of graphene oxide (GO), which markedly deteriorates other key attributes vital for wearability—such as hydrophilicity and bacteriostatic properties. As evidenced by peers' works and rGO-PMF-16_{HI} sample (1214.84 S m^{-1}, achieved by HI reduction in this work), such high conductivity often comes at the expense of a holistic wearable profile. This trade-off represents a typical bottleneck effect in the field, as illustrated in Supplementary Fig. 23 (detailed discussion in Supplementary Fig. 26, 29-35). Therefore, rGO-PMF-16 is engineered to achieve an optimal balance, providing substantial conductivity (283.06 S m^{-1}) while retaining hydrophilicity, air permeability, washability, bacteriostasis, and biocompatibility. This balance is crucial because further increasing conductivity yields diminishing returns while severely compromising other wearable properties (detailed discussion in Supplementary Fig. 35). As summarized in Supplementary Table 6, to the best of our knowledge, almost no previous work has achieved a comparable integration of comprehensive wearability features with similarly high conductivity.

Furthermore, TD strategy, which avoids harsh processes meanwhile acquires decent conductivity, is not only gentle on ordinary fabrics but also inherently scalable and practical for mass production, addressing a key challenge in transitioning E-fabrics from the lab to real-world applications.

Category	Item/Process	Price (US$)	Usage (m^{-2})	Cost ($US\$\cdot m^{-2}$)
Chemical Cost	PMF	$1\cdot kg^{-1}$	25 g	2.50×10^{-2}
	GO	$12.14\cdot kg^{-1}$	10.30 g	12.50×10^{-2}
	Triton	$6.86\cdot kg^{-1}$	0.93 g	0.64×10^{-2}
	Thiourea Dioxide	$0.86\cdot kg^{-1}$	6.14 g	0.53×10^{-2}
	Sodium Hydroxide	$0.65\cdot kg^{-1}$	2.70 g	0.18×10^{-2}
	DI water	$0.17\cdot kg^{-1}$	30 g	0.51×10^{-2}
	EA	$0.86\cdot kg^{-1}$	60 g	5.16×10^{-2}
Energy Cost	Preparation	$0.11\cdot kWh^{-1}$	0.51 kWh	5.61×10^{-2}
	GO Coating	$0.11\cdot kWh^{-1}$	0.38 kWh	4.18×10^{-2}
	Reduction	$0.11\cdot kWh^{-1}$	0.43 kWh	4.73×10^{-2}
Carbon Cost	Carbon Emission	$13.28\cdot kg^{-1}$	0.78 kg	1.04×10^{-2}
Total Cost	-	-	-	0.38

Supplementary Table 7 | Production cost of rGO-PMF-16.

Energy Cost (US$ m^{-2}): This is calculated by multiplying the total energy usage for each process ($kWh\cdot m^{-2}$) from Supplementary Table 8 by the local electricity price (US$ 0.11 per kWh), as shown in the "Energy Cost" section of Supplementary Table 7.

Carbon Cost ($US\$\cdot m^{-2}$): This is calculated by first determining the total CO_2 emissions (kg $CO_2\cdot m^{-2}$) from Supplementary Table 8 and then multiplying this value by the applied carbon price (US$ 13.28 per kg of CO_2), as shown in the "Carbon Cost" row of Supplementary Table 7.

All the prices are collected in Chinese domestic market and transformed into US Dollar with the rate: Chinese Yuan: US Dollar = 1:7.

Process	Energy Usage (m^{-2})	Average Emission CO_2 (kWh^{-1})	Production Emission (kg $CO_2 \cdot m^{-2}$)
Preparation	0.51 kWh	594.2 g	0.30
GO Coating	0.38 kWh	594.2 g	0.23
Reduction	0.43 kWh	594.2 g	0.26
Total	1.32 kWh	-	0.78

Supplementary Table 8 | Production cost of rGO-PMF-16.

China's average CO_2 emission is 594.2 g kWh^{-1}.

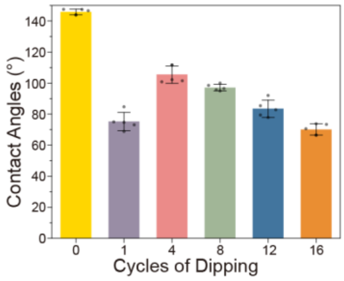

Supplementary Fig. 24 | The contact angles of multiple rGO-PMFs (n=5). All data in this figure is presented as mean ± SD.

According to the data in Supplementary Fig. 24, rGO-PMF-16 obtains the lowest contact angles among all the samples. Moreover, as highlighted in recent studies published for wearable devices interfaced with the skin[47,48], hydrophilicity is crucial for ensuring long-term wear comfort and skin health, and are themselves important dimensions of wearability evaluation. Our material design adheres to this principle, aiming to maintain the integrity and health of the skin barrier.

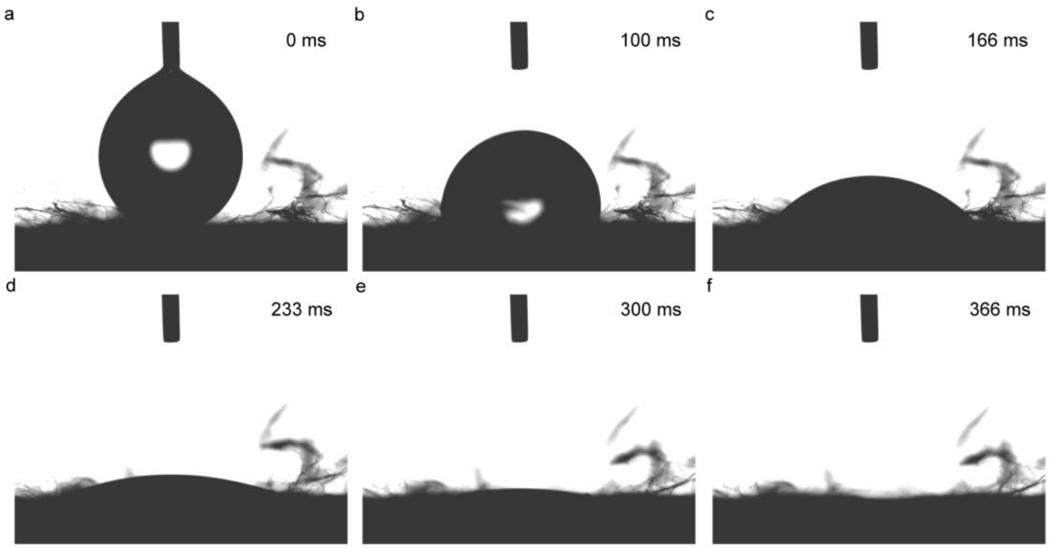

Supplementary Fig. 25 | The optical photos of a water drop on T-PMF surface in 366 ms.

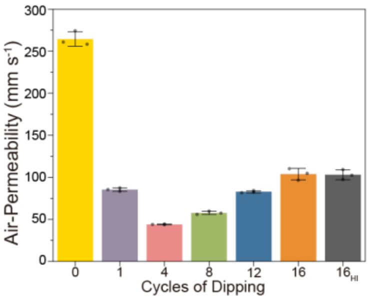

Supplementary Fig. 26 | The air-permeability of multiple rGO-PMFs (n=3). All data in this figure is presented as mean ± SD.

While a straightforward inverse correlation might suggest that the highest conductivity should coincide with the lowest permeability, the microstructure evolution in our coaxial fiber system reveals a more nuanced, three-stage process that fully explains this phenomenon, as evidenced by SEM image analysis (Supplementary Fig. 1, and Figure 2e).

Stage 1 (1-4 cycles): Gap Coverage and Initial Bridging

During the initial cycles, rGO nanosheets coat individual fibers and begin forming bridges at fiber intersections. This progressively reduces inter-fiber gaps, leading to continuously decreasing air permeability, which reaches a minimum at 4 cycles.

Stage 2 (4-12 cycles): Fiber Bundling and Gap Re-opening

With further coating (up to 12 cycles), the rGO structures become substantial enough to pull adjacent fibers into bundled assemblies. This bundling consolidates the conductive material at junctions and, importantly, re-opens larger gaps between bundles, causing air permeability to increase from its minimum.

Stage 3 (12-16 cycles): Inter-Bundle Bridging for Optimal Conductivity

At 16 cycles, substantial rGO bridges connect adjacent bundles, establishing a highly conductive network. Although these inter-bundle structures partially occupy some reopened gaps, they form in a spatially discrete manner that preserves significant inter-bundle porosity. This architecture enables maximal electron transport along the

bridging pathways while maintaining the highest air permeability. Further dipping would lead to excessive inter-bundle filling, compromising air permeability. Thus, rGO-PMF-16 achieves an optimal trade-off, leveraging bridging for conductivity without sacrificing structural openness. Besides, two kinds of reduction express similarity in air permeability as well.

Unlike conventional sandwich-type conductive composites, where conductivity improves through progressive pore filling and air permeability decreases monotonically, our coaxial system relies on bridging rather than void-filling for electron transport. This unique bundled-and-bridged architecture thereby breaks the conventional trade-off between conductivity and air permeability. It achieves this by decoupling the governing pathways: robust inter-bundle rGO bridges establish highly efficient electron transport for maximum conductivity, while the concurrently preserved inter-bundle porosity maintains high air permeability.

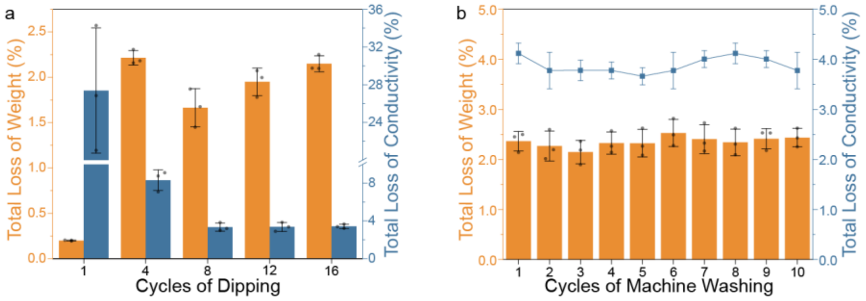

Supplementary Fig. 27 | The deterioration of conductivity and loss of weight for multiple rGO-PMFs in simulated machine washing. a, The results of multiple rGO-PMFs in the first cycle of simulated machine washing (n=3). **b,** The results of multiple rGO-PMF-16 in 10 cycles of simulated machine washing (n=3). All data in this figure is presented as mean ± SD.

Washability is essential for wearable devices. We have directly valued this issue by monitoring the total weight loss and total conductivity loss of the rGO-PMF during machine washing. These two losses diminish gradually as the number of dipping cycles increases (Supplementary Fig. 27a), which supports the effectiveness of our TD strategy. rGO-PMF-16 sample exhibits excellent robustness, with only ~2.3% total weight loss and ~4% total conductivity loss after the first washing cycle. Importantly, these values plateau over the next nine washing cycles, showing no further notable reduction (Supplementary Fig. 27b). This minimal and self-limiting loss indicates that rGO-PMF-16 has decent washability for wearable applications.

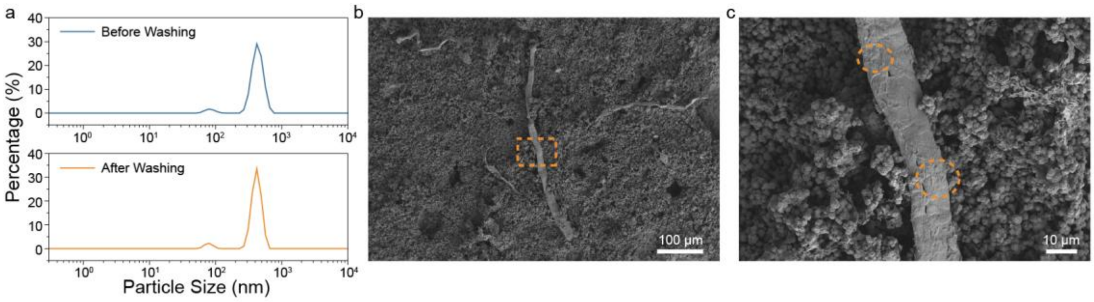

Supplementary Fig. 28 | Characterization of rGO-PMF-16 properties. a, The volume percentage-particle size curves of the washing supernatant before and after washing. **b,** SEM images of the filter material on a 0.22 micrometer filter membrane collected from the washing processes. **c,** The zoom-in of the marked part in b.

Parameter	Value	Reference
Fabrics Weight	32.75 g m^{-2}	Actual Measured
Garment Area	1.0 m^2	Commercial Average
Total Weight Loss	2.3%	Actual Measured
Assumed Release	753 mg $item^{-1}$	Calculated
Daily Sewage Volume	200 L day^{-1}	[49]
Market Penetration	1%	[50]
WWTP Removal	95%	[51]
River Dilution	10	[49]
Safety Threshold	0.63 ppb	[52]
Calculated PEC	0.19 ppb	Calculated

Supplementary Table 9 | Worst-case parameters and calculation of PEC.

However, gravimetric analysis alone is insufficient to detect trace amounts of nano-pollutants. Therefore, we rigorously addressed this concern by implementing a multi-

dimensional assessment strategy that combines spectroscopic analysis (Dynamic Light Scattering, DLS), morphological characterization (Scanning Electron Microscopy, SEM), and a worst-case theoretical exposure model (Predicted Environmental Concentration, PEC).

1. Experimental Verification: Absence of Suspended Nanoparticles

To specifically detect potentially released nanoparticles that a scale would miss, we analyzed the washing supernatant using DLS and SEM. As presented (Supplementary Fig. 28a), the volume-weighted particle size distribution of the washing solution is spectroscopically identical to that of the pure detergent background. Both samples exhibit dominant peak at ~80 and ~420 nm, corresponding to insoluble fillers (e.g., zeolites) inherent to the detergent matrix, with no additional signals detected in the nano-range. This confirms that the rGO coating (Average size = 401.53 nm) did not release detectable suspended nanosheets. Corroborating this, SEM analysis of the filtration residue (Supplementary Fig. 28b) identifies the physical source of the mass loss (~2.3%) as micron-sized fibers detached during mechanical agitation. High-magnification imaging (Supplementary Fig. 28c) reveals that the rGO coating remains firmly anchored to these microfibers rather than peeled off. Thus, the weight loss represents rapid-settling macro-particulates rather than persistent nano-pollutants.

2. Theoretical Safety Net: Worst-Case PEC Calculation (Per Garment)

To quantitatively bound the environmental risk, we performed a PEC calculation following ECHA Guidance[49], modeling the daily washing of a single functional T-shirt (assumed area: 1 m^2). We adopted an extreme "worst-case" assumption where the entire gravimetric mass loss (2.3%) is treated as 100% pure rGO, intentionally disregarding the fiber content. Even under this severe overestimation and applying a standard 1% market penetration[50] factor for emerging functional textiles and 95% wastewater treatment plant (WWTP) removal efficiency[51], the calculated PEC is 0.19 ppb. This theoretical upper value is significantly lower than the suggested 0.63 ppb[52] safety

threshold, confirming that the environmental risk is negligible. Calculation details are listed with reference (Supplementary Table 9).

Consequently, both experimental evidence (DLS/SEM) and the worst-case theoretical model demonstrate that the mass loss detected by the scale does not translate into hazardous nanoparticle release.

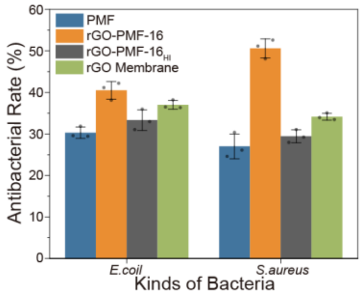

Supplementary Fig. 29 | The antibacterial rate of PMF, rGO-PMF-16 rGO-PMF-16$_{HI}$ and rGO membrane for *Escherichia coli* (*E.coil*) and *Staphylococcus aureus* (*S.aureus*) (n=3). All data in this figure is presented as mean ± SD.

According to the data in Supplementary Fig. 29, rGO-PMF-16 obtains the highest antibacterial rate among all the 4 samples. However, the performance of rGO-PMF-16$_{HI}$ is low, similar to the plain PMF itself. It is because the high reduction eliminates most oxygen-containing functional groups and defects, which leads to the lack of glutathione oxidation effect. High reduction brings obvious hydrophobicity and causes less bacteria to attach to the surface leading to deteriorating the nano knife effect. Moreover, the rGO membrane's performance of bacteriostasis shows the effect of rGO itself. rGO-PMF-16 exhibits the promotion of bacteriostasis performance by coaxial structure's large surface area.

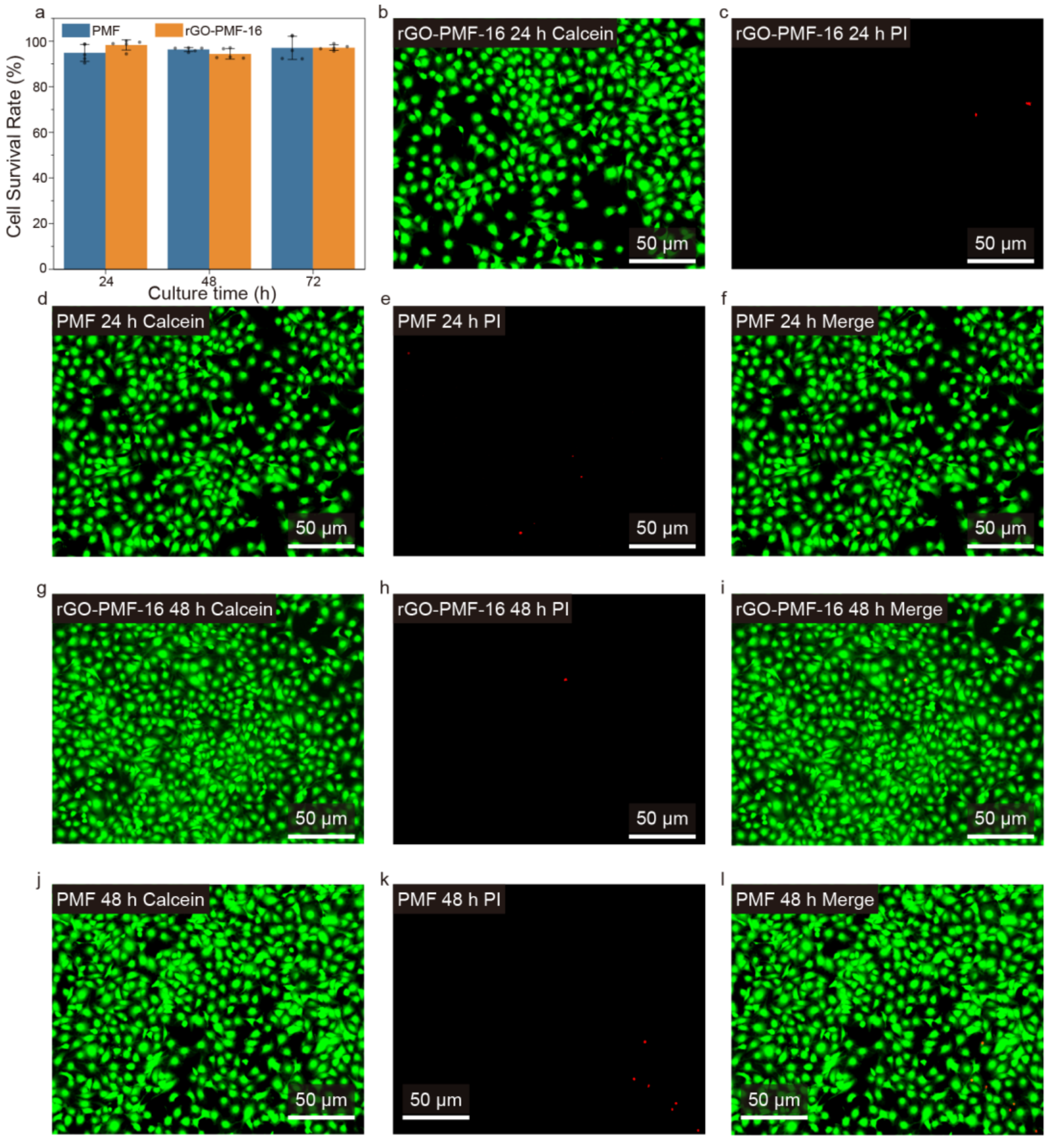

Supplementary Fig. 30 | The cell survival rate of PMFs and the fluorescence microscopy image of human fibroblasts (HFB) cultivated with PMFs. a, The cell survival rate of PMF and rGO-PMF-16 (n=5). All data in this figure is presented as mean ± SD. The calcein fluorescence microscopy image of HFB cultivated with rGO-PMF-16 in **b)** 24 and **g)** 48h. The propidium iodine (PI) fluorescence microscopy image of HFB cultivated with PMF in **c)** 24 and **h)** 48h. The merge fluorescence microscopy image of HFB cultivated with PMF in **i)** 48h. The calcein fluorescence microscopy image of HFB cultivated with PMF in **d)** 24 and **j)** 48h. The propidium iodine (PI) fluorescence microscopy image of HFB cultivated with rGO-PMF-16 in **e)** 24 and **k)** 48h. The merge fluorescence microscopy image of HFB cultivated with rGO-PMF-16 **f)** 24 and **l)** 48h.

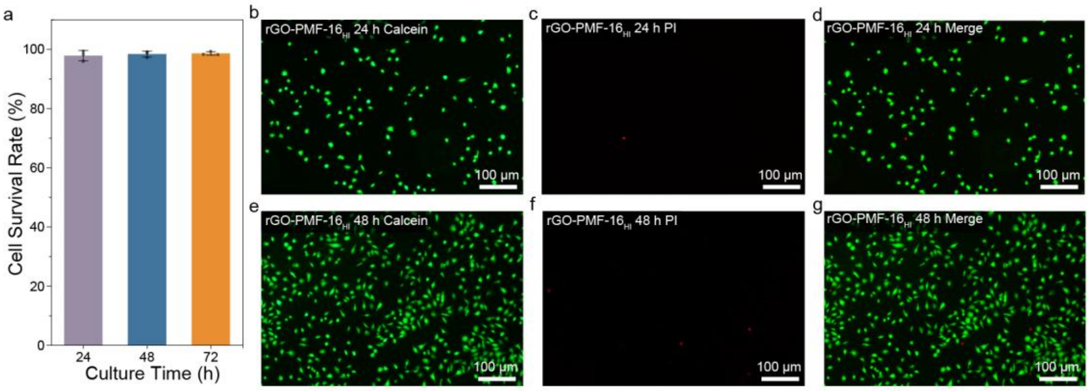

Supplementary Fig. 31 | The cell survival rate of rGO-PMF-16$_{HI}$ and the fluorescence microscopy image of human fibroblasts (HFB) cultivated with rGO-PMF-16$_{HI}$. a, The cell survival rate of rGO-PMF-16$_{HI}$ (n=3). All data in this figure is presented as mean ± SD. The calcein fluorescence microscopy image of HFB cultivated with r rGO-PMF-16$_{HI}$ in **b)** 24 and **e)** 48h. The propidium iodine (PI) fluorescence microscopy image of HFB cultivated with rGO-PMF-16$_{HI}$ in **c)** 24 and **f)** 48h. The merge fluorescence microscopy image of HFB cultivated with rGO-PMF-16$_{HI}$ **d)** 24 and **g)** 48h.

For our specific application of wearable E-fabrics designed for topical skin contact, the primary and initial biological interaction occurs with the skin's outer layers. In this context, human dermal fibroblasts are the most relevant and scientifically justified cell model. They are the predominant cell type in the dermis and the primary frontline cells that would interact with our material. Our testing strategy aligns with the ISO 10993-5:2009 standard, which recommends the use of mammalian fibroblast lines for initial cytotoxicity screening of devices with skin contact[53]. Besides, fibroblasts are widely used to evaluate the biocompatibility among recent works about wearable devices[54–56]. Therefore, we believe that assessing cytotoxicity on fibroblasts provides a direct and relevant safety assessment for the intended use of our material. As presented in Supplementary Fig. 31, rGO-PMF-16$_{HI}$ shows excellent biocompatibility as rGO-PMF-16.

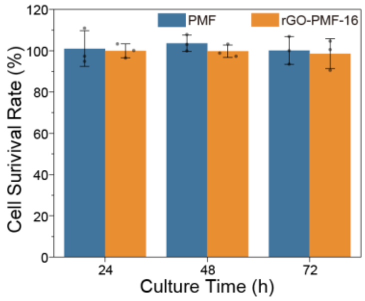

Supplementary Fig. 32 | The cell survival rate of PMF and rGO-PMF-16 in macrophages test (n=3). All data in this figure is presented as mean ± SD.

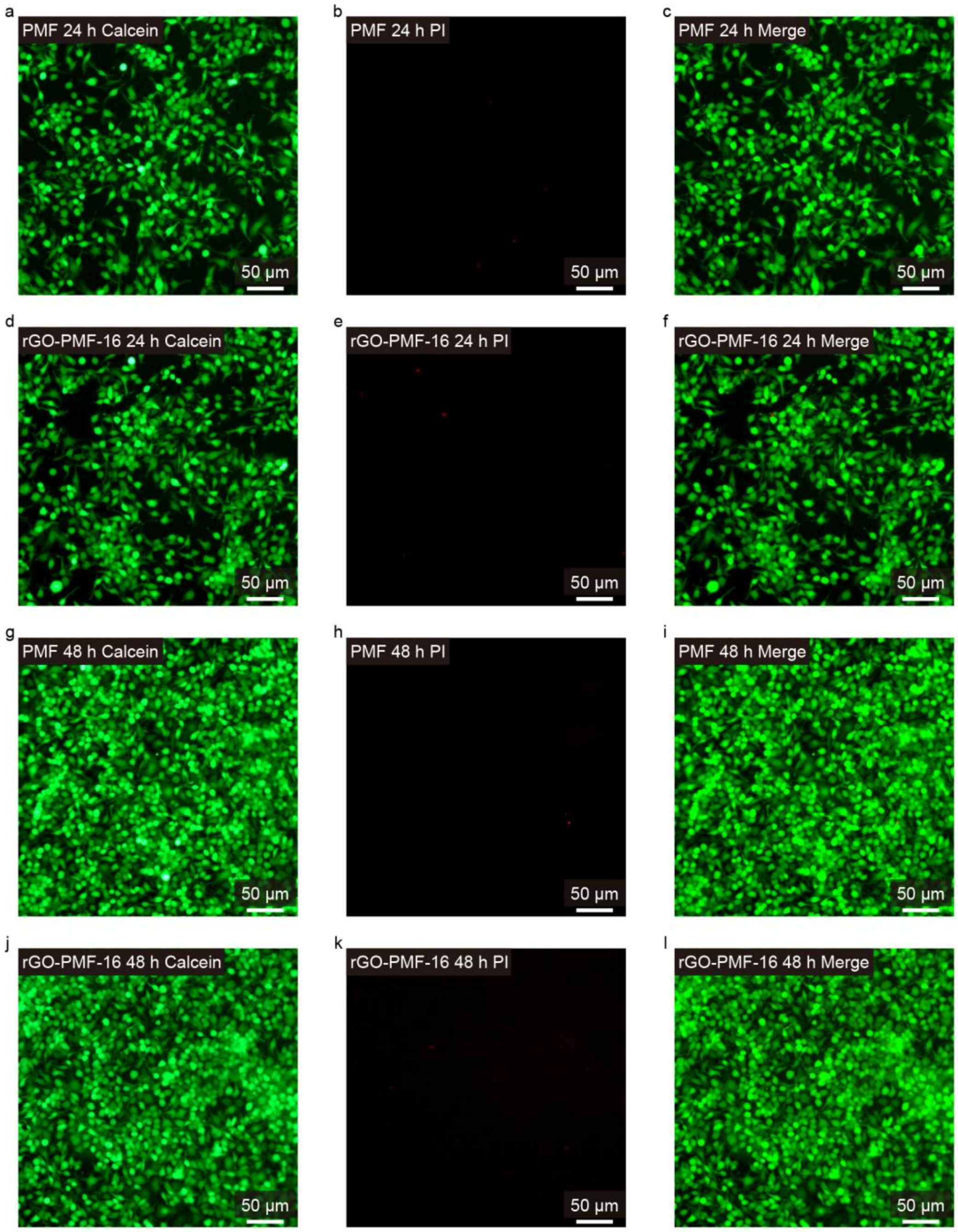

Supplementary Fig. 33 | The fluorescence microscopy image of macrophages cultivated with PMF and rGO-PMF-16. The calcein fluorescence microscopy image of macrophages cultivated with PMF in **a)** 24 and **g)** 48h. The propidium iodine (PI) fluorescence microscopy image of macrophages cultivated with PMF in **b)** 24 and **h)** 48h. The merge fluorescence microscopy image of macrophages cultivated with PMF in **c)** 24 and **i)** 48h. The calcein fluorescence microscopy image of macrophages cultivated with rGO-PMF-16 in **d)** 24 and **j)** 48h. The propidium iodine (PI)

fluorescence microscopy image of macrophages cultivated with rGO-PMF-16 in **e)** 24 and **k)** 48h. The merge fluorescence microscopy image of macrophages cultivated with rGO-PMF-16 **f)** 24 and **l)** 48h.

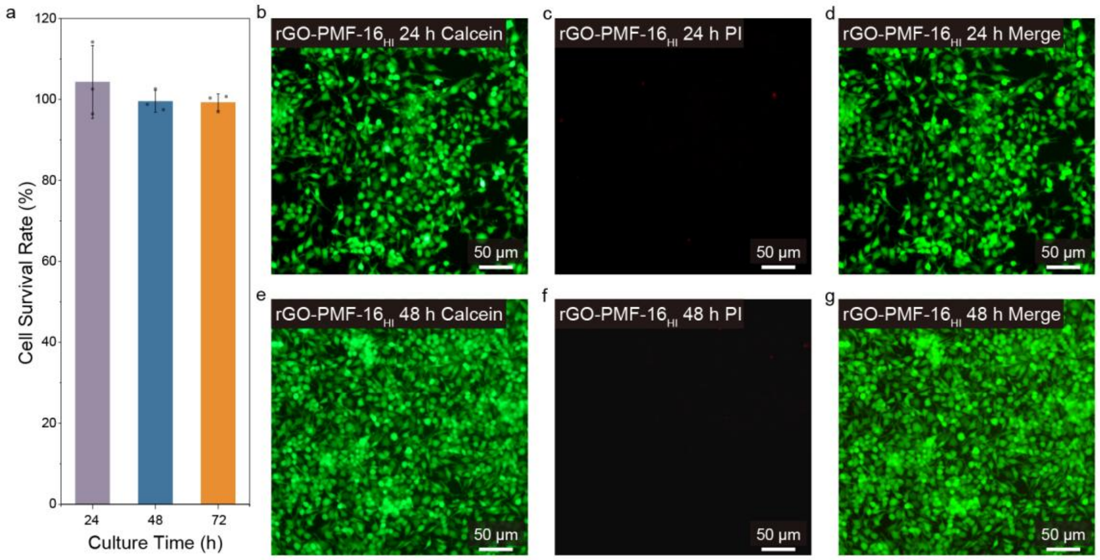

Supplementary Fig. 34 | The cell survival rate of rGO-PMF-16$_{HI}$ and the fluorescence microscopy image of macrophages cultivated with rGO-PMF-16$_{HI}$. a, The cell survival rate of rGO-PMF-16$_{HI}$ (n=3). All data in this figure is presented as mean ± SD. The calcein fluorescence microscopy image of macrophages cultivated with r rGO-PMF-16$_{HI}$ in **b)** 24 and **e)** 48h. The propidium iodine (PI) fluorescence microscopy image of macrophages cultivated with rGO-PMF-16$_{HI}$ in **c)** 24 and **f)** 48h. The merge fluorescence microscopy image of macrophages cultivated with rGO-PMF-16$_{HI}$ **d)** 24 and **g)** 48h.

Evaluating the interaction with immune cells is critical for ensuring the biosafety of wearable skin-electronics. To thoroughly address this, we conduct biocompatibility tests using macrophages co-cultured with PMF, rGO-PMF-16, and rGO-PMF-16$_{HI}$. We perform both quantitative cell viability assays (up to 72 h) and qualitative Live/Dead fluorescence staining (Supplementary Fig. 32-34).

As shown in Supplementary Fig. 32 and Supplementary Fig. 34a, the quantitative results demonstrate that macrophages maintained a high survival rate (near 100%) across all groups after 24, 48, and 72 hours of incubation, showing no significant difference compared to the control. Furthermore, fluorescence microscopy images (Calcein-AM/PI staining, Supplementary Fig. 33 and Supplementary Fig. 34) reveal a high density of viable cells (green fluorescence) with negligible dead cells (red

fluorescence). The cells displayed healthy spreading morphology, indicating excellent proliferation behavior.

These combined results confirm that the rGO-coated fabrics exhibit negligible cytotoxicity to immune cells, strongly supporting their biocompatibility and safety for long-term skin-contact applications.

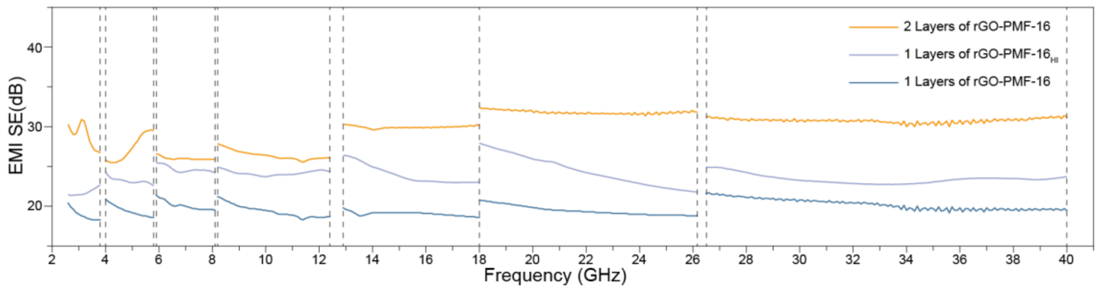

Supplementary Fig. 35 | The EMI SE of rGO-PMF-16 and rGO-PMF-16$_{HI}$ in 1 and 2 layers from 2.6-40.0 GHz.

The Electromagnetic Interference Shielding Effectiveness (EMI SE) of rGO-PMF-16$_{HI}$ is measured across the 2.6–40.0 GHz. As presented in Supplementary Fig. 35, 1 layer of rGO-PMF-16$_{HI}$ exhibits an average EMI SE of approximately 25 dB, representing an improvement of ~5 dB over rGO-PMF-16. This enhancement, however, is modest relative to the fourfold increase in electrical conductivity and remains inferior to the shielding achieved by simply adding a second layer of rGO-PMF-16. These results underscore a diminishing return in EMI SE performance with escalating conductivity. Consequently, the pursuit of ultrahigh conductivity—often at the expense of other wearable properties—yields limited functional gains in shielding performance.

4. Supplementary References

1. Gabbett, C. *et al.* Understanding how junction resistances impact the conduction mechanism in nano-networks. *Nat. Commun.* **15**, 4517 (2024).
2. Kelly, A. G., O'Suilleabhain, D., Gabbett, C. & Coleman, J. N. The electrical conductivity of solution-processed nanosheet networks. *Nat. Rev. Mater.* **7**, 217–234 (2022).
3. TRITON™ X-15 Surfactant | Dow Inc. https://www.dow.com/en-us/pdp.triton-x-15-surfactant.85740z.html#properties.
4. TRITON™ X-45 Surfactant | Dow Inc. https://www.dow.com/en-us/pdp.triton-x-45-surfactant.85765z.html?productCatalogFlag=1#properties.
5. TRITON™ X-100 Surfactant | Dow Inc. https://www.dow.com/en-us/pdp.triton-x-100-surfactant.85735z.html?productCatalogFlag=1#properties.
6. TRITON™ X-165 (70% Actives) Surfactant | Dow Inc. https://www.dow.com/en-us/pdp.triton-x-165-70-actives-surfactant.85743z.html#properties.
7. TRITON™ X-305 (70% Actives) Surfactant | Dow Inc. https://www.dow.com/en-us/pdp.triton-x-305-70-actives-surfactant.85759z.html#properties.
8. Guo, H., Huang, J., Ye, Y., Huang, J. & Wang, X. Surface modification of polypropylene by the entrapping method using the short-chained stearyl-alcohol poly(ethylene oxide) ether modifier. *J. Appl. Polym. Sci.* **133**, (2016).
9. Adams, H., Carver, F. J., Hunter, C. A., Morales, J. C. & Seward, E. M. Chemical Double-Mutant Cycles for the Measurement of Weak Intermolecular Interactions: Edge-to-Face Aromatic Interactions. *Angew. Chem. Int. Ed. Engl.* **35**, 1542–1544 (1996).
10. Lee, E. C. *et al.* Substituent effects on the edge-to-face aromatic interactions. *J. Am. Chem. Soc.* **127**, 4530–4537 (2005).
11. Ringer, A. L., Sinnokrot, M. O., Lively, R. P. & Sherrill, C. D. The Effect of Multiple Substituents on Sandwich and T-Shaped π–π Interactions. *Chemistry – A European Journal* **12**, 3821–3828 (2006).
12. Authorisation List - ECHA. https://echa.europa.eu/authorisation-list.

13. Regulations in the European Union for the Use of Triton X-100 in the Pharmaceutical Industry | BDO. https://www.bdo.com/insights/industries/life-sciences/regulations-in-the-european-union-for-the-use-of-triton-x-100-in-the-pharmaceutical-industry (2022).

14. Tan, J. *et al.* One-pot and industrial manufacturing of cardanol-based polyoxyethylene ether carboxylates as efficient and improved migration resistance plasticizers for PVC. *J. Clean. Prod.* **375**, 133943 (2022).

15. Tyman, J. H. P. & Bruce, I. E. Surfactant properties and biodegradation of polyethoxylates from phenolic lipids. *J. Surfactants Deterg.* **7**, 169–173 (2004).

16. Xi, Z., Xia, T., Shen, L. & Suo, L. Synthesis of cardanol grafted hydrophilic polymers and its mechanism of coal dust inhibition. *Fuel* **345**, 128112 (2023).

17. Toray Carbon Paper 060, Wet Proofed. https://www.fuelcellstore.com/toray-carbon-paper-060.

18. Wang, Y., Wang, C.-Y. & Chen, K. S. Elucidating differences between carbon paper and carbon cloth in polymer electrolyte fuel cells. *J. Electacta.* **52**, 3965–3975 (2007).

19. Hasan, M. M. *et al.* Functionalization of polypropylene nonwoven fabrics using cold plasma (O2) for developing graphene-based wearable sensors. *Sens. Actuators A-Phys.* **300**, 111637 (2019).

20. Pan, Q., Shim, E., Pourdeyhimi, B. & Gao, W. Highly Conductive Polypropylene-Graphene Nonwoven Composite via Interface Engineering. *Langmuir* **33**, 7452–7458 (2017).

21. Sun, T. *et al.* Self-Reinforced Polypropylene/Graphene Composite with Segregated Structures to Achieve Balanced Electrical and Mechanical Properties. *Ind. Eng. Chem. Res.* **59**, 11206–11218 (2020).

22. Neves, A. I. S. *et al.* Towards conductive textiles: coating polymeric fibres with graphene. *Sci. Rep.* **7**, 4250 (2017).

23. Mohan, V. B. & Bhattacharyya, D. Mechanical characterization of functional graphene nanoplatelets coated natural and synthetic fiber yarns using polymeric binders. *Int. J. Smart Nano Mater.* **11**, 78–91 (2020).

24. Zhao, H. *et al.* Fast and facile graphene oxide grafting on hydrophobic polyamide fabric

via electrophoretic deposition route. *J. Mater. Sci.* **53**, 9504–9520 (2018).

25. Yang, L., Pan, L., Xiang, H., Fei, X. & Zhu, M. Organic-Inorganic Hybrid Conductive Network to Enhance the Electrical Conductivity of Graphene-Hybridized Polymeric Fibers. *Chem. Mate.* **34**, 2049–2058 (2022).
26. Liang, B. *et al.* Fabrication and application of flexible graphene silk composite film electrodes decorated with spiky Pt nanospheres. *Nanoscale* **6**, 4264–4274 (2014).
27. Hu, X., Tian, M., Qu, L., Zhu, S. & Han, G. Multifunctional cotton fabrics with graphene/polyurethane coatings with far-infrared emission, electrical conductivity, and ultraviolet-blocking properties. *Carbon N. Y.* **95**, 625–633 (2015).
28. Zulan, L. *et al.* Reduced Graphene Oxide Coated Silk Fabrics with Conductive Property for Wearable Electronic Textiles Application. *Adv. Electron. Mater.* **5**, 1800648 (2019).
29. Karim, N. *et al.* Scalable Production of Graphene-Based Wearable E-Textiles. *ACS Nano* **11**, 12266–12275 (2017).
30. Yapici, M. K., Alkhidir, T., Samad, Y. A. & Liao, K. Graphene-clad textile electrodes for electrocardiogram monitoring. *Sens. Actuators B Chem.* **221**, 1469–1474 (2015).
31. Yun, Y. J. *et al.* A Novel Method for Applying Reduced Graphene Oxide Directly to Electronic Textiles from Yarns to Fabrics. *Adv. Mater.* **25**, 5701–5705 (2013).
32. Karim, N. *et al.* All inkjet-printed graphene-based conductive patterns for wearable e-textile applications. *J. Mater. Chem. C Mater.* **5**, 11640–11648 (2017).
33. Javed, K., Galib, C. M. A., Yang, F., Chen, C. M. & Wang, C. A new approach to fabricate graphene electro-conductive networks on natural fibers by ultraviolet curing method. *Synth. Met.* **193**, 41–47 (2014).
34. Zhou, Q., Ye, X., Wan, Z. & Jia, C. A three-dimensional flexible supercapacitor with enhanced performance based on lightweight, conductive graphene-cotton fabric electrode. *J. Power Sources* **296**, 186–196 (2015).
35. Xu, L. L., Guo, M. X., Liu, S. & Bian, S. W. Graphene/cotton composite fabrics as flexible electrode materials for electrochemical capacitors. *RSC Adv.* **5**, 25244–25249 (2015).
36. Afroj, S., Tan, S., Abdelkader, A. M., Novoselov, K. S. & Karim, N. Highly Conductive,

Scalable, and Machine Washable Graphene-Based E-Textiles for Multifunctional Wearable Electronic Applications. *Adv. Funct. Mater.* **30**, 2000293 (2020).

37. Abdelkader, A. M. *et al.* Ultraflexible and robust graphene supercapacitors printed on textiles for wearable electronics applications. *2D Mater.* **4**, 035016 (2017).

38. Molina, J. *et al.* Electrochemical characterization of reduced graphene oxide-coated polyester fabrics. *J. Electacta.* **93**, 44–52 (2013).

39. Shateri-Khalilabad, M. & Yazdanshenas, M. E. Preparation of superhydrophobic electroconductive graphene-coated cotton cellulose. *Cellulose* **20**, 963–972 (2013).

40. Fugetsu, B., Sano, E., Yu, H., Mori, K. & Tanaka, T. Graphene oxide as dyestuffs for the creation of electrically conductive fabrics. *Carbon N. Y.* **48**, 3340–3345 (2010).

41. Samad, Y. A., Li, Y., Alhassan, S. M. & Liao, K. Non-destroyable graphene cladding on a range of textile and other fibers and fiber mats. *RSC Adv.* **4**, 16935–16938 (2014).

42. Sahito, I. A., Sun, K. C., Arbab, A. A., Qadir, M. B. & Jeong, S. H. Graphene coated cotton fabric as textile structured counter electrode for DSSC. *J. Electacta.* **173**, 164–171 (2015).

43. Guo, Q. *et al.* Structural–Functional Integrated Graphene-Skinned Aramid Fibers for Electromagnetic Interference Shielding. *ACS Nano* **18**, 33566–33575 (2024).

44. Ma, Z. *et al.* Permeable superelastic liquid-metal fibre mat enables biocompatible and monolithic stretchable electronics. *Nat. Mater.* **20**, 859–868 (2021).

45. Yin, L. *et al.* A self-sustainable wearable multi-modular E-textile bioenergy microgrid system. *Nat. Commun.* **12**, (2021).

46. Libanori, A., Chen, G., Zhao, X., Zhou, Y. & Chen, J. Smart textiles for personalized healthcare. *Nat. Electron.* **5**, 142–156 (2022).

47. Liang, X. *et al.* Hydrophilic, Breathable, and Washable Graphene Decorated Textile Assisted by Silk Sericin for Integrated Multimodal Smart Wearables. *Adv. Funct. Mater.* **32**, 2200162 (2022).

48. Ma, X. *et al.* A monolithically integrated in-textile wristband for wireless epidermal biosensing. *Sci. Adv.* **9**, eadj2763 (2023).

49. Guidance on information requirements and Chemical Safety Assessment Chapter R.16:

Environmental exposure assessment. https://echa.europa.eu/documents/10162/17224/IR_CSR_R16_V4_FINAL.pdf/b9f0f406-ff5f-4315-908e-e5f83115d6af (2026).

50. Ema. Committee for Medicinal Products for Human Use (CHMP) Guideline on the environmental risk assessment of medicinal products for human use. https://www.ema.europa.eu/en/documents/scientific-guideline/guideline-environmental-risk-assessment-medicinal-products-human-use-revision-1_en.pdf (2024).

51. Registry of restriction intentions until outcome - ECHA. https://echa.europa.eu/registry-of-restriction-intentions/-/dislist/details/0b0236e18244cd73.

52. Fekete-Kertész, I. *et al.* Assessing the Chronic Environmental Risk of Graphene Oxide Using a Multimarker Approach Across Three Trophic Levels of the Aquatic Ecosystem. *Nanomaterials* **15**, 1553 (2025).

53. Gruber, S. & Nickel, A. Toxic or not toxic? The specifications of the standard ISO 10993-5 are not explicit enough to yield comparable results in the cytotoxicity assessment of an identical medical device. *Front. Med. Technol.* **5**, 1195529 (2023).

54. Ye, G., Song, D., Song, J., Zhao, Y. & Liu, N. A Fully Biodegradable and Biocompatible Ionotronic Skin for Transient Electronics. *Adv. Funct. Mater.* **33**, 2303990 (2023).

55. Chen, S. *et al.* Mechanically and biologically skin-like elastomers for bio-integrated electronics. *Nat. Commun.* **11**, 1–8 (2020).

56. Cheng, S. *et al.* Ultrathin Hydrogel Films toward Breathable Skin-Integrated Electronics. *Adv. Mater.* **35**, 2206793 (2023).
