# Supplementary material for: Conformal graphene coatings on ordinary fabrics for wearable electronic devices
Source: Nat Commun. 2026 May 18;17:6565. doi: 10.1038/s41467-026-73319-2 (PMC13381529; doi:10.1038/s41467-026-73319-2)
Supplement: Supplementary file 2 — Description of Additional Supplementary Files [file 41467_2026_73319_MOESM2_ESM.pdf]

Description of Additional Supplementary Files

Supplementary Video 1 | Molecular dynamics simulation video of Triton's attachment in EA.

Supplementary Video 2 | Molecular dynamics simulation video of Triton's attachment in DI water.
