# Supplementary material for: Conformal graphene coatings on ordinary fabrics for wearable electronic devices
Source: Nat Commun. 2026 May 18;17:6565. doi: 10.1038/s41467-026-73319-2 (PMC13381529; doi:10.1038/s41467-026-73319-2)
Supplement: Supplementary file 5 — Reporting Summary [file 41467_2026_73319_MOESM5_ESM.pdf]

nature portfolio

Corresponding author(s): Bo Liu, Jiaxing Huang, Daping He

Last updated by author(s): Mar 20, 2026

Reporting Summary

Nature Portfolio wishes to improve the reproducibility of the work that we publish. This form provides structure for consistency and transparency in reporting. For further information on Nature Portfolio policies, see our Editorial Policies and the Editorial Policy Checklist.

Statistics

For all statistical analyses, confirm that the following items are present in the figure legend, table legend, main text, or Methods section.

n/a	Confirmed	
☐	☒	The exact sample size (*n*) for each experimental group/condition, given as a discrete number and unit of measurement
☐	☒	A statement on whether measurements were taken from distinct samples or whether the same sample was measured repeatedly
☒	☐	The statistical test(s) used AND whether they are one- or two-sided<br>*Only common tests should be described solely by name; describe more complex techniques in the Methods section.*
☒	☐	A description of all covariates tested
☒	☐	A description of any assumptions or corrections, such as tests of normality and adjustment for multiple comparisons
☐	☒	A full description of the statistical parameters including central tendency (e.g. means) or other basic estimates (e.g. regression coefficient) AND variation (e.g. standard deviation) or associated estimates of uncertainty (e.g. confidence intervals)
☒	☐	For null hypothesis testing, the test statistic (e.g. *F*, *t*, *r*) with confidence intervals, effect sizes, degrees of freedom and *P* value noted<br>*Give P values as exact values whenever suitable.*
☒	☐	For Bayesian analysis, information on the choice of priors and Markov chain Monte Carlo settings
☒	☐	For hierarchical and complex designs, identification of the appropriate level for tests and full reporting of outcomes
☒	☐	Estimates of effect sizes (e.g. Cohen's *d*, Pearson's *r*), indicating how they were calculated

Our web collection on statistics for biologists contains articles on many of the points above.

Software and code

Policy information about availability of computer code

Data collection	FORCITE package of Materials Studio software 2020；COMPASS Ⅲ
Data analysis	OriginPro 2025b (OriginPro Learning Edition), Adobe Illustrator 2026

For manuscripts utilizing custom algorithms or software that are central to the research but not yet described in published literature, software must be made available to editors and reviewers. We strongly encourage code deposition in a community repository (e.g. GitHub). See the Nature Portfolio guidelines for submitting code & software for further information.

Data

Policy information about availability of data

All manuscripts must include a data availability statement. This statement should provide the following information, where applicable:

- Accession codes, unique identifiers, or web links for publicly available datasets
- A description of any restrictions on data availability
- For clinical datasets or third party data, please ensure that the statement adheres to our policy

All other data supporting the findings of this study are available in the main text and the Supplementary Information. The data generated in this study are provided in the Source Data file.

Research involving human participants, their data, or biological material

Policy information about studies with human participants or human data. See also policy information about sex, gender (identity/presentation), and sexual orientation and race, ethnicity and racism.

Reporting on sex and gender	N/A. This article involves in vitro experiments using established commercial human cell lines, not human participants.
Reporting on race, ethnicity, or other socially relevant groupings	N/A. This article does not involve reporting on race, ethnicity, or other socially relevant groupings.
Population characteristics	N/A. This article does not involve population characteristics.
Recruitment	N/A. This article does not involve recruitment.
Ethics oversight	N/A. This article does not involve ethics oversight.

Note that full information on the approval of the study protocol must also be provided in the manuscript.

Field-specific reporting

Please select the one below that is the best fit for your research. If you are not sure, read the appropriate sections before making your selection.

- [x] Life sciences
- [ ] Behavioural & social sciences
- [ ] Ecological, evolutionary & environmental sciences

For a reference copy of the document with all sections, see nature.com/documents/nr-reporting-summary-flat.pdf

Life sciences study design

All studies must disclose on these points even when the disclosure is negative.

Sample size	No statistical methods were used to predetermine sample size. Sample sizes were chosen based on standard practices in the field for in vitro antibacterial and biocompatibility assays (n=3/5, noted in figure legends), which have been historically shown to be sufficient for statistical significance in these types of experiments.
Data exclusions	No data were excluded from the analyses.
Replication	All experiments were performed in at least three independent replicates. All attempts at replication were successful and yielded reproducible results.
Randomization	Samples, bacterial cultures, and cell lines were randomly allocated into experimental and control groups prior to all assays.
Blinding	Investigators were not blinded to group allocation during the in vitro cell and bacterial experiments, as the physical presence and distinct appearance of the material samples in culture vessels make blinding practically impossible. To strictly prevent any subjective bias, all quantitative data were acquired using standardized, automated instruments without manual intervention in the readout process.

Reporting for specific materials, systems and methods

We require information from authors about some types of materials, experimental systems and methods used in many studies. Here, indicate whether each material, system or method listed is relevant to your study. If you are not sure if a list item applies to your research, read the appropriate section before selecting a response.

Materials & experimental systems

n/a	Involved in the study	
x		Antibodies
	x	Eukaryotic cell lines
x		Palaeontology and archaeology
	x	Animals and other organisms
x		Clinical data
x		Dual use research of concern
x		Plants

Methods

n/a	Involved in the study	
x		ChIP-seq
x		Flow cytometry
x		MRI-based neuroimaging

Eukaryotic cell lines

Policy information about cell lines and Sex and Gender in Research

Cell line source(s)	Human fibroblasts (HSF, STM-CL-5176, Male, Adult) were obtained from Stemrecell (Shanghai) Biotechnology Co., Ltd. Mouse

	macrophages (RAW 264.7, CL-0190, Male, Adult) were obtained from Wuhan Pricella Biotechnology Co., Ltd.
Authentication	The cell lines were authenticated by the supplier prior to purchase. No further authentication was performed in our laboratory.
Mycoplasma contamination	The cell lines were not specifically tested for mycoplasma contamination in our laboratory.
Commonly misidentified lines (See ICLAC register)	None of the cell lines used in this study are listed in the ICLAC database of commonly misidentified cell lines.

Animals and other research organisms

Policy information about studies involving animals; ARRIVE guidelines recommended for reporting animal research, and Sex and Gender in Research

Laboratory animals	This study did not involve laboratory animals. For in vitro antibacterial assays, standard bacterial strains including Escherichia coli (BNCC133264) and Staphylococcus aureus (BNCC186335) were utilized and obtained from BeNa Culture Collection (Beijing, China).
Wild animals	N/A. This study did not involve wild animals.
Reporting on sex	N/A.
Field-collected samples	N/A. This study did not involve field-collected samples.
Ethics oversight	No ethical approval or guidance was required, as the study solely utilized commercially available, established human cell lines and standard bacterial strains for in vitro experiments.

Note that full information on the approval of the study protocol must also be provided in the manuscript.

Plants

Seed stocks	N/A
Novel plant genotypes	N/A
Authentication	N/A
