# Supplementary material for: Conformal graphene coatings on ordinary fabrics for wearable electronic devices
Source: Nat Commun. 2026 May 18;17:6565. doi: 10.1038/s41467-026-73319-2 (PMC13381529; doi:10.1038/s41467-026-73319-2)
Supplement: Supplementary file 6 — Transparent Peer Review file [file 41467_2026_73319_MOESM6_ESM.pdf]

nature portfolio

Peer Review File

Conformal Graphene Coatings on Ordinary Fabrics for Wearable Electronic Devices

Corresponding Author: Professor Daping He

This file contains all reviewer reports in order by version, followed by all author rebuttals in order by version.

Version 0:

Reviewer comments:

Reviewer #1

(Remarks to the Author)
The English language of the paper is not vetted.

Throughout the paper: I am not sure that the term "electrifying" is appropriate. The process here is merely about making a textile (a surface) conductive. There is no electricity production or storage involved.

The product Triton X-100 (octylphenol ethoxylate, OPE) is no longer REACH-compliant in Europe, or will not be in the future. Nonylphenol and its ethoxylated derivatives were added to the CEPA toxic substances list in June 2001. Therefore, it is not feasible to develop an industrial production process for conductive textiles using this product (line 233 and conclusion).

Line 31: You wrote, "various ordinary fabrics." Does this apply to natural fibers (e.g., cotton) or other cellulosic fibers?

Line 36: Be careful—E-textiles are not necessarily wearable and not necessarily advanced.

Reviewer #2

(Remarks to the Author)
In this study, electronic fabrics were fabricated using temporal decoupling strategy with Triton, GO, and PMF. The conductivity of the rGO-PMF-16 is 283.06 S/m and the microscopic structures were provided. The authors compared the conductivity with various surfactants and solvents. The biocompatibility, bacteriostasis, hydrophilicity, air-permeability, washability, EMI shielding, and Joule heating were provided. The mass production of the fabric was achieved. Molecular dynamics simulation was also provided for interpreting the mechanism of the strategy.

POINT-BY-POINT COMMENTS
1. The conductivity of 283.06 S/m of rGO-PMF-16 is very small compared to the other e-textiles fabricated with rGO (1040 S/m in Adv. Mater. 2013, 24, 5701, 1163 S/m in Adv. Mater. 2017, 29, 160479, etc).
2. Moreover, this value of conductivity was not shown in any materials including Supplementary Information.
3. Different swelling times- (Fig. S11), different GO concentrations- (Fig. S12), single dipping time- (Fig. S13), and different reduction times- (Fig. S14) dependent conductivity of rGO-PMF-16 should be provided instead of the conductivity of rGO-PMF-1 and rGO-PMF-4.
4. The authors wrote that the bridge and bunding structures cause to enhance the conductivity. If then, the highest conductivity of rGO-PMF-16 originates from the large electron pathway due to the 16 dip coating. In this regard, the air permeability of rGO-PMFs (Fig. S17) is not understandable.
5. In the same way, the highest conductivity should be shown in rGO-PMF fabricated large size of GO (1016.29 nm) but not 401.53 nm of GO (Figs. S6 and S8). A profound physical interpretation should be provided.
6. The rGO is the main factor to endow the electrical conductivity of rGO-PMFs. If then, what is the reason for the different conductivity of various polymers shown in Fig. 4?
7. 1214.84 S/m of conductivity was achieved using HI acid but it had low hydrophilicity. How about air permeability, EMI

shielding, bacteriostasis, and biocompatibility of this sample? Occasionally, hydrophobicity is more important than hydrophilicity.
8. The reduction methods cause different conductivities as mentioned in comment 7. Hence the rGO is the main factor but not microscopic morphology as the authors mentioned on page 20 of Supplementary Information.
9. The antibacterial rate of rGO-PMF-16 is higher than that of PMF. Inserting the data of rGO itself helps the reader understand the role of rGO.

In this light, there is no novelty for the publication in Nature Communications.

Reviewer #3

(Remarks to the Author)

General Comments for Main Text:

1. **Clarification on Triton Type**: The study mentions the use of Triton, but there are various types within the Triton family. Please specify which type of Triton was used and provide a rationale for its selection.

2. **Justification for Choosing Triton**: While Triton is a well-known non-ionic surfactant, there are many other non-ionic surfactants available. Could you elaborate on why Triton was chosen over other alternatives?

3. **Experimental Design**: The current study utilized fabric directly for coating. To strengthen the evidence for the mechanisms proposed by the authors, it is recommended to perform additional experiments using single fibers with larger diameters. This approach could provide more detailed insights into the interactions and validate the proposed mechanisms more effectively.

4. **Reallocation of Data**: The main text currently consists mainly of pictures and photos, with too many data and results placed in the Supplementary Information (SI). Key results from the SI should be re-allocated back into the main text. For example, Figure 6 contains almost no data and is filled with photos/ pictures. Important data should be included in the main text to enhance the clarity and impact of the study.

5. **Conductivity Calculation**: How was the conductivity calculation performed in this study? Additionally, how does the way the conductivity is deduced compare to other literature? Please provide an explanation and cross-comparison.

6. **Conversion of Costs**: In Figure 5, how are energy costs and carbon costs converted to 'production cost in S.m^-2'? Please clarify the methodology used for these conversions.

7. **Variation in Sheet Resistance**: It is important to state the batch-to-batch variation and position-to-position variation in sheet resistance. This information is crucial for understanding the consistency and reliability of the results.

Comments for Supplementary Information (SI):

1. **Surface Chemical Characterizations**: Perform surface chemical characterizations for the commercial polymer fabrics, as many of these fabrics could have pre-existing surface treatments that significantly affect the functionalization mechanisms and outcomes.

2. **Nanoparticle Release**: Check the nanoparticle release from the bath during machine washing. This is important for assessing the environmental impact and safety of the materials used.

3. **Biocompatibility Testing**: Biocompatibility tests should be performed on macrophages instead of fibroblasts. Macrophages are more relevant for assessing the immune response and biocompatibility of the materials.

4. **Impact of Environmental Conditions**: How do the data differ when operating under different humidity and temperature conditions? Provide an analysis of the impact of these environmental factors on the results.

5. **Raw Data for Fabric Weights**: In SI-Figure 18, the loss of weight is reported to be between 0 to 2%. Please provide the raw data on the fabric weights that derive these results for transparency and validation.

Version 1:

Reviewer comments:

Reviewer #1

(Remarks to the Author)
The authors have taken into account the reviewers’ numerous comments. The responses are well structured, address the

questions, and improve the overall quality of the manuscript. A substantial amount of supplementary material has been produced, and the bibliography has been expanded. I have only one point to suggest for revision: the number of significant figures reported for some values. In several cases, the stated precision is unnecessary given the units, standard deviations, or number of measurements. Values with a higher number of significant figures may be presented in the Supplementary Material or in the raw data.

Reviewer #2

(Remarks to the Author)
The authors have revised the manuscript as the reviewer commented. Hence, the revised manuscript is scientifically crystal clear. In this regard, the manuscript has novelty for publication in Nature Communications.

I examined the response to the comment from Reviewer 3. The answers are insufficient for the questions. The reason is as follows.

Answer 1. Chemical formula is needed. HLB values of other tritons in Table R3 should be checked again.
Answer 3. The FE-SEM or FE-TEM image of the cross-section of the single fiber with larger diameters and the electrical conductivity of the single fiber have to be provided.
Answers 5 and 7. The most important thing is that the conductivity obtained from the sheet resistance with the thickness can not interpret the conductive pathway along the z-direction. It is just a conventional 4-probe measurement. This method just provides in-plane conductivity.
The conductivity is over 103 S/m in Figure 5d, which is not consistent with answer 7 (283.6 S/m).
In SI,
Answer 2. How to measure total weight loss? If the authors used the scale, the results are not acceptable because a small amount of the nanoparticles can affect the environment.
Answer 3. I agree with Reviewer 3. The author should perform on macrophages.

Version 2:

Reviewer comments:

Reviewer #2

(Remarks to the Author)
The authors have provided comprehensive responses to the concerns raised during the review process. Consequently, the revised manuscript demonstrates sufficient novelty and scientific merit for publication.

Open Access This Peer Review File is licensed under a Creative Commons Attribution 4.0 International License, which permits use, sharing, adaptation, distribution and reproduction in any medium or format, as long as you give appropriate credit to the original author(s) and the source, provide a link to the Creative Commons license, and indicate if changes were made.

In cases where reviewers are anonymous, credit should be given to 'Anonymous Referee' and the source.
The images or other third party material in this Peer Review File are included in the article’s Creative Commons license, unless indicated otherwise in a credit line to the material. If material is not included in the article’s Creative Commons license and your intended use is not permitted by statutory regulation or exceeds the permitted use, you will need to obtain permission directly from the copyright holder.
To view a copy of this license, visit https://creativecommons.org/licenses/by/4.0/

<u>Replies to the Reviewers' Comments and Revisions Made to the Manuscript</u>

Reviewer: 1

1. The English language of the paper is not vetted.

Response: Thank you for the detailed review. We have had the manuscript professionally edited by a native English-speaking expert to improve its overall clarity and readability.

2. Throughout the paper: I am not sure that the term "electrifying" is appropriate. The process here is merely about making a textile (a surface) conductive. There is no electricity production or storage involved.

Response: We sincerely thank the reviewer for raising this important point. We fully agree that "electrifying" is suboptimal as it may imply energy conversion rather than imparting conductivity. To precisely convey that this work focuses on enabling fabrics with conductivity for use in wearable electronic devices, the title has been revised to "Conformal Graphene Coatings on Ordinary Fabrics for Wearable Electronic Devices."

3. The product Triton X-100 (octylphenol ethoxylate, OPE) is no longer REACH-compliant in Europe, or will not be in the future. Nonylphenol and its ethoxylated derivatives were added to the CEPA toxic substances list in June 2001. Therefore, it is not feasible to develop an industrial production process for conductive textiles using this product (line 233 and conclusion).

Response: We sincerely thank the reviewer for this critical and insightful comment regarding regulatory compliance and environmental impact. We have thoroughly investigated these concerns, and our response addresses them on two key fronts: (1) demonstrating full compliance with current EU regulations, and (2) underscoring an environmentally friendly alternative based on the temporal decoupling (TD) strategy.

1. Compliance with EU REACH Regulations

We have conducted a comprehensive review of laws and regulations in most major industrial countries. Notably, the European Union (EU) stands out as the **<u>only region</u>**

with specific restrictions on Triton X-100 (Triton) and its derivatives. In contrast, Canada's CEPA does not list octyl phenol (the base component of Triton X-100) as a controlled substance[1,2]. We acknowledge the reviewer's accurate reference to the EU REACH restrictions on Triton. Importantly, a key exemption under this regulation specifies that authorization is not required if the substance concentration in the final products remains below 0.1% (w/w)[3,4].

To definitively verify the compliance of rGO-PMFs-16, quantitative analysis is conducted by high-performance liquid chromatography (HPLC). A standard calibration curve with excellent linearity (R^2 = 0.99994) is established through a series of Triton solutions (Figure R1a and c). Triton is extracted from fabric samples (n=3) via Soxhlet extraction (Figure R1b). The results are highly consistent, yielding a calculated concentration of **0.07% (w/w)** of Triton in rGO-PMFs-16. **This value is conclusively below the 0.1% regulatory threshold, confirming that the production and sale of rGO-PMFs-16 are fully compliant with current EU REACH regulations.**

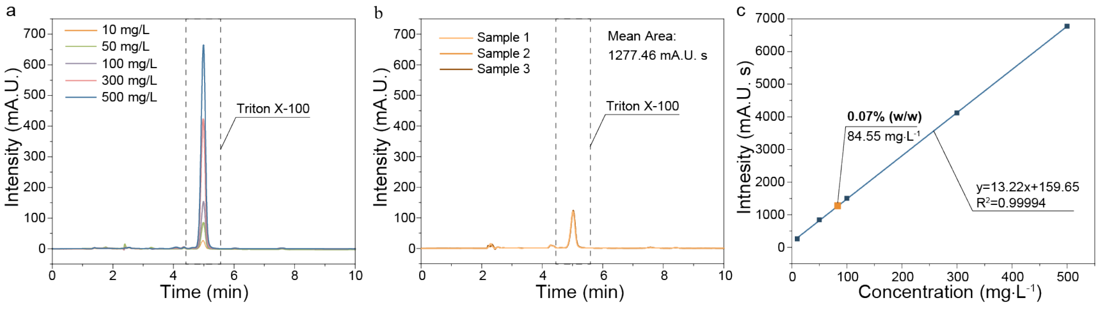

Figure R1 | Triton Concentration of rGO-PMF-16 detected by HPLC. a, The HPLC peaks of Triton at different concentrations. **b,** The HPLC results of Triton in rGO-PMF-16. **c,** The Triton concentration of samples on the linear fit standard curve.

2. An Eco-Friendly Alternative Based on the Proposed TD Strategy

We share the reviewer's underlying concern regarding the ecological footprint of manufacturing. The significance of our work lies not in the specific surfactant used, but in the universal TD strategy. To proactively demonstrate this versatility and address environmental considerations, a synthesis is successfully replicated using cardanol polyoxyethylene ether (CPE-9), **a surfactant with a superior environmental profile**[5–7]. The resulting fabrics (rGO-cPMF-x) exhibit a conformal rGO coaxial coating (Figure R2a and b) and achieve high conductivity (up to 153.87 S m^{-1}, Figure R2c). These

results unequivocally prove that TD Strategy is universally adaptable and provides a direct pathway for developing high-performance conductive fabrics with a significantly reduced ecological impact.

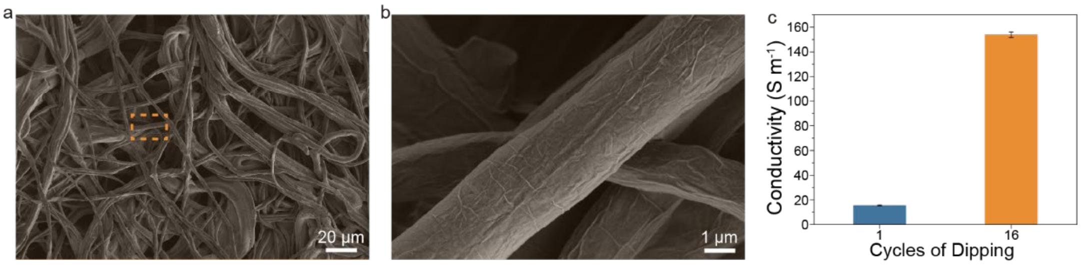

Figure R2 | The synthesis results of rGO-cPMFs. a, The SEM image of rGO-cPMF-16's surface. **b,** The SEM image of marked part in figure R2a. **c,** The conductivity of rGO-cPMF-1 and rGO-cPMF-16.

In conclusion, rGO-PMFs-16 meets all necessary regulatory standards in an industrial production process. More importantly, TD strategy is future-proof, enabling the expansion to more environmentally friendly chemical systems. Figures R1 and R2 have been added to Support Information as Supplementary Fig. 10 and 11, and corresponding discussion has been added accordingly in the manuscript.

Reference

1. Toxic substances list: schedule 1 - Canada.ca. https://www.canada.ca/en/environment-climate-change/services/canadian-environmental-protection-act-registry/substances-list/toxic/schedule-1.html (2025).
2. Screening assessment - Poly(alkoxylates/ethers) Group - Canada.ca. https://www.canada.ca/en/environment-climate-change/services/evaluating-existing-substances/screening-assessment-poly-alkoxylates-ethers-group.html (2022).
3. Authorisation List - ECHA. https://echa.europa.eu/authorisation-list (2025).
4. Regulations in the European Union for the Use of Triton X-100 in the Pharmaceutical Industry | BDO. https://www.bdo.com/insights/industries/life-sciences/regulations-in-the-european-union-for-the-use-of-triton-x-100-in-the-pharmaceutical-industry (2022).
5. Tan, J. *et al.* One-pot and industrial manufacturing of cardanol-based polyoxyethylene ether carboxylates as efficient and improved migration resistance plasticizers for PVC. *J. Clean Prod.* **375**, 133943 (2022).
6. Tyman, J. H. P. & Bruce, I. E. Surfactant properties and biodegradation of polyethoxylates from phenolic lipids. *J. Surfactants Deterg.* **7**, 169–173 (2004).
7. Xi, Z., Xia, T., Shen, L. & Suo, L. Synthesis of cardanol grafted hydrophilic polymers and its mechanism of coal dust inhibition. *Fuel* **345**, 128112 (2023).

4. Line 31: You wrote, "various ordinary fabrics." Does this apply to natural fibers (e.g., cotton) or other cellulosic fibers?

Response: We thank the reviewer for this insightful comment. To directly address the question regarding natural fibers, two representative natural/cellulosic fibers (cotton nonwoven fabrics and linen nonwoven fabrics) are verified. The iconic surface morphology of graphene (especially on marked parts) appears on rGO-fabrics in conformal coating structure (Figure. R3a and b) compared to their raw surface (Figure. R3c and d), confirming a the successful assembly of the coaxial coating on these natural materials.

More importantly, conductivity measurements (Figure. R3e) robustly demonstrate the universality of our TD strategy. The conductivity values of both rGO-cotton and rGO-linen fabricated with the TD strategy are higher than those prepared without it. This consistent performance enhancement across synthetic polymers and now natural fabrics strongly supports the universality of our approach.

The corresponding results are included in the revised manuscript as Figure 4 and Supplementary Fig. 13.

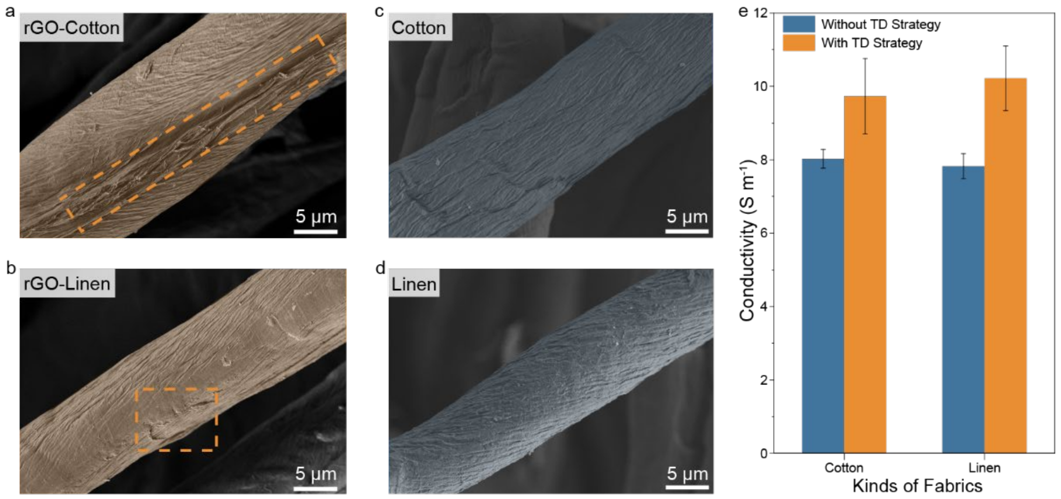

Figure R3 | The universality of TD strategy on natural fabrics. a, The SEM image of cotton nonwoven fabrics in single dip coating treatment. **b,** The SEM image of linen nonwoven fabrics in single dip coating treatment. **c,** The raw fiber SEM image of cotton nonwoven fabrics. **d,** The raw fiber SEM image of linen nonwoven fabrics. **e,** The conductivity of two fabrics with/without TD strategy in single dip coating treatment.

5. Line 36: Be careful—E-textiles are not necessarily wearable and not necessarily advanced.

Response: Thanks for your detailed comments. The sentence in Line 36 is revised as: "Electronic fabrics (E-fabrics), which integrate functional materials into fabrics, hold great potential for creating advanced wearable devices that could revolutionize our interaction with technology."

Reviewer: 2

Comments: In this study, electronic fabrics were fabricated using temporal decoupling strategy with Triton, GO, and PMF. The conductivity of the rGO-PMF-16 is 283.06 S/m and the microscopic structures were provided. The authors compared the conductivity with various surfactants and solvents. The biocompatibility, bacteriostasis, hydrophilicity, air-permeability, washability, EMI shielding, and Joule heating were provided. The mass production of the fabric was achieved. Molecular dynamics simulation was also provided for interpreting the mechanism of the strategy.

Response: We thank the reviewer for insightful comments and constructive suggestions on our manuscript. The feedback, particularly regarding the synthesis mechanism, has been instrumental in helping us deepen our understanding and improve the mechanistic discussion. We have addressed all the points raised and provided detailed, point-by-point responses below. Our revisions, which include a dynamic investigation through multiple dipping cycles and a thorough exploration of the conductivity-morphology balance, have significantly strengthened the manuscript.

1. The conductivity of 283.06 S/m of rGO-PMF-16 is very small compared to the other e-textiles fabricated with rGO (1040 S/m in Adv. Mater. 2013, 24, 5701, 1163 S/m in Adv. Mater. 2017, 29, 160479, etc).

Response: Thank you for this insightful comment and we are glad to clarify the misunderstanding. Notably, while electrical conductivity is a critical parameter for the practical applications of wearable electronic fabrics, it is by no means the sole determinant of performance. These materials must also meet essential wearability requirements, including hydrophilicity, air permeability, washability, bacteriostasis, and biocompatibility[8–10]. In practice, achieving ultra-high conductivity (typically >1000 S m^{-1}) often necessitates aggressive chemical or thermal reduction of graphene oxide (GO), which markedly deteriorates other key attributes vital for wearability—such as hydrophilicity and bacteriostatic properties. As evidenced by the mentioned reference and rGO-PMF-16_{HI} sample (1214.84 S m^{-1}, achieved by HI reduction in this work), such high conductivity often comes at the expense of a holistic

wearable profile. This trade-off represents a typical bottleneck effect in the field, as illustrated in Figure R4 (detailed discussion in Response 7). Therefore, rGO-PMF-16 is engineered to achieve an **optimal balance**, providing substantial conductivity (283.06 S m^{-1}) while retaining hydrophilicity, air permeability, washability, bacteriostasis, and biocompatibility. This balance is crucial, as our EMI shielding analysis reveals that further increasing conductivity yields diminishing returns (<5 dB gain) while severely compromising other wearable properties. As summarized in Table R1, to the best of our knowledge, almost no previous work has achieved a comparable integration of comprehensive wearability features with similarly high conductivity.

Furthermore, temporal decoupling (TD) strategy, which avoids harsh processes while acquiring decent conductivity, is not only gentle on ordinary fabrics but also inherently **scalable and practical for mass production**, addressing a key challenge in transitioning E-fabrics from the lab to real-world applications. The corresponding results are also included in the revised Supplementary Fig. 20 and Supplementary Table 6.

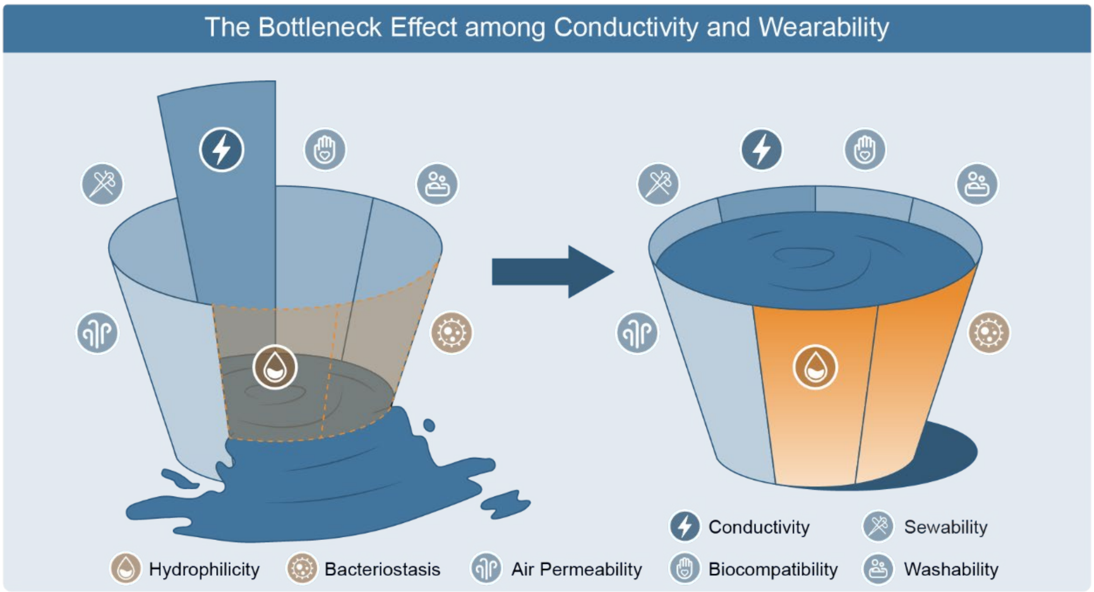

Figure R4 | The bottleneck effect of conductivity and multiple wearability performance.

Reference	Conductivity (S m^{-1})	Sheet Resistance (Ω sq^{-1})	Fabrics/ Fibers	Wearability*
**Our work**	**283.06**	**27.19**	**PP**	√
**Our work****	**1214.84**	**6.58**	**PP**	×
[11]	11	-	PP	×
[12]	35.6	-	PP	×
[13]	6.24	-	PP	×
[14]	-	600	Nylon	×
[15]	10	-	Glass	×
[16]	3.3	-	Polyamide	×
[17]	33.6	-	PEDOT:PSS	×
[18]	57.9	-	Silk	×
[19]	3.4	-	Cotton	×
[20]	3.06×10^{-2}	-	Silk	×
[21]	-	3.69×10^{4}	Cotton	×
[22]	450	-	Nylon	×
[23]	1040	-	Nylon	×
[24]	-	2.14×10^{3}	Cotton	×
[25]	0.09	4.5×10^{4}	Wool	×
[26]	10.9	910	Cotton	×
[27]	-	560	Cotton	×
[28]	241.70	11.92	Poly-Cotton	×
[29]	12.9	110	Cotton	×
[30]	0.53	1.1×10^{4}	PES	×
[31]	-	840	Cotton	×
[32]	0.01	-	Acrylic	×
[33]	1300	-	Kevlar	×
[34]	55	113.64	Cotton	×

[35]	146.8	17.03	Aramid	×

Table R1 | Conductivity and wearability comparison. *Wearability is concluded based on the reported properties in the respective publications. The absence of reported data for any of the 5 key properties (hydrophilicity, air-permeability, washability, bacteriostasis, and biocompatibility) is considered as a lack of demonstrated wearability. **The data is measured by rGO-PMFs-16$_{HI}$.

Reference

8. Ma, Z. *et al.* Permeable superelastic liquid-metal fibre mat enables biocompatible and monolithic stretchable electronics. *Nat. Mater.* **20**, 859–868 (2021).
9. Yin, L. *et al.* A self-sustainable wearable multi-modular E-textile bioenergy microgrid system. *Nat. Commun.* **12**, 1542 (2021).
10. Libanori, A., Chen, G., Zhao, X., Zhou, Y. & Chen, J. Smart textiles for personalized healthcare. *Nat. Electron.* **5**, 142–156 (2022).
11. Hasan, M. M. *et al.* Functionalization of polypropylene nonwoven fabrics using cold plasma (O2) for developing graphene-based wearable sensors. *Sens. Actuators A-Phys.* **300**, 111637 (2019).
12. Pan, Q., Shim, E., Pourdeyhimi, B. & Gao, W. Highly Conductive Polypropylene-Graphene Nonwoven Composite via Interface Engineering. *Langmuir* **33**, 7452–7458 (2017).
13. Sun, T. *et al.* Self-Reinforced Polypropylene/Graphene Composite with Segregated Structures to Achieve Balanced Electrical and Mechanical Properties. *Ind. Eng. Chem. Res.* **59**, 11206–11218 (2020).
14. Neves, A. I. S. *et al.* Towards conductive textiles: coating polymeric fibres with graphene. *Sci. Rep.* **7**, 4250 (2017).
15. Mohan, V. B. & Bhattacharyya, D. Mechanical characterization of functional graphene nanoplatelets coated natural and synthetic fiber yarns using polymeric binders. *Int. J. Smart Nano Mater.* **11**, 78–91 (2020).
16. Zhao, H. *et al.* Fast and facile graphene oxide grafting on hydrophobic polyamide fabric via electrophoretic deposition route. *J. Mater. Sci.* **53**, 9504–9520 (2018).
17. Yang, L., Pan, L., Xiang, H., Fei, X. & Zhu, M. Organic-Inorganic Hybrid Conductive Network to Enhance the Electrical Conductivity of Graphene-Hybridized Polymeric Fibers. *Chem. Mater.* **34**, 2049–2058 (2022).
18. Liang, B. *et al.* Fabrication and application of flexible graphene silk composite film electrodes decorated with spiky Pt nanospheres. *Nanoscale* **6**, 4264–4274 (2014).
19. Hu, X., Tian, M., Qu, L., Zhu, S. & Han, G. Multifunctional cotton fabrics with graphene/polyurethane coatings with far-infrared emission, electrical conductivity, and ultraviolet-blocking properties. *Carbon N. Y.* **95**, 625–633 (2015).
20. Zulan, L. *et al.* Reduced Graphene Oxide Coated Silk Fabrics with Conductive Property for Wearable Electronic Textiles Application. *Adv. Electron. Mater.* **5**, 1800648 (2019).
21. Karim, N. *et al.* Scalable Production of Graphene-Based Wearable E-Textiles. *ACS Nano* **11**, 12266–12275 (2017).
22. Yapici, M. K., Alkhidir, T., Samad, Y. A. & Liao, K. Graphene-clad textile electrodes for electrocardiogram monitoring. *Sens. Actuators B Chem.* **221**, 1469–1474 (2015).
23. Yun, Y. J. *et al.* A Novel Method for Applying Reduced Graphene Oxide Directly to Electronic Textiles from Yarns to Fabrics. *Adv. Mater.* **25**, 5701–5705 (2013).

24. Karim, N. *et al.* All inkjet-printed graphene-based conductive patterns for wearable e-textile applications. *J. Mater. Chem. C Mater.* **5**, 11640–11648 (2017).
25. Javed, K., Galib, C. M. A., Yang, F., Chen, C. M. & Wang, C. A new approach to fabricate graphene electro-conductive networks on natural fibers by ultraviolet curing method. *Synth. Met.* **193**, 41–47 (2014).
26. Zhou, Q., Ye, X., Wan, Z. & Jia, C. A three-dimensional flexible supercapacitor with enhanced performance based on lightweight, conductive graphene-cotton fabric electrode. *J. Power Sources* **296**, 186–196 (2015).
27. Xu, L. L., Guo, M. X., Liu, S. & Bian, S. W. Graphene/cotton composite fabrics as flexible electrode materials for electrochemical capacitors. *RSC Adv.* **5**, 25244–25249 (2015).
28. Afroj, S., Tan, S., Abdelkader, A. M., Novoselov, K. S. & Karim, N. Highly Conductive, Scalable, and Machine Washable Graphene-Based E-Textiles for Multifunctional Wearable Electronic Applications. *Adv. Funct. Mater.* **30**, 2000293 (2020).
29. Abdelkader, A. M. *et al.* Ultraflexible and robust graphene supercapacitors printed on textiles for wearable electronics applications. *2D Mater.* **4**, 035016 (2017).
30. Molina, J. *et al.* Electrochemical characterization of reduced graphene oxide-coated polyester fabrics. *Electrochim. Acta* **93**, 44–52 (2013).
31. Shateri-Khalilabad, M. & Yazdanshenas, M. E. Preparation of superhydrophobic electroconductive graphene-coated cotton cellulose. *Cellulose* **20**, 963–972 (2013).
32. Fugetsu, B., Sano, E., Yu, H., Mori, K. & Tanaka, T. Graphene oxide as dyestuffs for the creation of electrically conductive fabrics. *Carbon N. Y.* **48**, 3340–3345 (2010).
33. Samad, Y. A., Li, Y., Alhassan, S. M. & Liao, K. Non-destroyable graphene cladding on a range of textile and other fibers and fiber mats. *RSC Adv.* **4**, 16935–16938 (2014).
34. Sahito, I. A., Sun, K. C., Arbab, A. A., Qadir, M. B. & Jeong, S. H. Graphene coated cotton fabric as textile structured counter electrode for DSSC. *Electrochim. Acta.* **173**, 164–171 (2015).
35. Guo, Q. *et al.* Structural–Functional Integrated Graphene-Skinned Aramid Fibers for Electromagnetic Interference Shielding. *ACS Nano* **18**, 33566–33575 (2024).

2. Moreover, this value of conductivity was not shown in any materials including Supplementary Information.

Response: Thank you for the kind reminder. We have added numeric notes into Figure 5c for better clarity.

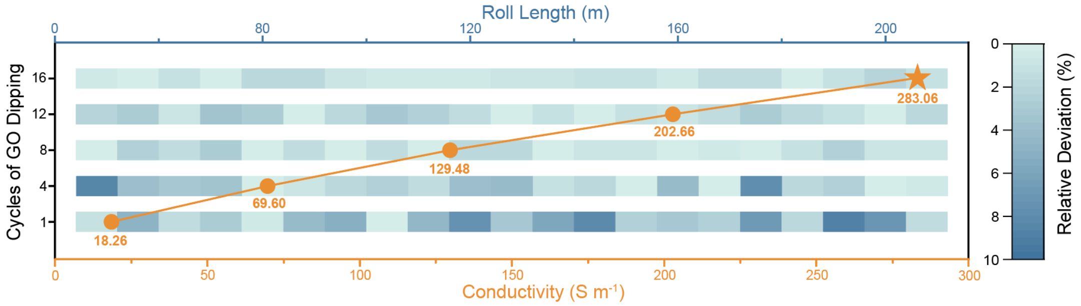

Figure 5c | The conductivity of 200 m roll for multiple rGO-PMFs and their relative deviation.

3. Different swelling times- (Fig. S11), different GO concentrations- (Fig. S12), single dipping time- (Fig. S13), and different reduction times- (Fig. S14) dependent conductivity of rGO-PMF-16 should be provided instead of the conductivity of rGO-PMF-1 and rGO-PMF-4.

Response: Thank you for the valuable comments. We have updated Fig. S15-S18 with conductivity data of rGO-PMF-16 and the corresponding discussion.

a. Different swelling times

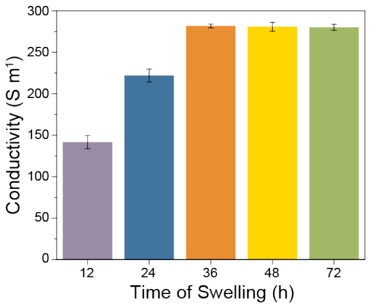

Supplementary Fig. 15 | The conductivity of rGO-PMF-16 under different swelling time.

The influence of swelling time is investigated. As shown in Supplementary Fig. 15, the conductivity of rGO-PMF-16 reaches a plateau after 36 h, indicating that a swelling time of 36 h is sufficient for the process. Therefore, 36 h is selected as the optimal swelling time for all subsequent experiments.

b. Different GO concentrations

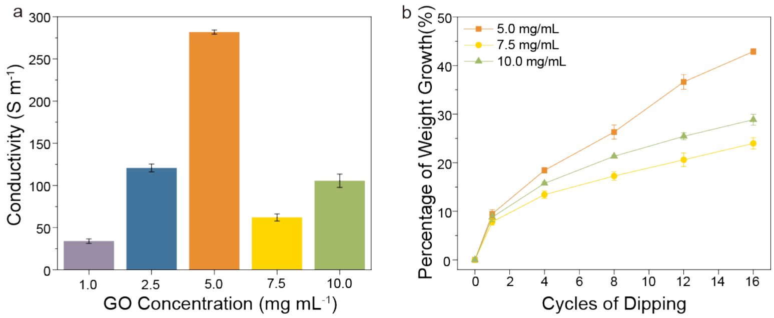

Supplementary Fig. 16 | The conductivity of rGO-PMF-16 under different GO concentration and the weight growth of rGO-PMF-16s under 5.0, 7.5 and 10 mg mL^{-1}. a, The conductivity of rGO-PMF-16 under different GO concentrations. **b,** The GO weight growth of rGO-PMF-16 under 5.0, 7.5 and 10 mg mL^{-1} in dipping process.

As shown in Supplementary Fig. 16a, the conductivity of rGO-PMF-16 depends strongly on GO concentration, reaching a maximum at 5.0 mg mL^{-1}. This optimum

corresponds to an efficient coaxial coating structure, where GO nanosheets uniformly penetrate the fabrics, forming a continuous 3D conductive pathway. At higher concentrations (7.5 and 10 mg mL^{-1}), however, excessive GO limits deep penetration, resulting in lower weight growth (Supplementary Fig. 16b) and inefficient conductive networks. Consequently, both higher concentrations yield inferior conductivity compared to the optimal 5.0 mg mL^{-1} condition with its fully interconnected conformal 3D network.

c. Single dipping time

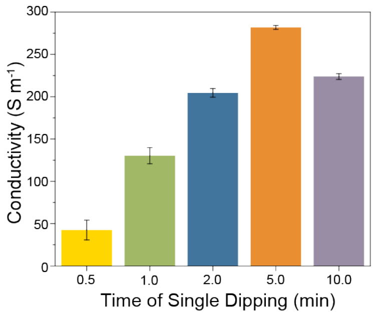

Supplementary Fig. 17 | The conductivity of rGO-PMF-16 under different single dipping time.

The effect of a single dipping cycle is investigated. As presented in Supplementary Fig. 17, rGO-PMF-16 prepared with a 5.0 min dipping time exhibits the highest conductivity. A shorter time may lead to insufficient adsorption, while a longer time might cause nanosheet re-stacking. Therefore, a 5.0 min single dipping time is identified as optimal.

d. Different reduction times

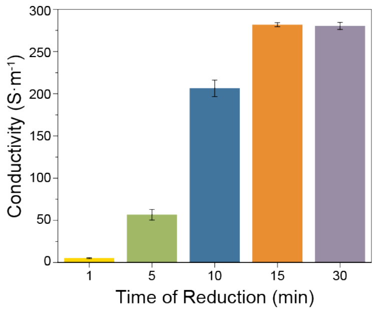

Supplementary Fig. 18 | The conductivity of rGO-PMF-16 under different reduction time.

The effect of chemical reduction time is investigated. As shown in Supplementary Fig. 18, the conductivity of rGO-PMF-16 plateaus after 15 min of reduction, indicating that 15 min is sufficient for effective reduction. Consequently, a reduction time of 15 min is adopted for all samples.

4. The authors wrote that the bridge and bunding structures cause to enhance the conductivity. If then, the highest conductivity of rGO-PMF-16 originates from the large electron pathway due to the 16 dip coating. In this regard, the air permeability of rGO-PMFs (Fig. S17) is not understandable.

Response: Thank you for raising this important point regarding the correlation between conductivity and air permeability. While a straightforward inverse correlation might suggest that the highest conductivity should coincide with the lowest permeability, the microstructure evolution in our coaxial fiber system reveals a more nuanced, three-stage process that fully explains this phenomenon, as evidenced by SEM image analysis (Figure R5, rearranged from Supplementary Fig. 1, and Figure 2e).

Stage 1 (1-4 cycles): Gap Coverage and Initial Bridging

During the initial cycles, rGO nanosheets coat individual fibers and begin forming bridges at fiber intersections. This progressively reduces inter-fiber gaps, leading to continuously decreasing air permeability, and reaching a minimum at 4 cycles.

Stage 2 (4-12 cycles): Fiber Bundling and Gap Re-opening

With further coating (up to 12 cycles), the rGO structures become substantial enough to pull adjacent fibers into bundled assemblies. This bundling consolidates the conductive material at junctions and, importantly, re-opens larger gaps between bundles, causing air permeability to increase from its minimum.

Stage 3 (12-16 cycles): Inter-Bundle Bridging for Optimal Conductivity

At 16 cycles, substantial rGO bridges connect adjacent bundles, establishing a highly conductive network. Although these inter-bundle structures partially occupy some reopened gaps, they form in a spatially discrete manner that preserves significant inter-bundle porosity. This architecture enables maximal electron transport along the bridging pathways while maintaining the highest air permeability. Further dipping would lead to excessive inter-bundle filling, compromising air permeability. Thus, rGO-PMF-16 achieves an optimal trade-off, leveraging bridging for conductivity without sacrificing structural openness.

Unlike conventional sandwich-type conductive composites, where conductivity improves through progressive pore filling and air permeability decreases monotonically, our coaxial system relies on bridging rather than void-filling for electron transport. This unique bundled-and-bridged architecture thereby breaks the conventional trade-off between conductivity and air permeability. It achieves this by decoupling the governing pathways: robust inter-bundle rGO bridges establish highly efficient electron transport for maximum conductivity, while the concurrently preserved inter-bundle porosity maintains high air permeability.

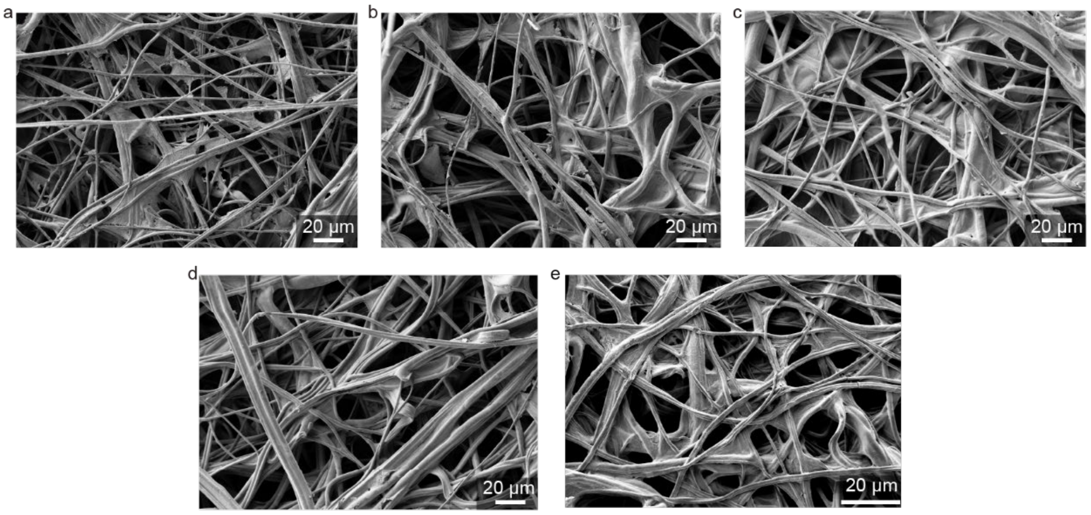

Figure R5 | The SEM images of rGO-PMFs in different cycles of dipping. a-e, The SEM image of rGO-PMF-x. x represent 1, 4, 8, 12, 16 cycles of dipping respectively.

5. In the same way, the highest conductivity should be shown in rGO-PMF fabricated large size of GO (1016.29 nm) but not 401.53 nm of GO (Figs. S6 and S8). A profound physical interpretation should be provided.

Response: We sincerely thank the reviewer for this insightful comment, which prompts a deeper examination of the relationship between GO nanosheet size and conductivity in rGO–PMFs. In response to this observation, we systematically investigate the conductivity over 1 to 16 dipping cycles to comprehensively capture the evolving architecture of the conductive network, rather than relying on a single data point. As rightly noted, samples synthesized with larger nanosheets should, in principle, exhibit higher conductivity. However, our experimental results (Figure R6a) reveal a non-monotonic trend in conductivity, indicating that macroscopic electrical performance is

governed not only by the intrinsic properties of individual nanosheets but also by the architecture of the conductive network within the fabrics. The observed behavior can be interpreted as a trade-off between a penetration-limited regime (large GO) to a junction resistance-limited regime (small GO):

Large GO nanosheets (1016.29 nm and 667.56 nm): These sheets show limited penetration into PMFs, as evidenced by their low GO weight growth during dipping (Figure R6b). They form a sandwich structure rather than a continuous 3D coaxial network, resulting in low conductivity despite high intrinsic in-plane conductivity.

Intermediate GO nanosheets (506.30 nm, 401.53 nm): This range represents an optimal balance. The nanosheets penetrate effectively (shown by a sharp increase in GO weight growth) and coaxially coat the fibers, establishing a robust, long-range conductive network. The peak conductivity at 401.53 nm reflects this optimal regime.

Small GO nanosheets (329.12 nm): While achieving the highest loading and penetration, the conductive network formed by these small sheets contains an excessively high density of inter-nanosheet junctions. This introduces significant electron junction resistance[36,37], which dominates and leads to a decrease in overall conductivity.

In summary, the conductivity of rGO-PMFs is also controlled dominantly by the architecture of the conductive network. The non-monotonic trend results from a transition from a penetration-limited regime (large GO) to a junction resistance-limited regime (small GO), with an optimal intermediate size. This discussion has been added to the revised Supplementary Fig. 6.

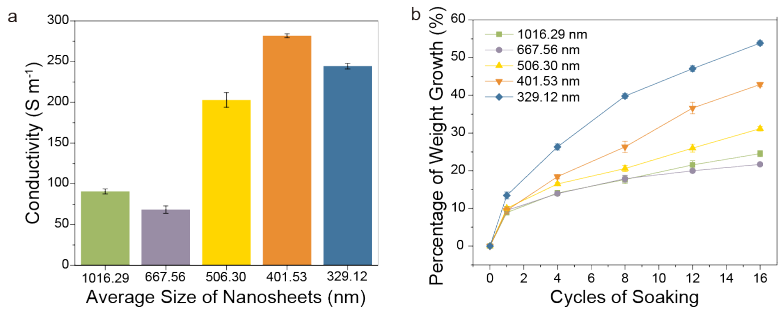

Figure R6 | The conductivity of multiple rGO-PMF-16s in different average sizes of GO nanosheets and their GO weight growth. a, The conductivity of rGO-PMF-16s in different average sizes of GO nanosheets. **b,** The GO weight growth of rGO-PMF-16s under different average

sizes of GO nanosheets in dipping process.

Reference

36. Gabbett, C. *et al.* Understanding how junction resistances impact the conduction mechanism in nano-networks. *Nat. Commun.* **15**, 4517 (2024).

37. Kelly, A. G., O'Suilleabhain, D., Gabbett, C. & Coleman, J. N. The electrical conductivity of solution-processed nanosheet networks. *Nat. Rev. Mater.* **7**, 217–234 (2022).

6. The rGO is the main factor to endow the electrical conductivity of rGO-PMFs. If then, what is the reason for the different conductivity of various polymers shown in Fig. 4?

Response: We thank the reviewer for their insightful comment. While rGO is indeed the conductive component, our results demonstrate that the microstructure of the fabric scaffold is a critical determinant for the efficiency of electron transport. We quantify this microstructural influence by introducing the fiber network volume fraction (φ), which represents the volumetric density of the fiber network (Table R2). Our analysis reveals a strong positive correlation between φ and conductivity (Figure R7). A higher φ signifies a denser, more interconnected scaffold, which facilitates the formation of a continuous, long-range coaxial rGO conductive pathway. Consequently, while the intrinsic properties of rGO set the fundamental conductivity ceiling, the solid fraction of the underlying polymer fabric is also a key modulating factor for the realized conductivity in the rGO-PMFs. The construction of an efficient conductive rGO network is fundamentally governed by the solid fraction of the underlying polymer fabric scaffold. The corresponding results are also included in the revised Supplementary Fig. 14 and Supplementary Table 5.

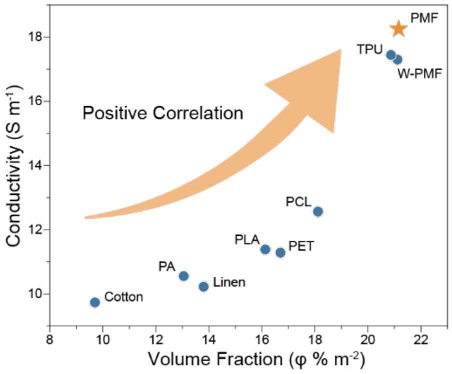

Figure R7 | The conductivity and volume fraction (φ) of each commercial polymer fabrics.

Polymer/ Natural Fabrics	Conductivity ($S\ m^{-1}$)	Intrinsic Density ($g\ cm^{-3}$)	Weight ($g\ m^{-2}$)	Thickness (μm)	Volume Fraction (φ % m^{-2})
PMF	18.26	0.91	25.03	130.01	21.16
W-PMF	17.30	0.91	24.99	130.06	21.11
PCL	12.56	1.15	25.00	120.00	18.12
PLA	11.38	1.24	30.00	149.97	16.13
PA	10.55	1.15	30.03	200.07	13.05
PET	11.28	1.40	35.07	150.00	16.70
TPU	17.44	1.20	50.09	199.98	20.87
Cotton	9.73	1.54	50.10	335.04	9.71
Linen	10.22	1.55	59.92	280.09	13.80

Table R2 | Conductivity, intrinsic density, weight, thickness and volume occupation of each commercial polymer fabrics. The fiber network volume fraction (φ) is calculated as: φ = (Weight / Intrinsic Density) / Thickness. This represents the volume fraction of solid polymer fibers within the total volume of the fabric, providing a quantitative measure of the network's denseness and available scaffold for rGO coating.

7. 1214.84 S/m of conductivity was achieved using HI acid but it had low hydrophilicity. How about air permeability, EMI shielding, bacteriostasis, and biocompatibility of this sample? Occasionally, hydrophobicity is more important than hydrophilicity.

Response: Thanks for your detailed comments. We have provided the performance of air permeability, EMI shielding, bacteriostasis, and biocompatibility of rGO-PMF-16_{HI} as requested.

a. The performance of air permeability

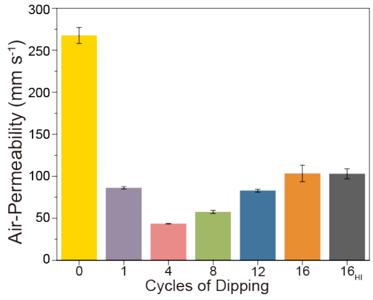

Supplementary Fig. 23 | The air permeability of multiple rGO-PMFs.

We have added the response of comment 5 to this figure for better clarity.

b. The performance of EMI shielding

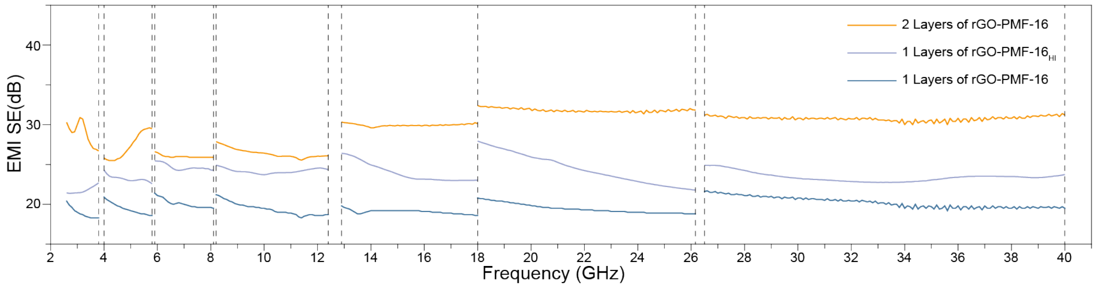

Fig. R8 | The EMI SE of rGO-PMF-16 and rGO-PMF-16$_{HI}$ in 1 and 2 layers from 2.6-40.0 GHz.

The Electromagnetic Interference Shielding Effectiveness (EMI SE) of rGO-PMF-16$_{HI}$ is measured across the 2.6–40.0 GHz. As presented in Figure R8, 1 layer of rGO-PMF-16$_{HI}$ exhibits an average EMI SE of approximately 25 dB, representing an improvement of ~5 dB over rGO-PMF-16. This enhancement, however, is modest relative to the fourfold increase in electrical conductivity and remains inferior to the shielding achieved by simply adding a second layer of rGO-PMF-16. These results underscore a **diminishing return in EMI SE performance with escalating conductivity**. Consequently, the pursuit of ultrahigh conductivity—often at the expense of other wearable properties—yields limited functional gains in shielding performance. The corresponding results are also included in the revised Supplementary Fig. 28.

c. The performance of bacteriostasis

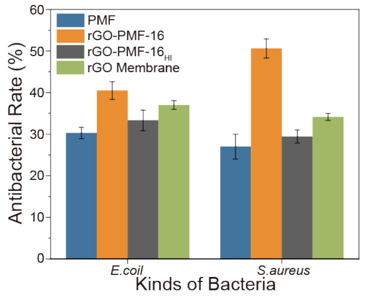

Supplementary Fig. 25 | The antibacterial rate of PMF, rGO-PMF-16 rGO-PMF-16HI and rGO membrane for *Escherichia coli* (*E.coil*) and *Staphylococcus aureus* (*S.aureus*).

According to the data in Supplementary Fig.25, rGO-PMF-16 obtains the highest antibacterial rate among all the 4 samples. However, the performance of rGO-PMF-

16$_{HI}$ is low, near the plain PMF itself. It is because the high reduction eliminates most oxygen-containing functional groups and defects, which leads to the lack of glutathione oxidation effect. High reduction brings obvious hydrophobicity and causes less bacteria to attach to the surface leading to deteriorating the nano knife effect. Moreover, the rGO membrane's performance of bacteriostasis shows the effect of rGO itself. rGO-PMF-16 exhibits the promotion of bacteriostasis performance by coaxial structure's large surface area.

d. The performance of biocompatibility

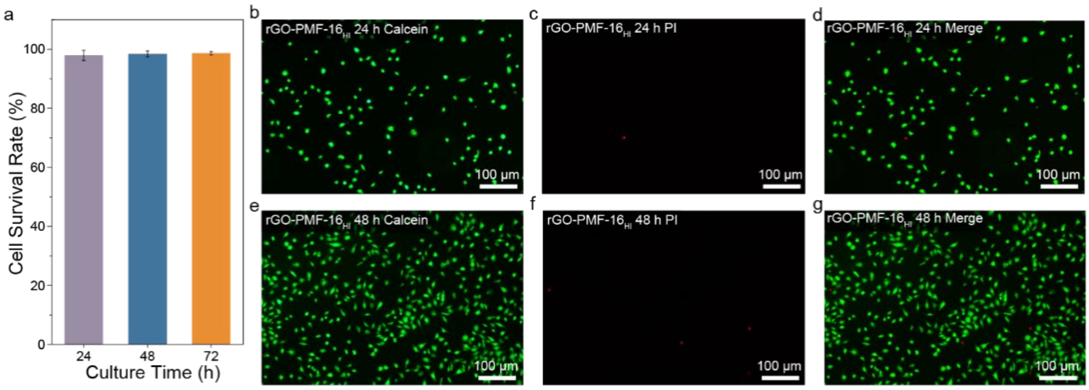

Fig. R9 | The cell survival rate of rGO-PMF-16$_{HI}$ and the fluorescence microscopy image of human fibroblasts (HFB) cultivated with rGO-PMF-16$_{HI}$**. a,** The cell survival rate of rGO-PMF-16$_{HI}$. The calcein fluorescence microscopy image of HFB cultivated with r rGO-PMF-16$_{HI}$ in **b)** 24 and **e)** 48h. The propidium iodine (PI) fluorescence microscopy image of HFB cultivated with rGO-PMF-16$_{HI}$ in **c)** 24 and **f)** 48h. The merge fluorescence microscopy image of HFB cultivated with rGO-PMF-16$_{HI}$ **d)** 24 and **g)** 48h.

The biocompatibility performance of rGO-PMF-16$_{HI}$ is measured. As presented in Figure R9, rGO-PMF-16$_{HI}$ shows excellent biocompatibility as rGO-PMF-16. The figure and corresponding results are also included in the revised Supplementary Fig. 27. Moreover, as highlighted in recent studies published for wearable devices interfaced with the skin[38,39], hydrophilicity is crucial for ensuring long-term wear comfort and skin health, and is itself an important dimension of wearability evaluation. Our material design adheres to this principle, aiming to maintain the integrity and health of the skin barrier.

Therefore, while the pursuit of extreme electrical conductivity remains a valid pathway for specific applications, it often necessitates trade-offs that compromise other essential wearable properties, such as hydrophilicity and bacteriostasis. This is exemplified by

the marginal improvement in EMI shielding (~5 dB) despite a fourfold increase in conductivity, highlighting a law of diminishing returns. Our work demonstrates that **a holistic evaluation of e-fabrics must extend beyond a single metric like conductivity**. The innovation of our temporal decoupling strategy lies in its ability to fabricate multi-functional fabrics that maintain an optimal and practical balance between respectable conductivity and a full suite of wearable comforts and safety features, thereby advancing the field towards realistically deployable and user-centric electronic fabrics.

Reference

38. Liang, X. *et al.* Hydrophilic, Breathable, and Washable Graphene Decorated Textile Assisted by Silk Sericin for Integrated Multimodal Smart Wearables. *Adv. Funct. Mater.* **32**, 2200162 (2022).

39. Ma, X. *et al.* A monolithically integrated in-textile wristband for wireless epidermal biosensing. *Sci. Adv.* **9**, eadj2763 (2023).

8. The reduction methods cause different conductivities as mentioned in comment 7. Hence the rGO is the main factor but not microscopic morphology as the authors mentioned on page 20 of Supplementary Information.

Response: Thank you for the insightful review. The different reduction methods lead to varying conductivity. While the intrinsic properties of rGO set the fundamental conductivity ceiling, the construction of an efficient conductive rGO network in fabrics is also a key modulating factor for the realized conductivity in the rGO-PMFs. We have deleted inappropriate statements and updated the discussion from response 5.

9. The antibacterial rate of rGO-PMF-16 is higher than that of PMF. Inserting the data of rGO itself helps the reader understand the role of rGO.

Response: We have updated the data and the figure as follows:

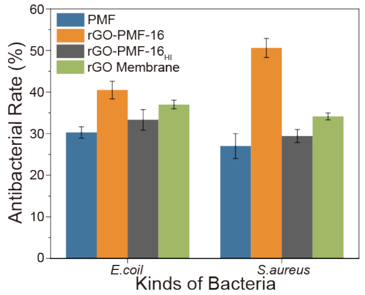

Supplementary Fig. 25 | The antibacterial rate of PMF, rGO-PMF-16 rGO-PMF-16HI and rGO membrane for *Escherichia coli* (*E.coil*) and *Staphylococcus aureus* (*S.aureus*).

We have updated the discussion of this figure in response 7.

In this light, there is no novelty for the publication in Nature Communications.

Response: We thank the reviewer for the critique and the opportunity to underscore the foundational novelty of our work. Our manuscript introduces a temporal decoupling (TD) strategy that establishes a new paradigm for fabricating e-textiles, fundamentally addressing the long-standing dilemma between the deep penetration and robust adhesion of conductive macromolecules. This core innovation is not an incremental improvement but a conceptual and methodological leap, which we have mechanistically elucidated and scaled to demonstrate its unique ability to integrate high conductivity with comprehensive wearability—a combination previously unattained. We are confident that these contributions represent a significant advance worthy of ***Nature Communications***.

The key innovations of our work are:

1. **A Universal Coating Mechanism Enabling Balanced Performance:**

The core innovation is the TD strategy's ability to dynamically control interfacial interactions during fabrication. This is mechanistically elucidated through multi-scale evidence—including molecular dynamics simulations and XPS depth profiling—which confirms the programmed sequence of weak (for penetration) and strong (for adhesion) interactions. This orchestrated process drives a unique structural evolution, resulting in a **conformal, coaxial rGO morphology** across diverse ordinary fabrics (synthetic and natural). It is this specific, designed architecture that uniquely enables the simultaneous

achievement of high conductivity and essential wearability properties (air permeability, washability, etc.), effectively breaking the longstanding performance trade-off.

2. Scalable Manufacturing of a High-Performance Platform:

The true test of a methodology's novelty and impact is its scalability and real-world applicability. Our strategy is engineered for mass production, successfully demonstrating unprecedented consistency (283.06 S m^{-1} with <2.4% deviation over 200 meters) at an ultralow cost (\$0.38 m^{-2}). This scalable process directly yields a robust, sewable fabric platform that validates its practical utility through high-performance applications like EMI shielding and uniform Joule heating, meeting commercial standards. This combination of massive, low-cost production with holistic, device-ready performance represents a significant leap towards the industrial adoption of graphene-based E-fabrics.

In summary, our work provides a new mechanistic framework and a scalable manufacturing pathway for creating multi-functional e-textiles, moving beyond the limitations of previous approaches. We believe these fundamental and practical advances are fully aligned with the innovative standards of ***Nature Communications***.

Reviewer #3 (Remarks to the Author):

General Comments for Main Text:

1. **Clarification on Triton Type**: The study mentions the use of Triton, but there are various types within the Triton family. Please specify which type of Triton was used and provide a rationale for its selection.

Response: Thank you for this insightful question. As indicated on Line 67 of the manuscript, we use Triton X-100 (hereafter referred to as 'Triton' for simplicity). The selection of Triton is based on the Hydrophile-Lipophile Balance (HLB) value, which is a critical parameter for surfactant efficacy in hydrophilic modification. As discussed in prior work[40], an HLB value that is too low results in insufficient hydrophilicity, while a value that is too high compromises the surfactant's affinity for the polypropylene (PP) matrix. The optimal HLB range has been identified as 11.9 to 14.8. Accordingly, we select Triton X-100, which has an HLB value of 13.5, falling squarely within this optimal range, as compared to other Triton variants (Table R3). This discussion has been added to the revised Supplementary Table 3.

Triton Type	HLB Value
Triton X-15	9.5
Triton X-45	10.4
**Triton X-100**	**13.5**
Triton X-165	15.8
Triton X-305	17.3

Table R3 | Different HLB values of Triton series. HLB stands for Hydrophile-Lipophile Balance.

Reference

40. Guo, H., Huang, J., Ye, Y., Huang, J. & Wang, X. Surface modification of polypropylene by the entrapping method using the short-chained stearyl-alcohol poly(ethylene oxide) ether modifier. *J. Appl. Polym. Sci.* **133**, 43607 (2016).

2. **Justification for Choosing Triton**: While Triton is a well-known non-ionic surfactant, there are many other non-ionic surfactants available. Could you elaborate on why Triton was chosen over other alternatives?

Response: Thank you for this insightful comment. In our systematic screening (Fig. 3a, b), we have evaluated several non-ionic surfactants, including Triton X-100, Trideceth-4 and BNO-12. Triton X-100 is uniquely effective as a non-ionic surfactant by circumventing detrimental electrostatic effects. Moreover, its triphilic structure—comprising an aliphatic segment for anchoring into the PP fiber, a polar PEG chain facilitating GO penetration, and an aromatic group enabling post-reduction stabilization via π–π stacking—provided precise control over interfacial interactions. This molecular architecture was essential to temporally decoupled strategy, allowing simultaneous deep penetration and the formation of a robust, conformal coating.

Moreover, to proactively demonstrate the universal applicability of our strategy, a synthesis is successfully replicated using cardanol polyoxyethylene ether (CPE-9), with triphilic structure. The resulting fabrics (rGO-cPMF-x) exhibit a conformal rGO coaxial coating (Figure R2a and b) and achieve high conductivity (up to 153.87 S m^{-1}, Figure R2c). These results unequivocally prove that our strategy is universally adaptable and provides another pathway for developing high-performance conductive fabrics. Figure R2 and corresponding discussion have been added to the Supporting Information (Supplementary Fig. 11).

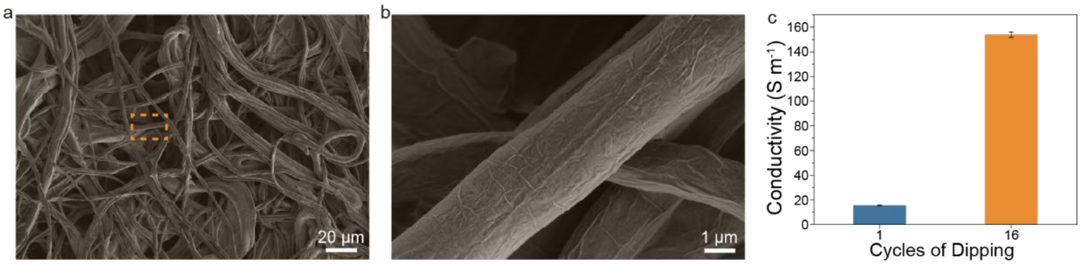

Figure R2 | The synthesis results of rGO-cPMFs. a, The SEM image of rGO-cPMF-16's surface. **b,** The SEM image of marked part in figure R2a. **c,** The conductivity of rGO-cPMF-1 and rGO-cPMF-16.

3. **Experimental Design**: The current study utilized fabric directly for coating. To strengthen the evidence for the mechanisms proposed by the authors, it is recommended to perform additional experiments using single fibers with larger diameters. This approach could provide more detailed insights into the interactions and validate the proposed mechanisms more effectively.

Response: We thank the reviewer for their valuable suggestion. While experiments on single fibers could provide further insights, the core objective of this study was to achieve ultrahigh depth resolution for accurately profiling chemical gradients across ultrathin interfaces—below 25 nm, as confirmed by HRTEM (Fig. 2k, l). The curved surface of a single fiber, even with a larger diameter, would introduce non-uniform etching during Ar-GCIB sputtering and uneven X-ray signal distribution, severely compromising depth resolution and obscuring the nanoscale chemical changes we aim to resolve. Therefore, to ensure reliable and high-resolution depth profiling, we utilized a hot-pressed PMF membrane subjected to the same synthesis process as rGO-PMF. This flat, homogeneous geometry is essential for obtaining convincing XPS depth profiles that accurately validate the proposed mechanisms.

4. **Reallocation of Data**: The main text currently consists mainly of pictures and photos, with too many data and results placed in the Supplementary Information (SI). Key results from the SI should be re-allocated back into the main text. For example, Figure 6 contains almost no data and is filled with photos/ pictures. Important data should be included in the main text to enhance the clarity and impact of the study.
Response: We thank the reviewer for this comment. Figure 6 is designed to holistically showcase our material's multiple functionalities as a wearable platform: Figure 6i and 6k-m present the quantitative core performance (EMI shielding, Joule heating), while Figure 6a-g provide essential qualitative demonstrations of key wearability attributes (e.g., hydrophilicity, biocompatibility). We find this structure effectively balances quantitative rigor with visual proof of practical application. To strengthen the manuscript, we have now incorporated key quantitative metrics supporting these wearability tests (e.g., contact angle, antibacterial rate) directly into the main text, ensuring all critical data is prominently featured without compromising the figure's clarity and impact.

5. **Conductivity Calculation**: How was the conductivity calculation performed in this study? Additionally, how does the way the conductivity is deduced compare to

other literature? Please provide an explanation and cross-comparison.

Response: Thank you for the insightful comment. The electrical conductivity was calculated based on the sheet resistance (measured using a four-point probe system) and the thickness of the rGO-PMFs. This approach ensures that the sheet resistance value is geometry-independent, being governed solely by the intrinsic material properties and sample thickness. By incorporating the total thickness into the conductivity calculation ($\sigma = 1/(R_{sq} \cdot t)$), we effectively account for the conductive pathways along the out-of-plane (z-) direction, thereby providing a more accurate representation of the overall conductivity in our three-dimensional porous coaxial structure.

This methodology aligns with well-established practices for thin film and conductive fabric characterization, as reflected in prior studies[8,23,35]. In contrast, a few reports on graphene-based fabrics, especially those with sandwich structures, often report only sheet resistance or in-plane conductivity, overlooking the contribution of through-thickness conduction[28]. Given the porous and volumetrically conductive nature of our rGO-PMFs, the conventional in-plane approximation would underestimate the actual charge transport capability. This approach is thus a more complete and physically relevant conductivity assessment.

Reference

8. Ma, Z. *et al.* Permeable superelastic liquid-metal fibre mat enables biocompatible and monolithic stretchable electronics. *Nat. Mater.* **20**, 859–868 (2021).

23. Yun, Y. J. *et al.* A Novel Method for Applying Reduced Graphene Oxide Directly to Electronic Textiles from Yarns to Fabrics. *Adv. Mater.* **25**, 5701–5705 (2013).

28. Afroj, S., Tan, S., Abdelkader, A. M., Novoselov, K. S. & Karim, N. Highly Conductive, Scalable, and Machine Washable Graphene-Based E-Textiles for Multifunctional Wearable Electronic Applications. *Adv. Funct. Mater.* **30**, 2000293 (2020).

35. Guo, Q. *et al.* Structural–Functional Integrated Graphene-Skinned Aramid Fibers for Electromagnetic Interference Shielding. *ACS Nano* **18**, 33566–33575 (2024).

6. **Conversion of Costs**: In Figure 5, how are energy costs and carbon costs converted to 'production cost in S.m^-2'? Please clarify the methodology used for these conversions.

Response: Thank you for the detailed review. The conversion of energy and carbon

costs to a per square meter production cost (US$ m^{-2}) in Figure 5 is direct and based on the data presented in Supplementary Tables 7 and 8.

Energy Cost (US$ m^{-2}): This is calculated by multiplying the total energy usage for each process (kWh·m^{-2}) from Supplementary Table 8 by the local electricity price (US$ 0.11 per kWh), as shown in the "Energy Cost" section of Supplementary Table 7.

Carbon Cost (US$·m^{-2}): This is calculated by first determining the total CO_2 emissions (kg CO_2·m^{-2}) from Supplementary Table 8 and then by multiplying this value by the applied carbon price (US$ 13.28 per kg of CO_2), as shown in the "Carbon Cost" row of Supplementary Table 7.

7. **Variation in Sheet Resistance**: It is important to state the batch-to-batch variation and position-to-position variation in sheet resistance. This information is crucial for understanding the consistency and reliability of the results.

Response: We have evaluated the batch-to-batch and position-to-position variation systematically. For our continuously produced 200-meter roll, the sheet resistance was measured every 20 meters (longitudinal variation) at three transverse positions (center and 5 cm from each edge) to assess the position-to-position variation. The calculated conductivity from all these data points yielded an average of 283.06 S m^{-1} with a standard deviation of less than 2.4%.

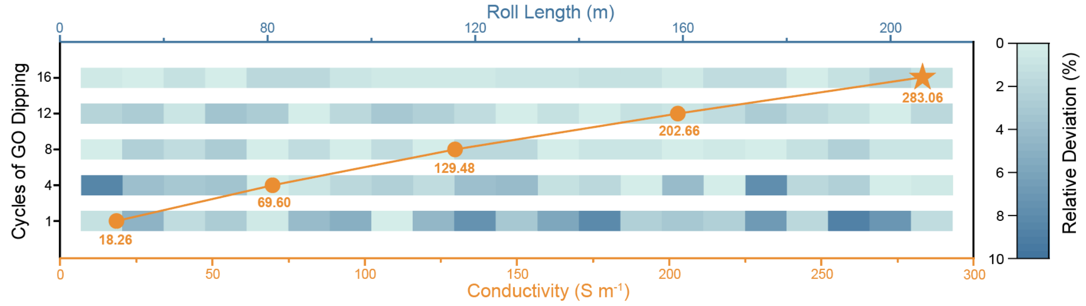

Figure 5c | The conductivity of 200 m roll for multiple rGO-PMFs and their relative deviation.

Comments for Supplementary Information (SI):

1. **Surface Chemical Characterizations**: Perform surface chemical characterizations for the commercial polymer fabrics, as many of these fabrics could have pre-existing surface treatments that significantly affect the functionalization

mechanisms and outcomes.

Response: Thank you for this insightful comment. In response, we have conducted systematic XPS characterization on the as-received commercial polymer fabrics. The results reveal no detectable evidence of pre-existing surface treatments across multiple common fabric types (Figure. R10). Figure R10 and corresponding discussion have been added to the Supporting Information (Supplementary Fig. 12).

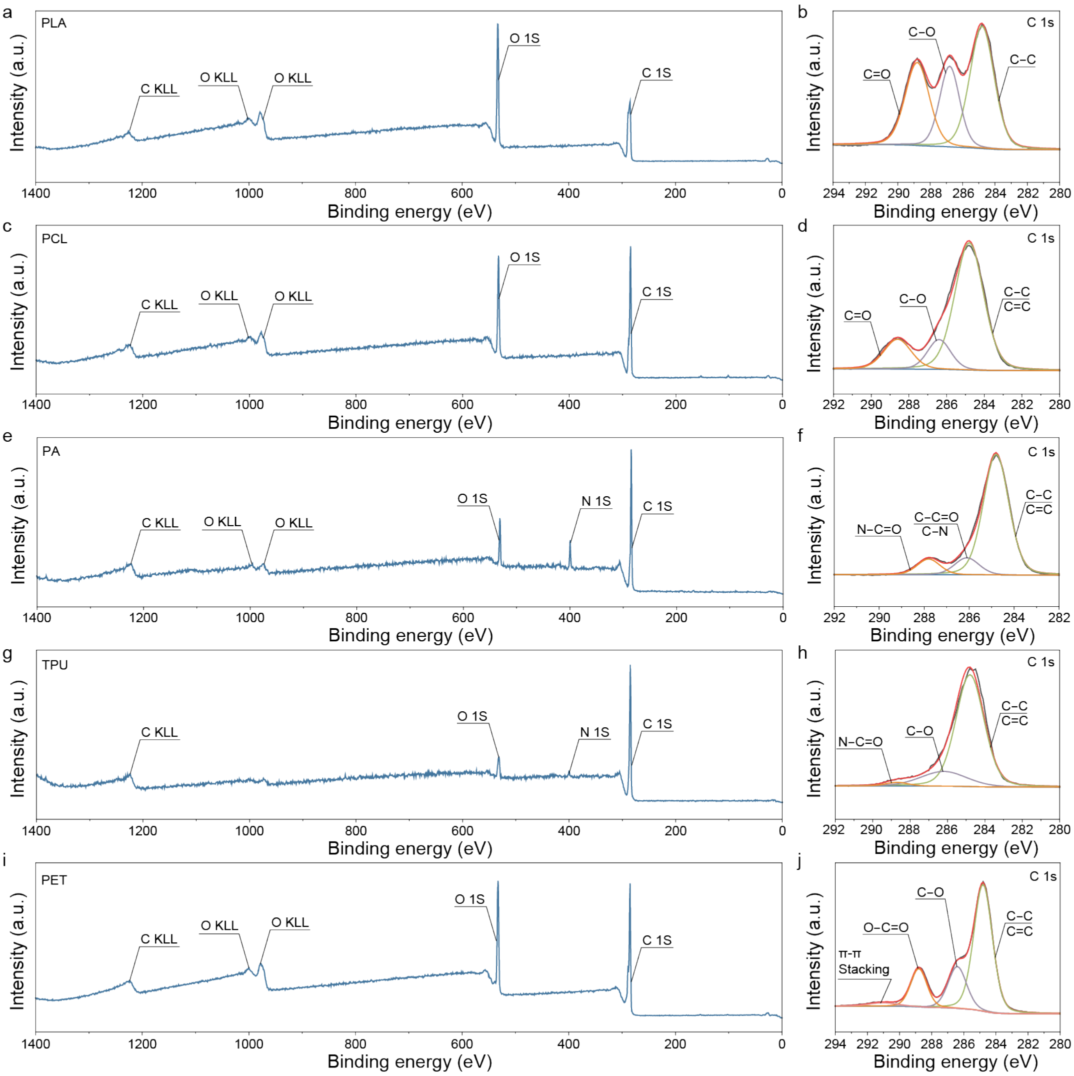

Figure R10 | XPS spectra of the untreated commercial fabrics. a-b, Full spectrum and high-resolution C 1s spectrum of PLA**. c-d,** Full spectrum and high-resolution C 1s spectrum of PCL**. e-f,** Full spectrum and high-resolution C 1s spectrum of PA**. g-h,** Full spectrum and high-resolution C 1s spectrum of TPU**. i-j,** Full spectrum and high-resolution C 1s spectrum of PET.

2. **Nanoparticle Release**: Check the nanoparticle release from the bath during machine washing. This is important for assessing the environmental impact and safety of the materials used.

Response: Thank you for highlighting this essential aspect of nanoparticle release, which is critical for evaluating the environmental impact and safety of the material. We have directly addressed this issue by monitoring the total weight loss and total conductivity loss of the rGO-PMF during machine washing. rGO-PMF-16 sample exhibits excellent robustness, with only ~2.5% total weight loss and ~4% total conductivity loss after the first washing cycle (Figure. R11). Importantly, these values plateau over the next nine washing cycles, showing no further notable reduction. This minimal and self-limiting loss indicates that nanoparticle release is negligible and confined primarily to the initial removal of loosely adhered residues, with no persistent shedding of the functional rGO coating.

We therefore conclude that the release of rGO nanoparticles from rGO-PMF-16 is minimal, self-limiting, and non-persistent—a release profile that substantially mitigates potential environmental concerns and underscores the material's safety and durability in practical applications. We have added Figure. R11 and the corresponding discussion as Supplementary Fig. 24.

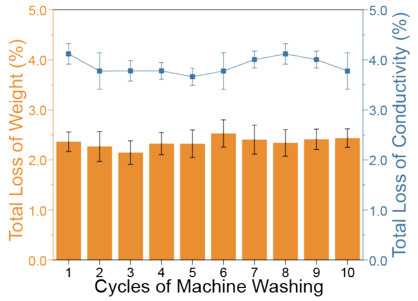

Figure. R11 | The washability of rGO-PMF-16.

3. **Biocompatibility Testing**: Biocompatibility tests should be performed on macrophages instead of fibroblasts. Macrophages are more relevant for assessing the immune response and biocompatibility of the materials.

Response: Thank you for the insightful review. We agree that macrophages are a key cell type for evaluating the immune response to biomaterials, particularly for implantable devices. However, for our specific application of wearable **E-fabrics designed for topical skin contact**, the primary and initial biological interaction occurs with the skin's outer layers. In this context, **human dermal fibroblasts are the most**

relevant and scientifically justified cell model. They are the predominant cell type in the dermis and the primary frontline cells that would interact with our material. Our testing strategy aligns with the ISO 10993-5:2009 standard, which recommends the use of mammalian fibroblast lines for initial cytotoxicity screening of devices with skin contact[41]. Besides, fibroblasts are widely used to evaluate the biocompatibility among recent works about wearable devices[42–44].

Therefore, we believe that assessing cytotoxicity on fibroblasts provides a direct and relevant safety assessment for the intended use of our material. Nonetheless, we appreciate the reviewer's insightful suggestion and will certainly consider incorporating macrophage-based assays in future studies to explore immune responses for more advanced applications.

Reference

41. Gruber, S. & Nickel, A. Toxic or not toxic? The specifications of the standard ISO 10993-5 are not explicit enough to yield comparable results in the cytotoxicity assessment of an identical medical device. *Front. Med. Technol.* **5**, 1195529 (2023).

42. Ye, G., Song, D., Song, J., Zhao, Y. & Liu, N. A Fully Biodegradable and Biocompatible Ionotronic Skin for Transient Electronics. *Adv. Funct. Mater.* **33**, 2303990 (2023).

43. Chen, S. *et al.* Mechanically and biologically skin-like elastomers for bio-integrated electronics. *Nat. Commun.* **11**, 1–8 (2020).

44. Cheng, S. *et al.* Ultrathin Hydrogel Films toward Breathable Skin-Integrated Electronics. *Adv. Mater.* **35**, 2206793 (2023).

4. **Impact of Environmental Conditions**: How do the data differ when operating under different humidity and temperature conditions? Provide an analysis of the impact of these environmental factors on the results.

Response: Thank you for the valuable comment regarding the impact of environmental conditions. We have conducted additional experiments to evaluate the synthesis under different humidity and temperature conditions, simulating real-world scenarios: Winter-like condition: 4 °C and 30% RH and Summer-like condition: 40 °C and 90% RH.

The results are summarized in Fig. R12. We observe that the conductivity under low-temperature conditions (4 °C, 264.03 S m^{-1}) is lower than that at room temperature (25 °C, 283.18 S m^{-1}), primarily due to slowed swelling and surface functionalization. In contrast, the conductivity under high-temperature conditions (40 °C, 287.13 S m^{-1})

is only 1.44% higher than that at room temperature (283.18 S m^{-1} at 25 °C, 50% RH), indicating that the swelling process is sufficient to achieve optimal functionalization. Notably, relative humidity showed negligible influence on conductivity, as the process is based on a wet-chemical route involving sequential soaking in EA and DI-water-based solutions. Although higher temperatures slightly enhance swelling kinetics, they also increase energy consumption and safety risks due to the use of EA. Therefore, we recommend room temperature (25 °C, 50% RH) as the optimal condition for balancing performance, cost, and safety. Figure R12 and corresponding discussion have been added to the Supporting Information (Supplementary Fig. 19).

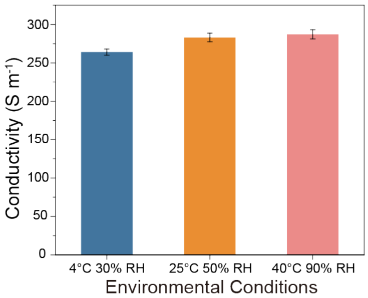

Fig. R12 | The Conductivity of rGO-PMF-16 in different conditions.

5. **Raw Data for Fabric Weights**: In SI-Figure 18, the loss of weight is reported to be between 0 to 2%. Please provide the raw data on the fabric weights that derive these results for transparency and validation.

Response: Thank you for the detailed review. We have listed revised Supplementary Fig.24 and its raw data (Table R4 and 5).

Dipping Cycles	Pre-Washed	Post-washed	Loss (%)	Pre-Washed	Post-washed	Loss (%)	Pre-Washed	Post-washed	Loss (%)	Mean (%)	Standard Deviation
	Sample Set 1			Sample Set 2			Sample Set 3				
Total Weight (g)											
1	49.60	49.50	0.20	49.20	49.10	0.20	51.70	51.60	0.19	0.20	0.0053
4	55.60	54.40	2.16	60.70	59.30	2.31	55.00	53.80	2.18	2.22	0.0796
8	59.70	58.70	1.68	58.80	57.70	1.87	55.20	54.40	1.45	1.67	0.2109
12	65.10	63.80	2.00	61.80	60.70	1.78	57.90	56.70	2.07	1.95	0.1519
16	62.00	60.70	2.10	61.90	60.60	2.10	62.20	60.80	2.25	2.15	0.0880
Total Conductivity (S m^{-1})											
1	32.66	25.79	21.02	42.07	27.65	34.28	33.67	24.64	26.83	27.38	6.6435
4	112.29	102.44	8.77	116.74	108.46	7.09	73.92	67.18	9.11	8.32	1.0843
8	158.34	153.60	2.99	160.47	154.32	3.83	153.69	148.78	3.19	3.34	0.4377
12	221.02	212.51	3.85	212.41	206.23	2.89	188.45	182.12	3.36	3.37	0.4815
16	276.92	268.03	3.21	263.44	254.53	3.38	293.93	283.17	3.66	3.42	0.2272

Table R4 | Raw data of Supplementary Fig. 24a.

Washing Cycles	0	1	2	3	4	5	6	7	8	9	10
						Total Weight (g)					
Sample 1	72.03	70.49	70.45	70.33	70.4	70.53	70.39	70.47	70.37	70.21	70.28
Loss (%)	0.00	2.14	2.19	2.36	2.26	2.08	2.28	2.17	2.30	2.53	2.43
Sample 2	81.29	79.26	79.18	79.75	79.55	79.45	79	79.4	79.16	79.52	79.46
Loss (%)	0.00	2.50	2.60	1.89	2.14	2.26	2.82	2.33	2.62	2.18	2.25
Sample 3	75.5	73.65	73.98	73.85	73.56	73.52	73.62	73.44	73.92	73.59	73.52
Loss (%)	0.00	2.45	2.01	2.19	2.57	2.62	2.49	2.73	2.09	2.53	2.62
Mean (%)	0.00	2.36	2.27	2.15	2.32	2.32	2.53	2.41	2.34	2.41	2.43
Standard Deviation	0.0000	0.1953	0.2982	0.2352	0.2210	0.2749	0.2721	0.2901	0.2655	0.2026	0.1857
						Total Conductivity (S m^{-1})					
Sample 1	282.81	271.81	272.78	272.78	271.81	272.78	272.78	271.81	271.81	271.81	272.78
Loss (%)	0.00	3.89	3.55	3.55	3.89	3.55	3.55	3.89	3.89	3.89	3.55
Sample 2	280.74	268.96	268.96	269.91	269.91	269.91	268.96	268.96	268.96	268.96	268.96
Loss (%)	0.00	4.20	4.20	3.86	3.86	3.86	4.20	4.20	4.20	4.20	4.20
Sample 3	285.96	273.75	275.71	274.73	275.71	275.71	275.71	274.73	273.75	274.73	275.71

Loss (%)	0.00	4.27	3.58	3.93	3.58	3.584	3.58	3.93	4.27	3.93	3.58
Mean (%)	0.00	4.12	3.78	3.78	3.78	3.66	3.78	4.00	4.12	4.00	3.78
Standard Deviation	0.0000	0.2033	0.3646	0.2039	0.1674	0.1711	0.3646	0.1676	0.2033	0.1676	0.3646

Table R5 | Raw data of Supplementary Fig. 24b.

<u>Replies to the Reviewers' Comments and Revisions Made to the Manuscript</u>

Reviewer 1:

1. The authors have taken into account the reviewers' numerous comments. The responses are well structured, address the questions, and improve the overall quality of the manuscript. A substantial amount of supplementary material has been produced, and the bibliography has been expanded. I have only one point to suggest for revision: the number of significant figures reported for some values. In several cases, the stated precision is unnecessary given the units, standard deviations, or number of measurements. Values with a higher number of significant figures may be presented in the Supplementary Material or in the raw data.

Response:

We sincerely thank the reviewer for the positive assessment of our revision and the constructive suggestion regarding data presentation. We agree that the reported precision should reflect the experimental error and valid significant figures. Accordingly, we have reviewed all data in the manuscript and adjusted the number of significant figures. As suggested, the values with higher precision have been moved to the Supplementary Material and Raw Data for reference.

Reviewer 2:

1.The authors have revised the manuscript as the reviewer commented. Hence, the revised manuscript is scientifically crystal clear. In this regard, the manuscript has novelty for publication in Nature Communications.

Response: We sincerely appreciate the reviewer's highly positive evaluation of our revised manuscript. We are encouraged that the reviewer recognizes the scientific clarity and the novelty of our work for publication in Nature Communications.

2.I examined the response to the comment from Reviewer 3. The answers are insufficient for the questions. The reason is as follows.

Response: We also sincerely thank the reviewer for their critical and meticulous assessment of our responses to Reviewer #3. We fully agree with the reviewer's judgment. To thoroughly address these concerns, we have performed a series of additional experiments and rigorous analyses as suggested below.

3. Answer 1. Chemical formula is needed. HLB values of other tritons in Table R3 should be checked again.

Response: Thanks for your detailed comments. The added chemical formula and revised HLB value are updated in Table R1 and Supplementary Table 3.

Triton Type	Chemical Formula	HLB Value
Triton X-15[1]	*tert-Octyl*-C_6H_4-$(OCH_2CH_2)_{1.5}OH$	4.9
Triton X-45[2]	*tert-Octyl* -C_6H_4-$(OCH_2CH_2)_{4.5}OH$	9.8
**Triton X-100**[3]	*tert-Octyl* -C_6H_4-$(OCH_2CH_2)_{9.5}OH$	**13.4**
Triton X-165[4]	*tert-Octyl* -C_6H_4-$(OCH_2CH_2)_{16}OH$	15.5
Triton X-305[5]	*tert-Octyl* -C_6H_4-$(OCH_2CH_2)_{30}OH$	17.3

Table R1 | Different HLB values of Triton series. HLB stands for Hydrophile-Lipophile Balance.

Reference

1. TRITON™ X-15 Surfactant | Dow Inc. https://www.dow.com/en-us/pdp.triton-x-15-surfactant.85740z.html#properties.

2. TRITON™ X-45 Surfactant | Dow Inc. https://www.dow.com/en-us/pdp.triton-x-45-surfactant.85765z.html?productCatalogFlag=1#properties.

3. TRITON™ X-100 Surfactant | Dow Inc. https://www.dow.com/en-us/pdp.triton-x-100-surfactant.85735z.html?productCatalogFlag=1#properties.

4. TRITON™ X-165 (70% Actives) Surfactant | Dow Inc. https://www.dow.com/en-us/pdp.triton-x-165-70-actives-surfactant.85743z.html#properties.

5. TRITON™ X-305 (70% Actives) Surfactant | Dow Inc. https://www.dow.com/en-us/pdp.triton-x-305-70-actives-surfactant.85759z.html#properties.

4. Answer 3. The FE-SEM or FE-TEM image of the cross-section of the single fiber with larger diameters and the electrical conductivity of the single fiber have to be provided.

Response: We appreciate your insightful comments. As shown in Figure R1a and R1b, we have performed FE-SEM to investigate conformal coaxial rGO coating of single PP fiber with larger diameter (D = 210.80 μm) by the same coating method. Clearly, the distinct wrinkles of rGO appear on the surface, indicating the coaxial rGO coating is successfully assembled. Moreover, the cross-section SEM image captured by Cryogenic Focused Ion Beam (Cryo-FIB-SEM) exhibits a conformal coaxial rGO coating of around 55 nm. Considering the micro-sized fibers (1–5 μm) typically found in the polypropylene melt-blown fabrics (PMF), the successful construction of a highly conformal coating on this 210.80 μm fiber unambiguously confirms the size universality of our coaxial coating strategy.

To measure the conductivity of the single fiber, linear sweep voltammetry (LSV) was conducted using a customized four-probe setup to effectively eliminate contact resistance (Figure R1c). As shown in the highly linear U-I curve (Figure R1d), the measured resistance (R) of the large-diameter fiber is 15,824.53 Ω between RE and Sensor. The apparent conductivity (σ_{app}) of large-diameter fiber is 20.28 S m^{-1}, calculated by the following formula:

$$\sigma_{app} = \frac{L}{R \cdot A_{total}} \quad (1)$$

Where:

σ_{app} is the conductivity in the z-direction;

L is the length between RE and Sensor;

R is the resistance of the large-diameter fiber between RE and Sensor;

A_{total} is the the total cross-sectional area.

Since the massive PP core is electrically insulating, we extracted the true intrinsic conductivity (σ_{int}) of the 55.15 nm rGO shell utilizing its effective cross-sectional area (A_{rGO}) by the following formula:

$$\sigma_{int} = \frac{L}{R \cdot A_{rGO}} \tag{2}$$

Where:

σ_{int} is the intrinsic conductivity of the conformal rGO shell;

A_{rGO} is the effective cross-sectional area of the rGO shell.

This yields an intrinsic conductivity of 19,378.55 S m^{-1}, resulting from our moderate chemical reduction. In PMF, the PP fibers possess much smaller diameters (D) ranging from 1 to 5 μm. By applying this intrinsic conductivity and the most conservative rGO thickness (t = 25 nm, Figure 2l) to these microfibers, their theoretical apparent conductivity (σ_{micro}) is calculated by the following formula:

$$\sigma_{micro} = \sigma_{int} \frac{4t}{D} \tag{3}$$

Where:

σ_{int} is the theoretical apparent conductivity of the microfiber in rGO-PMF-16;

t is the most conservative rGO coating thickness on the microfiber (25 nm);

D is the diameter of the microfiber in PMF (1–5 μm).

Through this scaling effect, the theoretical apparent conductivity of the fibers in rGO-PMF-16 achieves a lower bound of 387.57 (5 μm) to 1,937.86 S m^{-1} (1 μm). In traditional porous materials, macroscopic conductivity typically drops by 1 to 2 orders of magnitude due to massive contact resistance at fiber intersections. Remarkably, the macroscopic in-plane conductivity of rGO-PMF-16 reaches 283.06 S m^{-1}, demonstrating an ultra-high retention rate. This strongly supports that our observed rGO bridges and fiber bundles (Supplementary Fig. 1) act as effective welds, minimizing point-contact resistance and translating superior single-fiber performance

into a highly efficient 3D conductive network. The corresponding results are also included in the revised Supplementary Fig. 3 and 21.

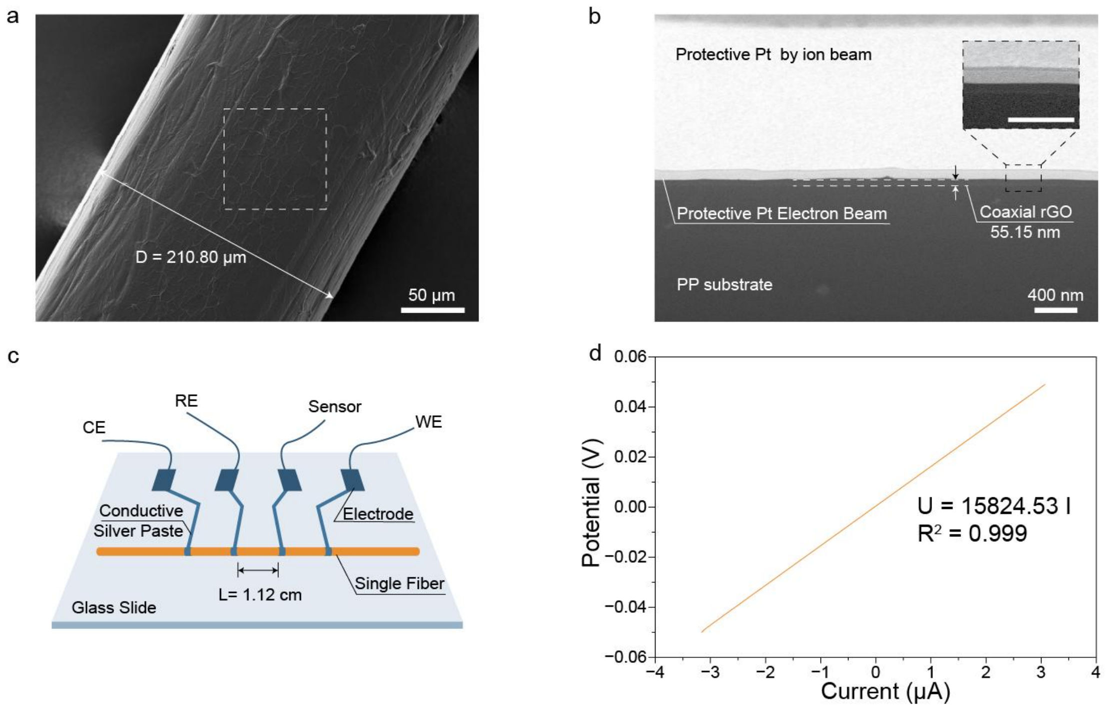

Figure R1 | Characterization of large diameter single fiber's surface, cross-section and its conductivity. a, The SEM image of the surface of the rGO coated large diameter fiber. **b,** The SEM image of the cross-section of the rGO coated large diameter fiber. **c,** Schematic diagram of the revised four probe conductivity measurement setup (CE represents Counter Electrode; RE represents Reference Electrode; Sensor represents Sensing Electrode, and WE represents Working Electrode). **d,** U-I curves from the LSV results.

5. Answers 5 and 7. The most important thing is that the conductivity obtained from the sheet resistance with the thickness can not interpret the conductive pathway along the z-direction. It is just a conventional 4-probe measurement. This method just provides in-plane conductivity.

Response: We thank the reviewer for this valuable comment regarding the electron transport mechanism. We agree that the conductivity derived from standard 4-probe measurements primarily characterizes the efficient in-plane transport along the continuous fiber network. To explicitly quantify the vertical transport and validate the existence of the 3D conductive pathway, we performed z-direction conductivity

measurements using a customized setup in LSV mode of an electrochemical workstation (Figure R2a). The sample was sandwiched between two copper electrodes under a constant load (500 g, yielding a pressure of 15.6 kPa). This specific pressure is deliberately applied to ensure reliable ohmic contact while strictly preserving the fabric's intrinsic highly porous architecture. Crucially, this value falls well within the real operational pressure range of wearable electronics on human skin, distinct from the destructive high-pressure testing (typically >1 MPa) conventionally used for rigid industrial materials like carbon paper. The internal resistance was baseline-corrected (Figure R2b). The net z-direction resistance (R_Z) of the rGO-PMF-16 is 0.05 Ω. Based on the actual thickness and the contact area of the sample, the z-direction conductivity (σ_Z) is 8.28 S m^{-1} which is calculated by the following formula:

$$\sigma_Z = \frac{h}{R_Z \cdot A} \tag{4}$$

σ_Z is the conductivity in the z-direction;

h is the actual thickness of the rGO-PMF-16 under the applied load;

R_Z is the net z-direction resistance of the rGO-PMF-16 (0.05 Ω);

A is the contact area of the Cu electrodes (3.14 cm^2).

This result reveals a significant but expected anisotropy compared to the in-plane conductivity (283.06 S m^{-1}). This behavior, caused by non-woven architecture (characterized by randomly oriented fibers stacked in the planar direction rather than an ordered interlaced structure), is consistent with the intrinsic physics of fibrous assemblies. Similar to carbon paper and carbon cloth[6,7], electron transport is naturally more efficient along the continuous fiber axis (in-plane) than hopping across fiber-to-fiber junctions in the thickness direction (z-direction). Crucially, the existence of a measurable z-direction conductivity strongly supports our claim of coaxial coating and bundle structures. In the absence of a conformal coating penetrating the entire fabric depth, the z-direction would show ultra-low conductivity or even remain electrically insulated. The ability to conduct current through the thickness validates the formation of a fully interconnected 3D conductive network, despite the anisotropic efficiency. The corresponding results are also included in the revised Supplementary Fig. 22.

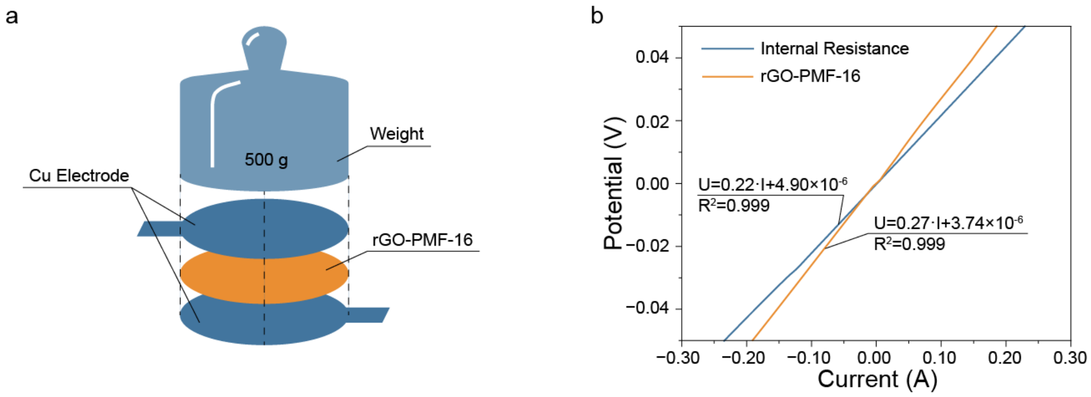

Figure R2 | Characterization of rGO-PMF-16's z-direction conductivity. a, Schematic diagram of the z-direction conductivity measurement setup. The sample is clamped between two Cu electrodes under a 500 g weight (~15.6 kPa). **b,** U-I curves from the LSV results.

Reference

6. Toray Carbon Paper 060, Wet Proofed. https://www.fuelcellstore.com/toray-carbon-paper-060.

7. Wang, Y., Wang, C.-Y. & Chen, K. S. Elucidating differences between carbon paper and carbon cloth in polymer electrolyte fuel cells. *J. Electacta.* **52**, 3965–3975 (2007).

6. The conductivity is over 10^3 S/m in Figure 5d, which is not consistent with answer 7 (283.6 S/m).

Response: We appreciate the reviewer's careful observation. The conductivity value of >10^3 S m^{-1} in Figure 5d corresponds to the sample reduced by HI acid (rGO-PMF-16_{HI}, 1214.84 S m^{-1}), whereas the value of 283.06 S m^{-1} mentioned in Answer 7 refers to the standard sample (rGO-PMF-16). To avoid confusion, we have explicitly labeled the specific sample names and their corresponding reduction methods in the revised Figure 5d.

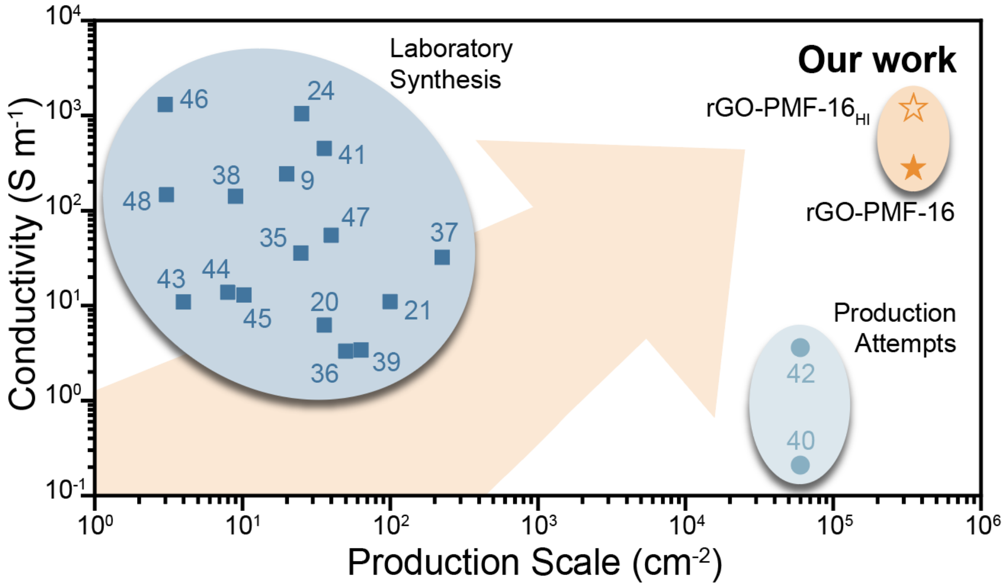

Figure 5d | The comparison of graphene-based fabrics in conductivity and production scale.

In SI

1. Answer 2. How to measure total weight loss? If the authors used the scale, the results are not acceptable because a small amount of the nanoparticles can affect the environment.

Response: We fully agree with the reviewer that weight analysis alone is insufficient to detect trace amounts of nano-pollutants. Therefore, we rigorously addressed this concern by implementing a multi-dimensional assessment strategy that combines spectroscopic analysis (Dynamic Light Scattering, DLS), morphological characterization (Scanning Electron Microscopy, SEM), and a worst-case theoretical exposure model (Predicted Environmental Concentration, PEC).

1. Experimental Verification: Absence of Suspended Nanoparticles

To specifically detect potentially released nanoparticles that a scale would miss, we analyzed the washing supernatant using DLS and SEM. As presented (Figure R3a), the volume-weighted particle size distribution of the washing solution is spectroscopically identical to that of the pure detergent background. Both samples exhibit dominant peaks at ~80 and ~420 nm, corresponding to insoluble fillers (e.g., zeolites) inherent to the detergent matrix, with no additional signals detected in the nano-range. This confirms that the rGO nanosheets (Average size = 401.53 nm) did not release detectable

suspended nanosheets. Corroborating this, SEM analysis of the filtration residue (Figure R3b) identifies the physical source of the mass loss (~2.3%) as micron-sized fibers detached during mechanical agitation. Furthermore, enlarged SEM image in Figure R3c reveals that the rGO coating remains firmly anchored to these microfibers rather than peeled off. Thus, the weight loss represents rapid-settling macro-particulates rather than persistent nano-pollutants.

2. Theoretical Safety Net: Worst-Case PEC Calculation (Per Garment)

To quantitatively bound the environmental risk, we performed a PEC calculation following ECHA Guidance[8], modeling the daily washing of a single functional T-shirt (assumed area: 1 m^2). We adopted an extreme "worst-case" assumption where the entire gravimetric mass loss (2.3%) is treated as 100% pure rGO, intentionally disregarding the fiber content. Even under this severe overestimation and applying a standard 1% market penetration[9] factor for emerging functional textiles and 95% wastewater treatment plant (WWTP) removal efficiency[10], the calculated PEC is 0.19 ppb. This theoretical upper value is significantly lower than the suggested 0.63 ppb[11] safety threshold, confirming that the environmental risk is negligible. Calculation details are listed with reference (Table R2).

Consequently, both experimental evidence (DLS/SEM) and the worst-case theoretical model demonstrate that the mass loss detected by the scale does not translate into hazardous nanoparticle release. The corresponding results are also included in the revised Supplementary Fig. 28 and Supplementary Table 9.

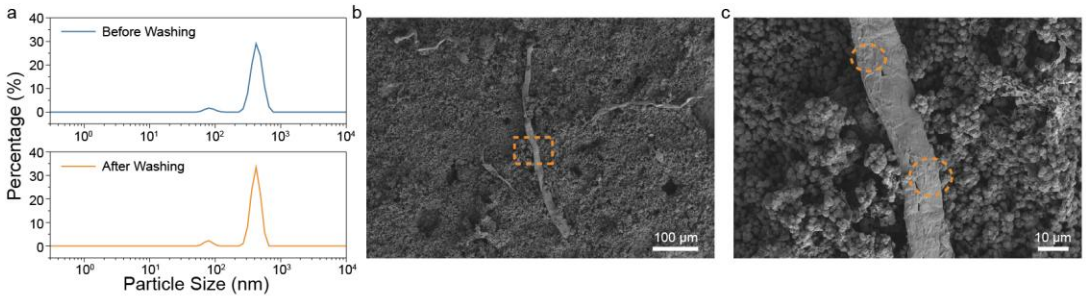

Figure R3 | Characterization of rGO-PMF-16 properties. a, The volume percentage-particle size curves of the washing supernatant before and after washing. **b,** SEM images of the filter material on

a 0.22 micrometer filter membrane collected from the washing processes. **c,** The zoom-in of the marked part in b.

Parameter	Value	Reference
Fabrics Weight	32.75 g m^{-2}	Actual Measured
Garment Area	1.0 m^2	Commercial Average
Total Weight Loss	2.3%	Actual Measured
Assumed Release	753 mg $item^{-1}$	Calculated
Daily Sewage Volume	200 L day^{-1}	[8]
Market Penetration	1%	[9]
WWTP Removal	95%	[10]
River Dilution	10	[8]
Safety Threshold	0.63 ppb	[11]
Calculated PEC	0.19 ppb	Calculated

Table R2 | Worst-case parameters and calculation of PEC.

Reference

8. Guidance on information requirements and Chemical Safety Assessment Chapter R.16: Environmental exposure assessment. https://echa.europa.eu/documents/10162/17224/IR_CSR_R16_V4_FINAL.pdf/b9f0f406-ff5f-4315-908e-e5f83115d6af (2026).

9. Committee for Medicinal Products for Human Use (CHMP) Guideline on the environmental risk assessment of medicinal products for human use. https://www.ema.europa.eu/en/documents/scientific-guideline/guideline-environmental-risk-assessment-medicinal-products-human-use-revision-1_en.pdf (2024).

10. Registry of restriction intentions until outcome - ECHA. https://echa.europa.eu/registry-of-restriction-intentions/-/dislist/details/0b0236e18244cd73.

11. Fekete-Kertész, I. *et al.* Assessing the Chronic Environmental Risk of Graphene Oxide Using a Multimarker Approach Across Three Trophic Levels of the Aquatic Ecosystem. *Nanomaterials* **15**, 1553 (2025).

2. Answer 3. I agree with Reviewer 3. The author should perform on macrophages.

Response: We strictly appreciate the reviewer's suggestion. We agree that evaluating the interaction with immune cells is critical for ensuring the biosafety of wearable skin-electronics. To thoroughly address this, we conducted biocompatibility tests using macrophages co-cultured with PMF, rGO-PMF-16, and rGO-PMF-16$_{HI}$. We performed both quantitative cell viability assays (up to 72 h) and qualitative Live/Dead fluorescence staining (Figure R4-R6).

As shown in Figure R4 and R6a, the quantitative results demonstrate that macrophages maintained a high survival rate (near 100%) across all groups after 24, 48, and 72 hours of incubation, showing no significant difference compared to the control. Furthermore, fluorescence microscopy images (Calcein-AM/PI staining, Figure R5 and R6) reveal a high density of viable cells (green fluorescence) with negligible dead cells (red fluorescence). The cells displayed healthy spreading morphology, indicating excellent proliferation behavior.

These combined results confirm that the rGO-coated fabrics exhibit negligible cytotoxicity to immune cells, strongly supporting their biocompatibility and safety for long-term skin-contact applications. The corresponding results are also included in the revised Supplementary Fig. 32-34.

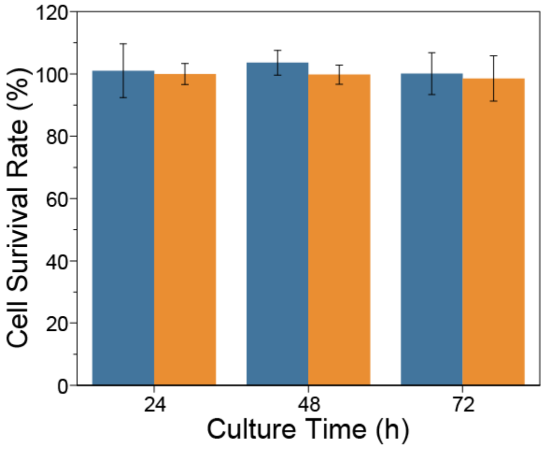

Figure R4 | The cell survival rate of PMF and rGO-PMF-16 in macrophages test.

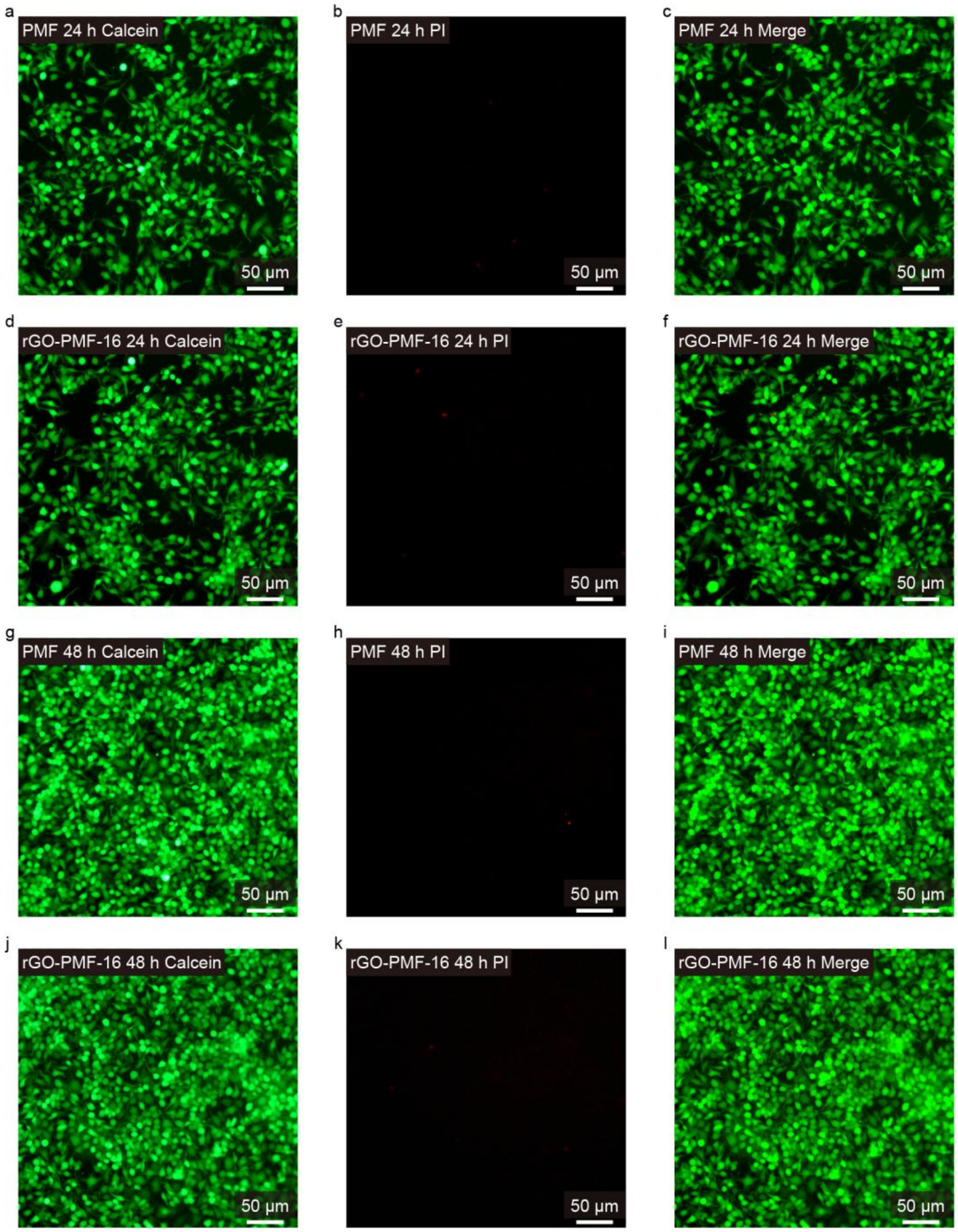

Figure R5 | The fluorescence microscopy image of macrophages cultivated with PMF and rGO-PMF-16. The calcein fluorescence microscopy image of macrophages cultivated with PMF in **a)** 24 and **g)** 48h. The propidium iodine (PI) fluorescence microscopy image of macrophages cultivated with PMF in **b)** 24 and **h)** 48h. The merge fluorescence microscopy image of macrophages cultivated with PMF in **c)** 24 and **i)** 48h. The calcein fluorescence microscopy image of macrophages cultivated with rGO-PMF-16 in **d)** 24 and **j)** 48h. The propidium iodine (PI) fluorescence microscopy image of macrophages cultivated with rGO-PMF-16 in **e)** 24 and **k)** 48h.

The merge fluorescence microscopy image of macrophages cultivated with rGO-PMF-16 **f)** 24 and **l)** 48h.

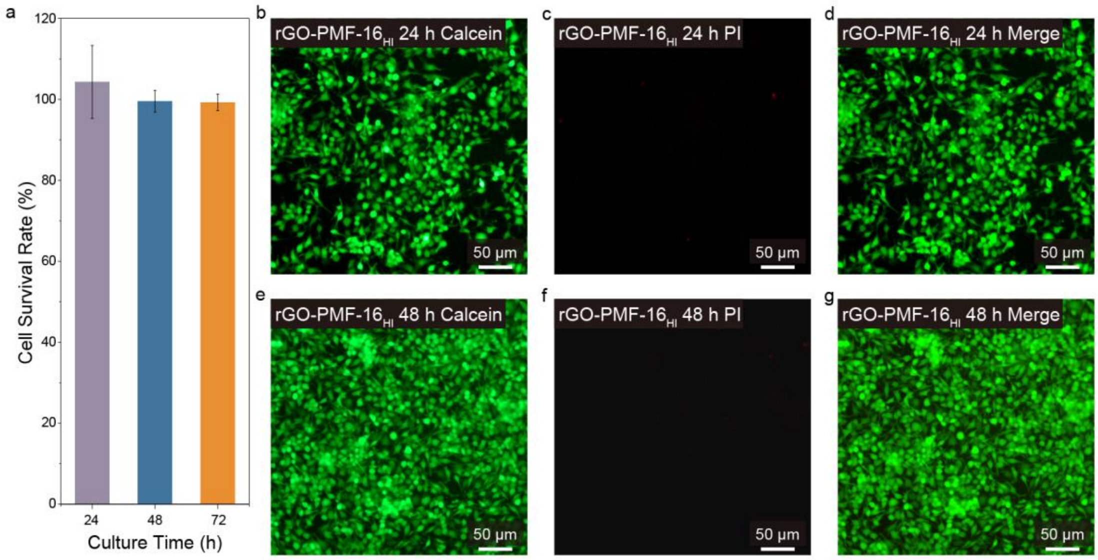

Figure R6 | The cell survival rate of rGO-PMF-16$_{HI}$ and the fluorescence microscopy image of macrophages cultivated with rGO-PMF-16$_{HI}$. a, The cell survival rate of rGO-PMF-16$_{HI}$. The calcein fluorescence microscopy image of macrophages cultivated with rGO-PMF-16$_{HI}$ in **b)** 24 and **e)** 48h. The propidium iodine (PI) fluorescence microscopy image of macrophages cultivated with rGO-PMF-16$_{HI}$ in **c)** 24 and **f)** 48h. The merge fluorescence microscopy image of macrophages cultivated with rGO-PMF-16$_{HI}$ **d)** 24 and **g)** 48h.
